# Alphaflexiviridae in Focus: Genomic Signatures, Conserved Elements and Viral-Driven Cellular Remodeling

**DOI:** 10.3390/v17050611

**Published:** 2025-04-24

**Authors:** Jesús R. Úbeda, Miguel A. Aranda, Livia Donaire

**Affiliations:** Centro de Edafología y Biología Aplicada del Segura (CEBAS)-CSIC, Department of Stress Biology and Plant Pathology, P.O. Box 164, Espinardo, 30100 Murcia, Spain; jerodriguez@cebas.csic.es (J.R.Ú.); m.aranda@cebas.csic.es (M.A.A.)

**Keywords:** *Allexivirus*, *Lolavirus*, *Platypuvirus*, *Potexvirus*, *Botrexvirus*, *Sclerodarnavirus*, replicase, coat protein, triple gene block proteins

## Abstract

The family *Alphaflexiviridae* comprises plant- and fungus-infecting viruses with single-stranded, positive-sense RNA genomes ranging from 5.4 to 9 kb. Their virions are flexuous and filamentous, measuring 470–800 nm in length and 12–13 nm in diameter. The family includes 72 recognized species, classified into six genera: *Allexivirus*, *Lolavirus*, *Platypuvirus*, *Potexvirus* (plant-infecting), and *Botrexvirus* and *Sclerodarnavirus* (fungus-infecting). The genus *Potexvirus* is the largest, with 52 species, including *Potexvirus ecspotati* (potato virus X), an important crop pathogen and plant virology model. The genera are distinguished by genome organization and host range, while species differentiation relies on nucleotide and protein sequence identity thresholds. In this review, we summarize the current knowledge on the genomic structure, conserved genes, and phylogenetic relationships within *Alphaflexiviridae*, with a particular focus on the replicase and coat protein genes as signature markers. Additionally, we update the model of cellular remodeling driven by the triple gene block proteins, which are essential for virus movement, among other viral functions. Beyond their biological significance, alphaflexiviruses serve as valuable models for studying virus–host dynamics and hold potential applications in plant disease control and biotechnology. This review provides an updated framework for understanding *Alphaflexiviridae* and their broader impact on plant virology.

## 1. Introduction

According to the most recent virus megataxonomy proposed by the International Committee on Taxonomy of Viruses (ICTV), the family *Alphaflexiviridae* is taxonomically placed within the kingdom *Orthornavirae*, phylum *Kitrinoviricota*, class *Alsuviricetes*, and order *Tymovirales*, under the realm *Riboviria* [1]. Viruses in the family *Alphaflexiviridae* have single-stranded, positive-sense RNA genomes ranging from 5.4 to 9 kb in length. They possess flexuous filamentous virions that are 470–800 nm long and 12–13 nm in diameter. The family includes 72 currently recognized species that are considered to have narrow host ranges and to infect plants or fungi that infect plants. Alphaflexiviruses that infect plants belong to four genera: *Allexivirus*, *Lolavirus*, *Platypuvirus*, and *Potexvirus*. Alphaflexiviruses isolated from fungi belong to two genera: *Botrexvirus* and *Sclerodarnavirus*. The genus *Potexvirus* is the largest in the family, currently comprising 52 species; it includes (and is named after) *Potexvirus ecspotati* (species formerly known as *Potato virus X*), an important crop pathogen and a major model for plant virology [2,3]. Within the genera *Allexivirus* and *Potexvirus*, the subgenera *Acarallexivirus* and *Mandarivirus* have been defined, respectively. Genera within the family are delimited based on genome organization and host, while viruses from different species should have less than 72% nucleotide identity (or 80% amino acid identity) between their respective coat protein (CP) or replicase genes (or proteins). The aim of this review is to provide a comprehensive and up-to-date synthesis of the molecular and evolutionary biology of the family *Alphaflexiviridae*, based on the latest classification and nomenclature established by the ICTV. In the following paragraphs, we review the genome structure of the viruses in the family, the genes that are conserved across genera, and the phylogenetic relationships that can be inferred by considering the alphaflexivirus signature proteins, replicase and CP. We have illustrated the contents of the following sections by using one exemplar virus of each genus and subgenus (Table S1), while for the rest of the analyses, we have used the sequences of exemplar isolates in all 72 alphaflexivirus species (Table S2). In addition, we updated the model for the cellular remodeling driven by the *Alphaflexiviridae* triple gene block proteins.

## 2. The *Alphaflexiviridae* Genomes

**Figure 1:**
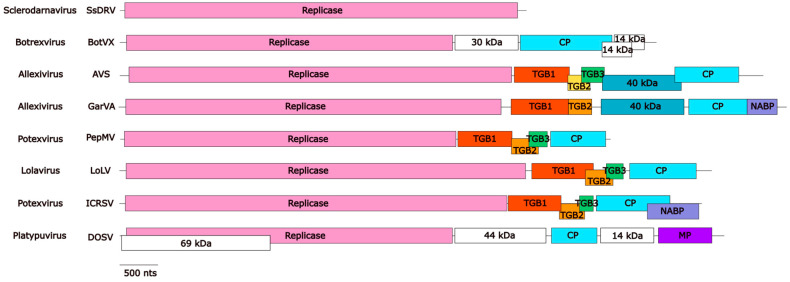
Genome organization of exemplar viruses within each genus and subgenus of the family *Alphaflexiviridae*. 5′ and 3′ untranslated regions are represented by a black line. Boxes in different colors mark regions encoding known viral proteins; regions encoding hypothetical proteins or proteins with unknown function are marked with white boxes. TGB: triple gene block; CP: coat protein; NABP: nucleic acid binding protein; MP: movement protein. A scale bar of 500 nucleotides is shown.

**Figure 2:**
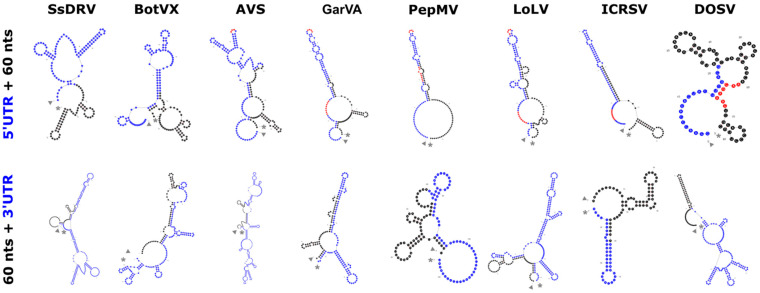
5′ and 3′ untranslated regions (UTRs) predicted structures of exemplar viruses within each genus and subgenus of the family *Alphaflexiviridae*. RNA structures include the 5′ and 3′-UTRs plus 60 nts downstream of the translation initiation codon, or upstream of the termination codon, respectively. The structure with the lowest initial Gibbs free energy (DG) was selected using the UNAFold web server. The UTR sequences are highlighted in dark blue. The positions of the putative conserved octanucleotide (ACCNNACC) and the GAAA sequence in the 5′ stem-loop 1 (5′SL1) are highlighted in red. 5′ end is denoted with a triangle and 3′ end with an asterisk.

The alphaflexivirus genome (Figure 1) is polyadenylated at its 3′ end, and in all cases studied, it contains a cap structure at the 5′ end consisting of a 7-methylguanosine (m7G). There are untranslated regions (UTRs) at both ends of the genome of around 80 nt for the 5’-UTR and 150 nt for the 3′-UTR, although there is extensive length variability for these regions (Table S1). Both UTRs contain RNA structured elements, including stem loops (SL) that often extend into the coding regions, with a more pronounced complexity in the 3′-UTR (Figure 2). Some of these elements have been well studied for particular viruses in the family; for instance, the multifunctional SL1 in the potato virus X (PVX) 5’-UTR has been shown to participate in plus-strand RNA accumulation during viral replication [4], CP binding and virion assembly [5], as well as interaction with host factors [6], and positional equivalents can be identified for other members of the family (Figure 2 and Figure S1). Similarly, SLs in the PVX 3′-UTR have been shown to affect minus and plus-strand viral RNA accumulation differentially [7], and a conserved 5′-apical hairpin stem loop [8], as well as a pseudoknot in the 3′-UTR that interacts with the viral replicase [9], are required for efficient bamboo mosaic virus (BaMV) (*Potexvirus bambusae*) replication. Interestingly, 5′- and 3′-UTRs of satellite RNAs of BaMV (satBaMVs) have evolved similar RNA secondary structures and functional RNA elements with BaMV, including GAAA(A) repeats at the 5′-UTR, conserved hexanucleotides (ACCUAA) and polyadenylation signals (AAUAAA) at the 3′-UTR, as well as three stem loops at the 3′-UTR that are structurally conserved in the BaMV 3′-UTR, pointing toward RNA structural requirements for the BaMV replicase recognition [10], not excluding other satBaMV specific functions [11]. In contrast with these examples, RNA elements in the 5′- and 3′-UTRs of most other viruses in the family remain poorly characterized, and in many cases, putative functions are assumed based on their positional homology with the well-studied examples.

The alphaflexivirus replicase is encoded in the first and longest open reading frame (ORF) of the genome (Figure 1), with the exception of donkey orchid symptomless virus (DOSV) (*Platypuvirus asinorchis*), the only virus characterized thus far within its genus (see below). Following ORF1, there is a variable number of validated or hypothetical ORFs, ranging from none to five; their number and nature is a distinguishing feature of the genera within the family (Figure 1 and Table S2). *Sclerotinia sclerotiorum* debilitation-associated RNA virus (SsDRV) (*Sclerodarnavirus sclerotiniae*), the only virus in its genus, has the shortest genome, and no other ORF follows ORF1 (Figure 1 and Table S2). Viruses in the genus *Botrexvirus*, the other group of alphaflexiviruses that infect fungi, have three to four ORFs after the first one, which encode hypothetical proteins of unknown functions except for the CP (Figure 1 and Table S2). Genera *Allexivirus*, *Lolavirus,* and *Potexvirus*, the three of them being viruses that infect plants, have genomes structured in a similar manner (Figure 1 and Table S2); following ORF1, there is a conserved block of genes known as the triple gene block (TGB), with TGB1 being described as a multifunctional protein involved in virus movement, RNAi suppression, and formation of virus replication organelles (VROs), among other functions, and the small TGB2 and TGB3 as integral membrane proteins responsible for vesicle induction and required for virus movement [12,13] (see below). In the case of viruses in the genus *Allexivirus*, only three of them have a canonical TGB3 annotated in their genomic sequences, although TGB3s in this group may not contain a canonical initiation codon and may be translated by leaky scanning [14]. Also, for allexiviruses, TGB ORFs are followed by an ORF encoding a 40 kDa hypothetical protein conserved within the genus. The CP ORF follows, and there is a final ORF in the genomes of viruses of the two accepted subgenera encoding a cysteine-rich protein that is variable in size, with nucleic acid binding capacity (Figure 1) (see below).

DOSV has, by far, the more divergent genome among alphaflexiviruses in structural terms (Figure 1 and Table S2). To begin, the genomic RNA is non-polyadenylated; instead, the 3′-UTR is predicted to contain a pseudoknot resembling a transfer RNA-like (tRNA) secondary structure. The DOSV replicase is encoded in ORF2, with ORF1 encoding a hypothetical protein of 69 kDa containing sequences conserved among tymovirus movement proteins and ORF6 encoding a movement protein belonging to the 30K superfamily class [15], with similarity to the 3A movement proteins of diantho and furoviruses. Therefore, the DOSV movement function seems to be coded by ORFs 1 and 6, which are distantly positioned in the genome. To continue, the DOSV ORF3 encodes a hypothetical protein with similarity to autophagy protein 16 from squirrelpox virus. DOSV ORF5 can be translated into a 27 kDa hypothetical protein with no known homology, whereas ORF4 encodes a CP with a clear similarity to allexivirus CPs. The peculiarity of the DOSV genome perhaps reflects modular evolution in a very particular niche, but its replicase and CP clearly position this virus within the family *Alphaflexiviridae* [16] -(NCBI database, RefSeq NC_022894.1) (see below).

The alphaflexivirus genomic RNA serves as messenger for the replicase, while downstream ORFs are translated from subgenomic RNAs (sgRNAs). sgRNAs are 3′-co-terminal, include a 3′-terminal poly(A) tail (excluding DOSV), are supposed to be 5′-capped with a covalently linked m7G, and may (or may not) be encapsidated by the CP. Internal and 3′-terminal ORFs have upstream sequences with validated or hypothesized RNA structural elements with a role in sgRNAs synthesis and/or expression (see, for instance, [17]). sgRNAs can be polycistronic, and leaky scanning appears to be the mechanism for downstream ORF translation (e.g., [18]).

## 3. The Family Signature: Replicase and Coat Proteins

**Figure 3:**
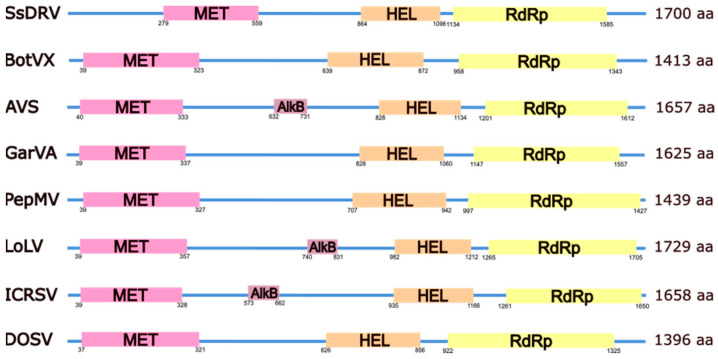
Organization of replicase motifs of exemplar viruses within each genus and subgenus of the family *Alphaflexiviridae*. Coordinates of each motif refer to amino acid positions. The amino acid length of each replicase protein is shown on the right. MET: type 1 RNA methyltransferase; HEL: RNA helicase-like; RdRp: RNA-dependent RNA polymerase [30].

**Figure 4:**
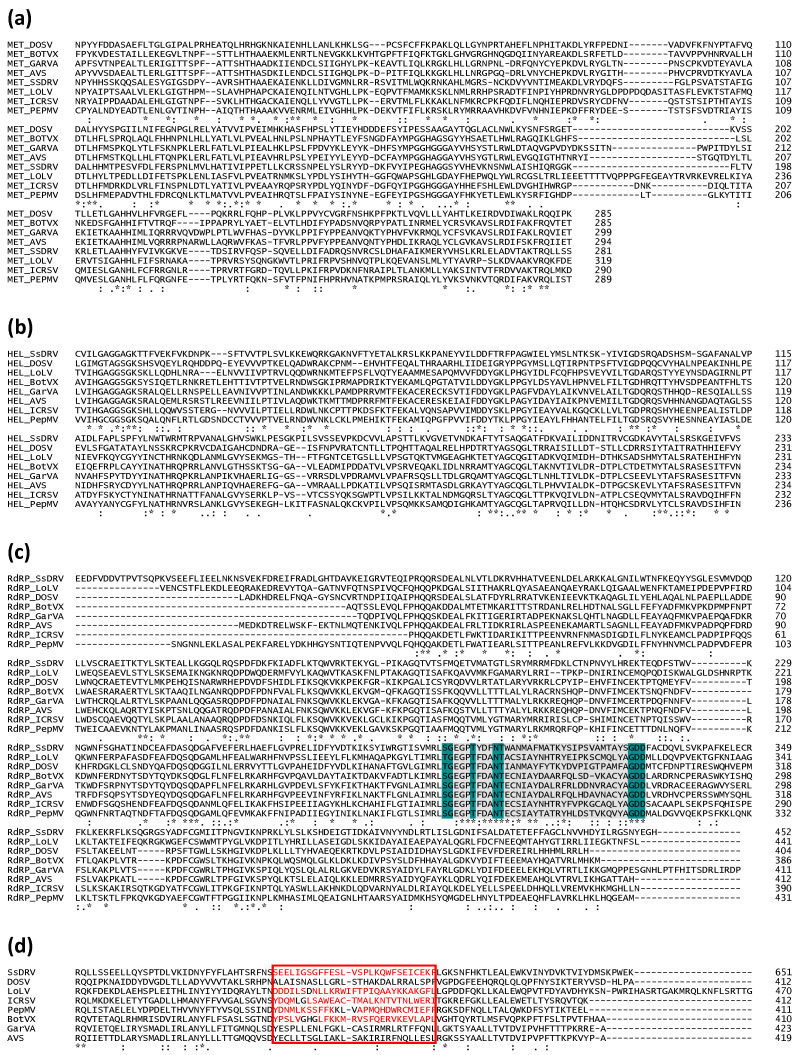
Amino acid alignments of replicase motifs of exemplar viruses within each genus and subgenus of the family *Alphaflexiviridae*. Amino acid alignments of MET (**a**), HEL (**b**), and RdRp (**c**) motifs. The characteristic core motif S/TGX3TX3NS/TX22GDD is highlighted in (**c**), where conserved amino acids are in dark green and any amino acid (X) in grey. (**d**) Amino acid alignment showing the predicted location of the amphipathic alpha-helix downstream of the MET domain. The red square indicates the location of the alpha-helix in Plantago asiatica mosaic virus (*Potexvirus marmorplantagonis*), and the red letters indicate the positions with predicted helical structure as in [30]. (*) Fully conserved residues; (:) Conservation between groups with strongly similar properties; (.) Conservation between groups with weakly similar properties.

Alphaflexivirus replicases range between 121.1 and 196.2 kDa in size and contain orderly arranged type 1 RNA methyltransferase (MET), RNA helicase-like (HEL), and RNA-dependent RNA polymerase (RdRp) motifs (Figure 3). MET is proximal to the N terminus of the protein and is part of the replicase capping domain. This domain is structurally homologous to those found in bromo mosaic virus replicase protein 1A, tobacco mosaic virus P126, and hepatitis E virus p110, which together with the equivalent in BaMV replicase, have properties in common with the guanine-N7-methyltransferase and the guanylyltransferase (GTase) of alphaviruses [19]. Functionally, this domain is involved in the capping of viral mRNAs; evidence generated using the BaMV replicase capping domain expressed in yeast led to the proposition that it works as a S-adenosylmethionine (AdoMet)-dependent mRNA GTase in the following sequential steps: (1) Production of m7GTP after GTP and AdoMet binding to the capping domain, with the release of S-adenosyl-L-homocysteine. (2) Formation of a covalent m7GMP-enzyme intermediate, with pyrophosphate release. (3) Binding of the nascent diphosphate RNA to the m7GMP moiety, leading to the formation of the m7G RNA [20,21,22]. Interestingly, it has also been shown that the HEL domain of the BaMV replicase exhibits RNA 5′-triphosphatase activity; therefore, it is able to catalyze the removal of the gamma-phosphate at the 5′ terminus of the nascent positive-strand RNA [23], producing the diphosphate-RNA needed for step (3) described above, thus contributing to the capping of the viral RNA. As far as we are aware, no helicase activity has been demonstrated for the HEL domain of alphaflexiviruses. Therefore, it appears that the BaMV replicase encodes, in its MET and HEL domains, the main enzymatic activities required for the 5′cap formation of the viral RNA [24]; whether this distinct pathway can be generalized to all alphaflexivirus replicases awaits confirmation, but the conservation of critical amino acid residues in the MET and HEL domains (Figure 4a,b) strongly suggests so.

The RdRp domain of the *Alphaflexiviridae* replicases lies within the carboxi-terminal portion of the protein and includes the characteristic core motif S/TGX3TX3NS/TX22GDD [25] (Figure 4c). For BaMV, it has been shown that its RdRp recognizes an RNA 3′-terminal pseudoknot and that this interaction is required for minus strand viral RNA synthesis [9]. Other RNA structures in the terminal regions of potexviral plus or minus RNA strands may be recognized by RdRp and required for the initiation of viral RNA transcription [7,26,27].

In addition to the canonical MET, HEL, and RdRp motifs, alphaflexiviruses replicases may (or may not) contain an AlkB motif (Figure 3). AlkB proteins are iron(II)-and 2-oxoglutarate-dependent dioxygenases that reverse methylation damage, such as 1-methyladenine and 3-methylcytosine, in RNA and DNA. The AlkB motif is present as part of the replicase between MET and HEL. Its functionality has been demonstrated for plant viruses outside the family *Alphaflexiviridae* [28]. Interestingly, bioinformatics analyses suggested a striking association of AlkBs with phloem-limited viruses that infect perennial plants and also AlkB domain acquisition by horizontal transfer; these hold true for alphaflexiviruses [29]. Apart from the domains marked by specific motifs, it appears that alphaflexivirus replicases contain regions sharing structural homology interspersed among the major domains. This is the case of a proline-kinked amphipatic alfa-helix found conserved among alphaflexiviruses; it is placed downstream from the MET domain (Figure 4d). Experimental evidence obtained with plantago asiatica mosaic virus (*Potexvirus marmorplantagonis*) showed that it is a membrane association determinant that likely contributes to the replicase complex anchorage to membranes [30].

The other signature protein encoded by the alphaflexivirus genome is the CP, which ranges between 20.7 and 63.3 kDa in size. Two major deviations to the general model of alphaflexivirus CPs (and the viral particle that it contributes to build) include SsDRV and lolium latent virus (LoTV) (*Lolavirus latenslolii*). In the case of SsDRV, its genome does not code for a CP. LoTV expresses and incorporates two carboxy co-terminal forms of the CP into virions. The two forms of the CP are virion constituents in equimolar proportions. The N-terminal sequence of the larger LoTV CP includes a putative chloroplast transit peptide, potentially responsible for cleavage to yield the shorter CP version [31]. The interaction of the larger CP form with a host protein appears to redirect the larger CP to the chloroplast and modulate virus infection [32]. In general terms, there is large variation in the N-termini of the *Alphaflexiviridae* CPs; for instance, the Rehmannia allexivirus (ReAV) (*Allexivirus rehmanniae*) CP, which is larger than most allexivirus CPs [33], has more than 300 extra amino acids at its N-terminus with no known homology to any other alphaflexivirus CP (Figure S2). In contrast, when N-termini are excluded, the conservation of the CPs among *Alphaflexiviridae* members is striking (Figure S2). To our knowledge, the virion 3D structure of *Alphaflexiviridae* members has been solved to atomic or near-atomic resolution for five potexviruses: BaMV, papaya mosaic virus (*Potexvirus papayae*), pepino mosaic virus (PepMV) (*Potexvirus pepini*), Althernantera mosaic virus (*Potexvirus alternantherae*), and PVX. The structures of the viral particles and the CP protomers in virions are essentially coincident for these five viruses and show a significant resemblance with other flexuous-filamentous viruses [34,35,36,37,38]. In the virion, the repeating unit is formed by 8.8 CP protomers, arranged to form a left-handed helix with a helical pitch of approximately 35 Å and a diameter of about 130 Å. Each CP protomer comprises three domains: a short N-terminal domain (domain I); a central core domain (domain II); and a carboxy-terminal domain (domain III). Domain I protrudes from the CP core and, in the assembled virion, embraces domain II of a nearby CP. Domains I and II of the CP are exposed on the external surface of the virus particle, while domain III forms an internal tunnel of about 16 Å in diameter running through the center of the virion. Each CP subunit interacts side by side with two immediately adjacent subunits and with subunits present in the lower and upper segments of the helical arrangement. In the central tunnel, there are a number of amino acid residues that are well conserved among *Alphaflexiviridae* members and form an RNA binding pocket that stabilizes and protects the RNA within virions [34,35,36,37,38]. For PepMV, it has been proposed that a cysteine conserved residue, which lies within the RNA binding pocket, is susceptible to modification through oxidation, and this may contribute to regulating the PepMV CP functions including RNA encapsidation [39]; this functional shift may associate to a dynamic redistribution of the protein between the VRO and the cytoplasm. The CP of the studied alphaflexiviruses also exhibits nuclear localization [40].

**Figure 5:**
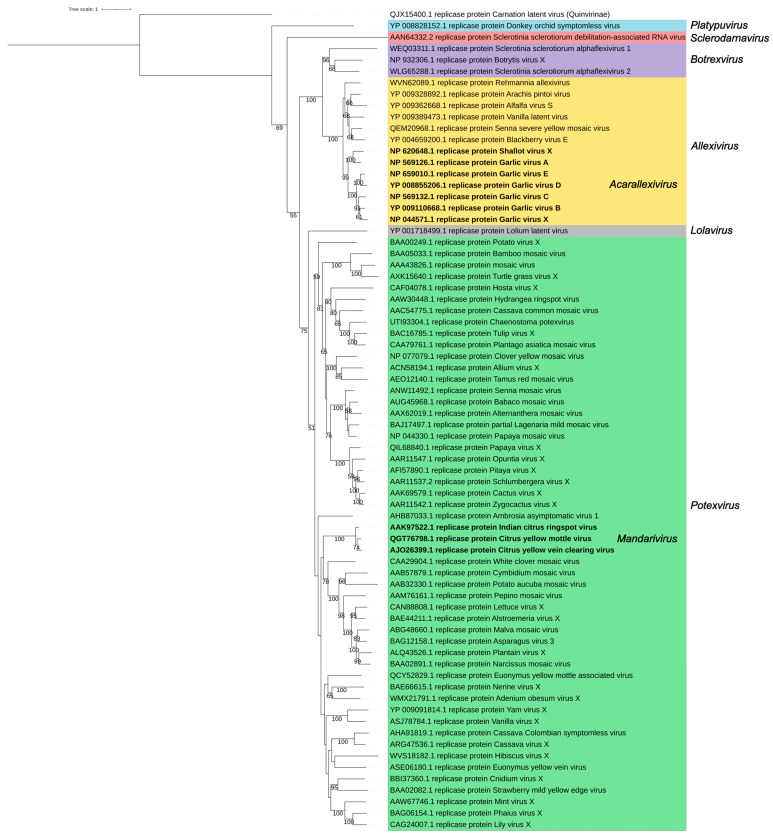
Phylogenetic analysis of the replicase protein of the family *Alphaflexiviridae*. The multiple sequence alignment of 72 replicase sequences was performed using MUSCLE [41]. The best amino acid substitution method was inferred using MEGA 11 [42]. The maximum likelihood (ML) trees were inferred using RAxML-NG software [43] using the LG method considering the proportion of invariable sites (+I) and the variation of the substitution rate among sites according to a gamma distribution (+G). The best ML tree with bootstrap support values (1000 replicates) is shown. Only bootstrap values higher than 50% are displayed. Carnation latent virus (QJX15400.1) (*Carlavirus latensdianthi*, family *Quinivirinae*) was used as an outgroup, and the tree was rooted with this sequence.

**Figure 6:**
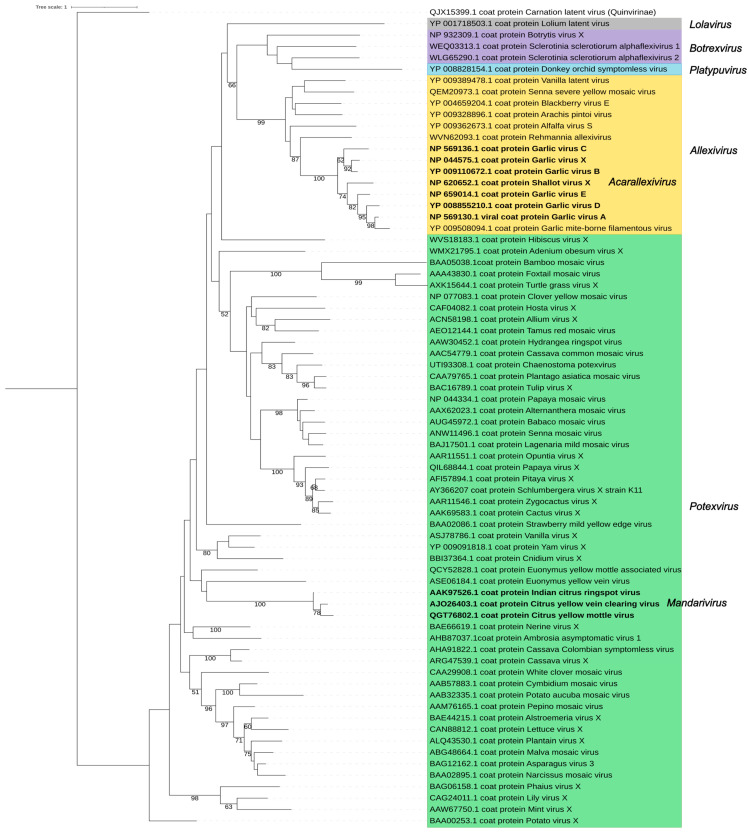
Phylogenetic analysis of full protein sequences of the coat protein (CP) of the family *Alphaflexiviridae*. The multiple sequence alignment of 72 CP sequences was performed using MUSCLE [41]. The best amino acid substitution method was inferred using MEGA 11 [42]. The maximum likelihood (ML) trees were inferred using RAxML-NG software [43] using the LG method considering the variation of the substitution rate among sites according to a gamma distribution (+G). The best ML tree with bootstrap support values (1000 replicates) is shown. Only bootstrap values higher than 50% are displayed. Carnation latent virus (QJX153999.1) (*Carlavirus latensdianthi*, family *Quinivirinae*) was used as an outgroup, and the tree was rooted with this sequence.

**Figure 7:**
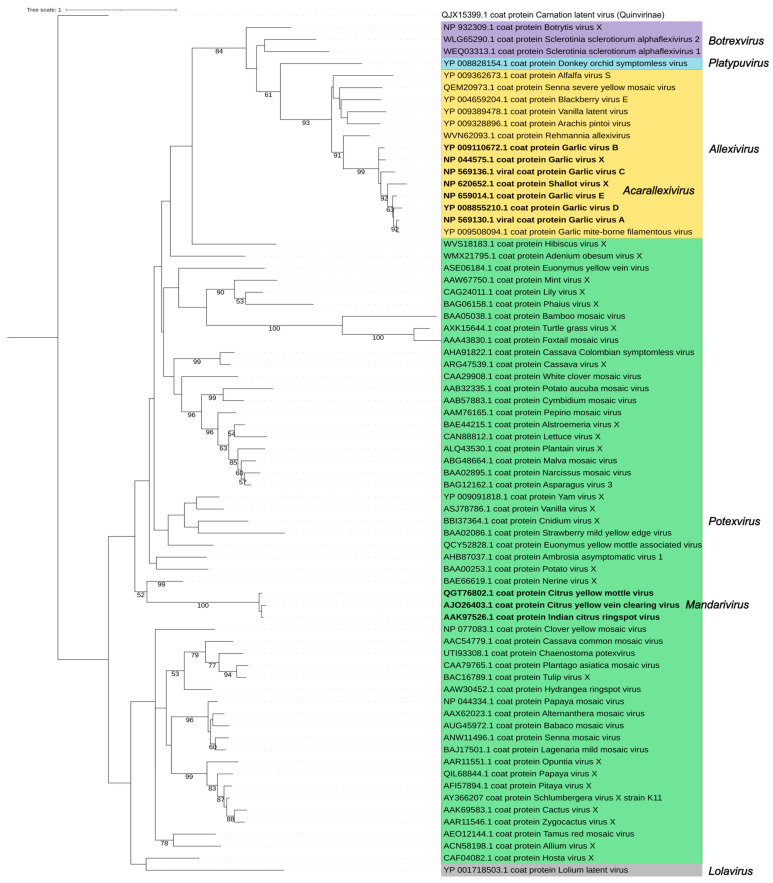
Phylogenetic analysis of the core region of the coat protein (CP) of the family *Alphaflexiviridae*. Phylogenetic tree was constructed excluding the variable N- and C- terminus of the CP sequences (Supplementary Figure S2). The description of the figure is as in Figure 6. Carnation latent virus (QJX153999.1) (*Carlavirus latensdianthi*, family *Quinivirinae*) was used as an outgroup, and the tree was rooted with this sequence.

The replicase and the CP are the major determinants of the *Alphaflexiviridae* taxonomy. A phylogenetic analysis using replicase amino acid sequences of the exemplar viruses for the 72 current *Alphaflexiviridae* species (Figure 5 and Table S2) rendered results congruent with previous analyses [13]. Sequences from viruses in genera *Potexvirus*, *Allexivirus,* and *Botrexvirus* form three distinct lineages; the potex and allexi lineages contain emerging clades for the two subgenera (*Mandarivirus* and *Acarallexivirus*, respectively) defined within each genus. Other small branches with strong significance emerged from the *Potexvirus* lineage and include members infecting hosts from evolutionarily related families; see, for instance, the clade including BaMV, foxtail mosaic virus (*Potexvirus setariae*) and turtle grass virus (*Potexvirus ecsthalassiae*) (Figure 5). The LoLV (genus *Lolavirus*) replicase seems to have evolved from a common allexivirus and potexvirus ancestor replicase. As previously found [13], the replicases of botrexviruses share ancestral sequences with the allexiviruses replicases, to which they are closer in evolutionary terms. The replicases of SsDRV (genus *Sclerodarnavirus*) and DOSV (genus *Platypuvirus*) are more distantly related (Figure 5); in any case, it is appealing that the replicases of viruses infecting fungi share ancestral sequences with those of plant viruses. A similar analysis was carried out for the CP amino acid sequences, providing a tree with less significant branches (Figure 6). Taking into consideration the variability of the *Alphaflexiviridae* CP N-termini, we repeated the analysis with only the core CP sequences excluding variable N-termini, and the results were equivalent (Figure 7). In any case, the CP phylogeny was congruent with that of the replicase, except that allexi-, botrex-, and lolaviruses CPs appear to have evolved from a potexvirus CP ancestor (Figure 6 and Figure 7).

## 4. The Triple Gene Block (TGB) Proteins Are Conserved Among Plant Alphaflexiviruses with a Few Exceptions

The TGB is an evolutionarily conserved gene module fundamental for cell-to-cell and long-distance viral movement through the coordinated action of its three encoded proteins. The TGB is present in plant viruses of different families, including the *Alphaflexiviridae*, in which it is present in all of its members except for genera *Botrexvirus* and *Platypuvirus*. Two potexviruses and most allexiviruses do not have a canonical TGB3 gene annotated in their genomes (Table S2).

The TGB module consists of three nonstructural proteins --TGB1, TGB2, and TGB3--, whose ORFs partially overlap (Figure 1). This distribution may provide a regulatory advantage in terms of timing and protein ratio, which could compensate for the possible loss of fitness due to its reduced ability to tolerate mutations in the overlapping region [44,45]. It is generally assumed that the *Alphaflexiviridae* TGB proteins are expressed from two sgRNAs. TGB1 is expressed from the larger and more abundant sgRNA (sgRNA1), while TGB2 and TGB3 are expressed from sgRNA2, with TGB3 being translated via leaky ribosome scanning through the TGB2 start codon [46].

The TGB1 gene (normally ORF2 downstream of the replicase ORF (Figure 1)), is 600–750 nt long and encodes the largest TGB protein of 24–27 kDa (214–306 aa). The *Alphaflexiviridae* TGB1 is a helicase belonging to a diverged lineage of the viral superfamily 1 of helicases [12]. The helicase domain of TGB1 functions in homologous interactions, cooperatively binding RNA, and has RNA helicase and NTPase activities, dependent on ATP and Mg^2+^ binding [47,48,49,50,51]. A small N-terminal region of ≈25 aa with modulatory functions can be identified upstream of the central helicase domain; three arginine residues within the BaMV N-term domain are important for NTP-binding, ATPase activity, and movement [49,50,52], but only Arg-16 is highly conserved across *Alphaflexiviridae* members (Figure S3). The TGB1 protein plays a crucial role as a viral suppressor of RNA silencing, by limiting the spread of the silencing signal rather than exerting a local effect [53]. However, there are significant differences in the silencing suppression abilities among the different members of the family [54]. The ability of silencing suppression is phenotypically linked to viral movement and, consequently, the host range. This is exemplified by PVX, which can infect *Arabidopsis thaliana* knockout mutants in the ARGONAUTE or DICER-LIKE genes, even though *A. thaliana* is not a natural host. Interestingly, while TGB1-mediated silencing suppression is crucial for viral movement, these functions can be uncoupled and complemented by other proteins such as 12 K [55], P19, or HcPro, in TGB1 point mutants lacking silencing suppression activity. In contrast, TGB1 mutants with movement defects, but fully competent as silencing suppressors, cannot be complemented in trans [56,57,58]. TGB1 undergoes phosphorylation by host kinases, acting as a molecular switch to regulate its diverse functions [59]. The mechanism by which this is regulated remains unclear. However, it seems that the phosphorylated version has the movement function but not the silencing suppression one [60]. This switch between highly diverse functions may explain its localization in multiple subcellular compartments, exhibiting a nucleo-cytoplasmic localization in healthy cells, while it additionally accumulates in plasmodesmata (PD) and VROs during infection [61].

The TGB2 gene, typically 297–405 nt in length, encodes a protein of 11–14 kDa (99–135 aa). Unlike TGB1, TGB2 is an endoplasmic reticulum (ER) membrane-associated protein, characterized by two transmembrane domains separated by a hydrophilic central region. The N-terminal region of the protein is highly conserved across the family, whereas the C-terminal half, including the second transmembrane domain, exhibits significant variability (Figure S4). The predicted topology of this protein, supported by experimental evidence, indicates that both terminal regions are exposed to the cytosolic face of the ER, while the central region remains exposed inside the ER lumen in a U-shaped conformation [12,62], although a W-shaped conformation with an exposed central hydrophilic domain is also plausible [63,64]. TGB2 localizes to the ER, where it induces constrictions to ER tubules [64] and the formation of ER-derived vesicles [61,65] and accumulates in the desmotubule [66]. Some observations suggest that this localization pattern contributes to increasing the PD size exclusion limit (SEL) [67], although other authors attribute the increase in the SEL to TGB1 [68]. TGB2 plays a pivotal role in the organization of VROs by interacting with the C-terminal domain of the RdRp. This interaction is essential for the relocalization of both RdRp and TGB3 to the VROs, and disruption of this interaction significantly reduces replication efficiency and impairs the movement capability of virus [63].

The TGB3 gene (156–378 nt) encodes a protein of 5.8–13 kDa (52–126 aa). This gene likely emerged in the genomic block consisting of TGB1 and TGB2 through overprinting [44]. TGB3 is the smallest and most variable of the TGBs, contains a single transmembrane domain (Figure S5), and it is associated with the ER. According to its predicted topology and experimental evidence with BaMV, the TGB3 N-terminus would be exposed to the lumen, while the C-terminal region would protrude into the cytoplasm [12,69]. Members of the genus *Allexivirus* lack a canonical TGB3, but they contain a TGB3-like protein-coding sequence devoid of an AUG initiator codon. This gene is located in the typical genomic position of TGB3 and similarly translated from a bicistronic mRNA through a leaky scanning mechanism [14]. Two potexviruses infecting cassava lack the TGB3 gene, suggesting that TGB3 may function as an accessory rather than an essential component, potentially playing a role only in specific hosts or tissues [70]. TGB3 expression triggers the unfolded protein response and elicits programmed cell death [71]. Different pathogenicity determinants have been identified in TGB3 [72].

## 5. A Model for Cellular Remodeling Driven by the Alphaflexiviridae Triple Gene Block (TGB) Proteins

**Figure 8:**
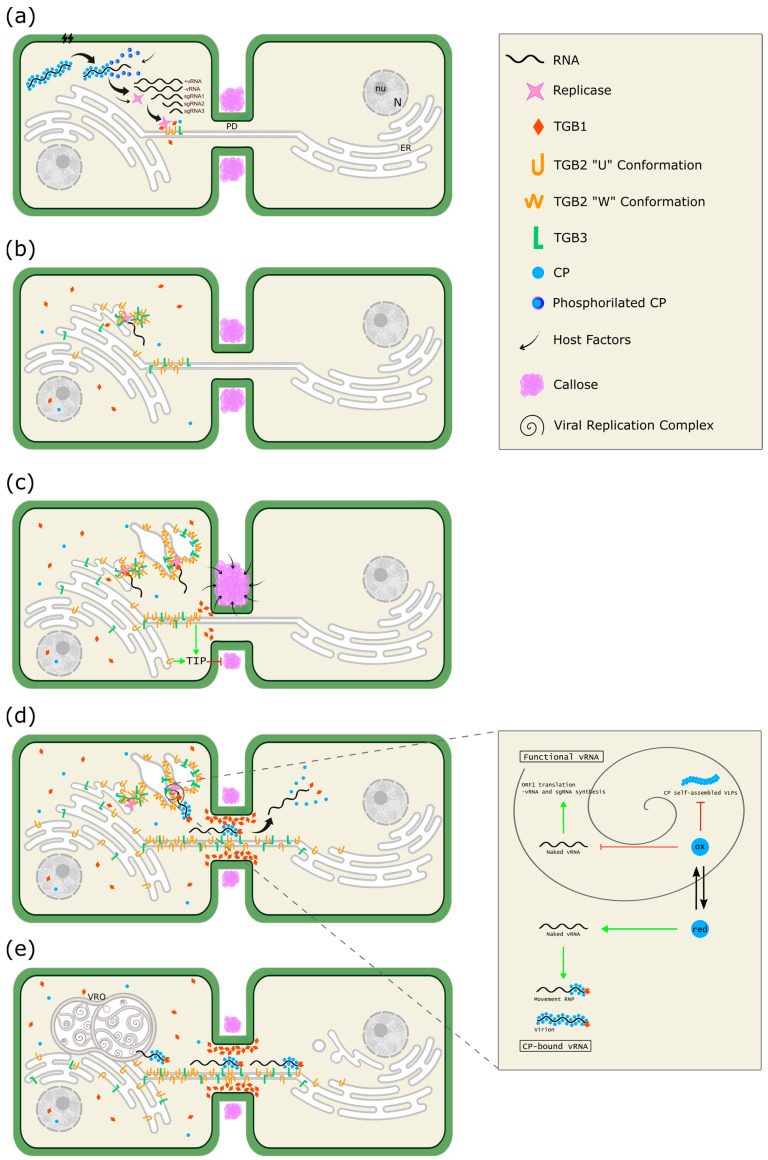
Model of the cellular remodeling coordinated by the triple gene block (TGB) proteins of the family *Alphaflexiviridae*. (**a**) Viral entry and initial replication. The virion penetrates into the host cell through a microlesion and undergoes uncoating via phosphorylation of capsid protein (CP) subunits by host factors. The free viral RNA (+vRNA) recruits the host translation machinery, synthesizing the replicase, which transcribes negative-strand vRNA (−vRNA) and subgenomic RNAs (sgRNA) that serve as templates for replication and translation of the viral proteins, respectively. N: nucleus; nu: nucleolus; ER: endoplasmic reticulum; PD: plasmodesmata. (**b**) Formation of endoplasmic reticulum (ER)-associated granules and TGB2-vesicles. TGB2 and TGB3 move through the ER, forming complexes that recruit the viral replicase and free vRNA, leading to the formation of ER-associated granules that act as early viral replication complexes (VRCs). Additionally, TGB2 induces the formation of trafficking vesicles that recruit TGB3. (**c**) Peripheral body formation. TGB3 contains localization signals directing it to the distal ER, where it undergoes oligomerization and/or conformational changes forming punctate structures. In the distal ER, TGB2 acquires a reticulon-like conformation, remodeling the ER into highly curved membranes and forming peripheral bodies. These structures function as intermediate VRCs, coupling transcription and translation with movement through the plasmodesmata (PD). TGB1 is recruited to PD by TGB3 and TGB2, increasing the size exclusion limit. Through distinct molecular mechanisms, the host perceives the infection and enhances callose deposition to seal the PD and restrict the viral spread. As a countermeasure, TGB2 interacts with TGB2 interacting proteins (TIPs) to activate β-1,3-glucanase, leading to callose degradation and facilitating viral movement through the PD. (**d**) RNP formation and infection of the adjacent cell. Within the VRC, CP may undergo S-oxidation, modifying its redox state and suppressing its affinity for vRNA, freeing it for translation and transcription. In contrast, outside the VRC, the reduced CP exhibits affinity for vRNA, promoting the formation of RNPs. Newly synthesized vRNA begins to encapsidate upon exiting the VRC, with TGB1 subunits binding to the 5′ end, forming the movement ribonucleoprotein (RNP). RNPs associate with TGB2-TGB3, which have colonized the desmotubule. The RNP complex colonizes adjacent cells. TGB1 triggers vRNA uncoating, making it accessible to the host translational machinery. This newly infected cell already possesses a set of viral proteins before the arrival of the vRNA. (**e**) Viral replication pseudo-organelle (VRO) formation. At the final stages of infection, all virus-induced structures concentrate into a VRO through restructuring the endomembrane system. As described by [61], the different VRCs, arranged in a spiral-like formation, associate with reorganized actin filament and contain TGB1 along with TGB2-TGB3 granules. Replication and translation take place in the space between TGB1 and the TGB2-TGB3 granules. This pseudo-organelle harbors large amounts of free vRNA, and nascent virions are located at its periphery.

Based on accumulated evidence mainly obtained through PVX and BaMV research, a model for the cellular remodeling driven by the *Alphaflexiviridae* triple gene block proteins can be proposed (Figure 8). Upon entering the host cell, the virion uncoats to expose the gRNA for translation, a phenomenon likely triggered by CP phosphorylation by host factors [73]. After initial rounds of transcription and translation (Figure 8a), TGB2 and TGB3 begin to associate with ER membranes and move along the ER–actin network through lateral movement by forming complexes with ER luminal-binding protein 4 and calreticulin 3 [74], as well as via TGB2-induced vesicles by a non-conventional mechanism independent of COPII in which TGB3 is subsequently recruited [75]. The TGB2/TGB3-based complexes act as a core from which the ER-associated granules are formed, with TGB2 recruiting the replicase and non-encapsidated vRNA (Figure 8b). These granules may function as the initial viral replication complexes (VRCs) [76]. TGB3 contains signal(s) for its targeting and formation of peripheral bodies, punctuated structures in the cortical ER that contain TGB1, TGB2, and probably other viral and host proteins, arising from the ER-associated granules (Figure 8c). The following events take place in peripheral bodies: (1) TGB2 recruits the replicase and non-encapsidated viral RNA, as in the initial VRCs, and concentrates TGB3 [63]. (2) TGB3 undergoes conformational and/or oligomerization changes, transitioning into punctate structures alongside TGB2. The motility of these punctate structures along the ER is significantly reduced compared to free proteins [77]. (3) The TGB2 exhibits reticulon-like activity, remodeling and stabilizing the cortical ER into highly curved membranes in the close vicinity of plasmodesmata (PD), where peripheral bodies assemble and are stabilized. It has been proposed that TGB2 adopts a “W” conformation, similar to reticulon-like proteins, rather than a “U” conformation, to perform its reticulon-like activity [64]. (4) The activity of the host reticulon-like protein is also hijacked [64,77]. At this stage, PD-anchored viral replication sites can be observed in the cell, and ER-associated granules remain visible (Figure 8c) [66]. As mentioned before, TGB3 is considered the PD targeting factor, playing a key role in directing TGB1 to increase the SEL of PD [68]. Thus, all three TGB proteins colonize the desmotubule. CP is also essential for cell-to-cell movement, but it requires other viral factors to be inserted into PD. Likely, the CP is inserted as a ribonucleoprotein (RNP) composed of newly synthesized genomic RNA that begins to encapsidate as they exit the VRC, with TGB1 bound to its 5′ end (Figure 8d). Outside the VRC, the reduced CP exhibits affinity for vRNA, forming the virion and the RNP. However, within the VRC, the CP may undergo S-oxidation [39], altering its redox state. This modification suppresses its affinity for vRNA or CP oligomerization, thereby making the vRNA available for translation and the synthesis of -vRNA and sgRNA (inset in Figure 8). The RNP is guided through the PD into a new cell by the formation of stable associations between the RNP and TGB2/TGB3-based membrane complexes [69]. Consequently, the new cell would be invaded not only with the vRNA and CP subunits, but also with a set of viral proteins that facilitate the initiation of the infection (Figure 8d). This is exemplified by TGB1, part of the RNP that mediates the RNP/virion disassembly, thereby accelerating the onset of infection, independent of host factors [78]. The recognition of viral proteins by the plant triggers the activation of calcium-dependent kinases, which in turn activate REMORINs, a group of PD-associated proteins. Once activated, REMORINs alter their localization and dynamics within the PD, leading to the deposition of callose, which restricts cell-to-cell movement [79]. As a countermeasure, TGB2 interacts specifically with three TGB2-interating host proteins (TIPs), which are proposed to activate β-1,3-glucanase, leading to callose degradation and facilitating virus movement between cells (Figure 8c) [80].

At later stages of infection, all virus-induced structures accumulate in the perinuclear region, forming a VRO that rivals the size of the cellular nucleus. This VRO is enclosed by reorganized host membranes, a process orchestrated by the coordinated activities of TGB proteins. VROs are highly organized, consisting of distinct VRCs, each containing a core of helically arranged TGB1 aggregates, surrounded by granules of TGB2/TGB3 (Figure 8e). These complexes, and the VRO, remain stable through a network of interactions between RdRp, TGBs, CP, vRNA, and virus-recruited host factors [61,81]. The incorporation of various viral proteins into endosomal vesicles has been documented; however, it remains uncertain whether this process reflects viral protein recycling, plant-mediated degradation, or even a potential mechanism for the virus to colonize non-symplastic connected tissues [75].

## 6. 3′-Terminal Nucleic Acid Binding Proteins Appear to Be Accessory in the Alphaflexivirus Genome

Viruses in subgenera *Acarallexivirus* (genus *Allexivirus*) and *Mandarivirus* (genus *Potexvirus*) have terminal ORFs in their genomes coding for cysteine-rich proteins of around 15 kDa and 23 kDa, respectively (Table S2), which share significant amino acid similarity (51% to 74% for viruses in the same subgenera, and around 30% for viruses in different subgenera), and are annotated as viral nucleic acid binding proteins (NABP). Cysteine-rich proteins are also encoded in the genomes of viruses outside the family *Alphaflexiviridae*; they have a nucleic acid-binding property and play multiple roles in viral infection [82], chiefly, the suppression of RNA silencing and functioning as pathogenicity determinants (e.g., Andika et al. [83]). NABPs of allexiviruses have been studied to some extent, and their activity as RNA silencing suppressors has been demonstrated for some of them [84,85]. In fact, the case of allexiviruses appears to be particularly interesting in terms of both control of the expression and the activity of their NABPs. Allexiviruses encoding NABPs belong to two phylogenetic lineages (groups I and II) [86] (Figure 5, Figure 6 and Figure 7). For allexiviruses in group I, the CP termination codon overlaps with the initiation codon of NABP, while for group II, there is a short sequence inserted between both codons [86]. This short insert acts as a termination upstream ribosome-binding site-like sequence, possibly affecting the recruiting of 18S rRNA to the start codon and therefore translation of the corresponding NABP by a leaky scanning mechanism, even if a sgRNA for this protein has been identified [84]. In addition, NABPs from the different groups seem to have different cellular fates, autophagosome for group I or nuclear accumulation for group II allexiviruses. Provided that CP and NABP function as antagonistic RNA silencing suppressors, nuclear accumulation or autophagosome degradation may contribute to maintaining an adequate CP/NABP balance in the cytoplasm [84].

## 7. Conclusions and Prospects

The family *Alphaflexiviridae* includes a significant number of important plant pathogens, and over the years, research efforts have been dedicated to understanding their biology with a focus on developing strategies for plant disease control. In this context, alphaflexiviruses that infect fungi hold promise, as they can be used, transformed, or leveraged, to generate valuable knowledge for the biocontrol of fungal pathogens. In addition to applied research, viruses in this family have relatively simple genomes, and numerous full-length infectious clones have been generated for a broad variety of them (e.g., Ruíz-Ramón et al. [87]). This makes *Alphaflexiviridae* members manageable experimental models for studying a range of fundamental aspects. The inclusion of plant- and fungus-infecting genera within the family *Alphaflexiviridae* provides a compelling example of trans-kingdom horizontal virus transfer, likely facilitated by the close ecological relationships between plants and their associated fungi, whether pathogenic or symbiotic [88]. Fungal alphaflexiviruses lack canonical movement and/or coat protein genes, in contrast to their plant-infecting counterparts. This pattern suggests that the distinct cellular architecture and mechanisms of intercellular communication in fungi—such as cytoplasmic continuity through hyphal fusion—may have rendered these genes dispensable, driving their loss during adaptation to fungal hosts. A key set of questions, therefore, relates to host determinism, particularly when the hosts belong to different kingdoms. Where are the barriers? Are there transkingdom alphaflexiviruses? What is the evolutionary history of the family, and how has it been shaped by its hosts? Most alphaflexiviruses appear to have narrow host ranges—what are the genetic determinants behind this? Is there a specific role for AlkB in the infection of woody plants? If so, what are the mechanisms, and why are they specifically required for this group of hosts? Similarly, different alphaflexiviruses utilize different transmission modes, providing opportunities to identify experimental systems whose study can expand our understanding of the determinants and mechanisms of transmission.

Over the years, intensive research on a few model alphaflexiviruses has shed light on fundamental cellular processes, including RNA replication and the modular nature of the viral replicase, suppression of RNA silencing, and the functions of TGB proteins and their coordinated actions in transforming the host cell into a virus factory. While we are grasping the general picture, we are still far from fully understanding the many specificities and subtle mechanisms underlying these phenomena. In particular, while we know quite a lot about the functions of viral proteins, these functions rely on a plethora of host factors, which likely perform various roles—either proviral, antiviral, or even both, depending on timing and the cellular context, and are mostly unknown. In this regard, viral proteins can be seen as ideal molecular probes for dissecting fundamental cellular processes, helping researchers to identify and characterize key cellular processes by unveiling their networks of interactions with host factors. Continuing this type of research with well-characterized alphaflexiviruses would undoubtedly yield important results; however, incorporating new alphaflexiviruses into this research may reveal hidden or overlooked aspects with groundbreaking relevance.

Last but not least, the ultrastructure of alphaflexivirus virions has been described in great detail (e.g., Agirrezabala et al. [34]), and several features suggest that alphaflexivirus particles—or virion-like particles—may hold significant biotechnological potential [89]. In conclusion, the family *Alphaflexiviridae* encompasses a diverse array of viruses that are worthy of study from both strategic and fundamental perspectives.

## Data Availability

No new data were created or analyzed in this study. Data sharing is not applicable to this article.

## Supplementary Materials

The following supporting information can be downloaded at: https://www.mdpi.com/article/10.3390/v17050611/s1, Figure S1: 5′ and 3′ untranslated region (UTR) structures of exemplar viruses of each genus and subgenus of the family *Alphaflexiviridae*; Figure S2: Amino acid alignment of coat protein (CP) sequences of members of the family *Alphaflexiviridae*; Figure S3: Amino acid alignment of triple gene block 1 (TGB1) sequences of members of the family *Alphaflexiviridae*; Figure S4: Amino acid alignment of triple gene block 2 (TGB2) sequences of members of the family *Alphaflexiviridae*; Figure S5: Amino acid alignment of triple gene block 3 (TGB3) sequences of members of the family *Alphaflexiviridae*; Table S1: Exemplar viruses of each genus or subgenus within the family Alphaflexiviridae; Table S2: NCBI accession numbers of the viral genomes and proteins of the 72 members of the family *Alphaflexiviridae*.
