# Supplementary material for: Alphaflexiviridae in Focus: Genomic Signatures, Conserved Elements and Viral-Driven Cellular Remodeling
Source: Viruses. 2025 Apr 24;17(5):611. doi: 10.3390/v17050611 (PMC12115993; doi:10.3390/v17050611)
Supplement: Supplementary file 1 [file viruses-17-00611-s001.zip › viruses-3549170-supplementary/Supplementary_files/Figure S1.pptx]

## Slide 1
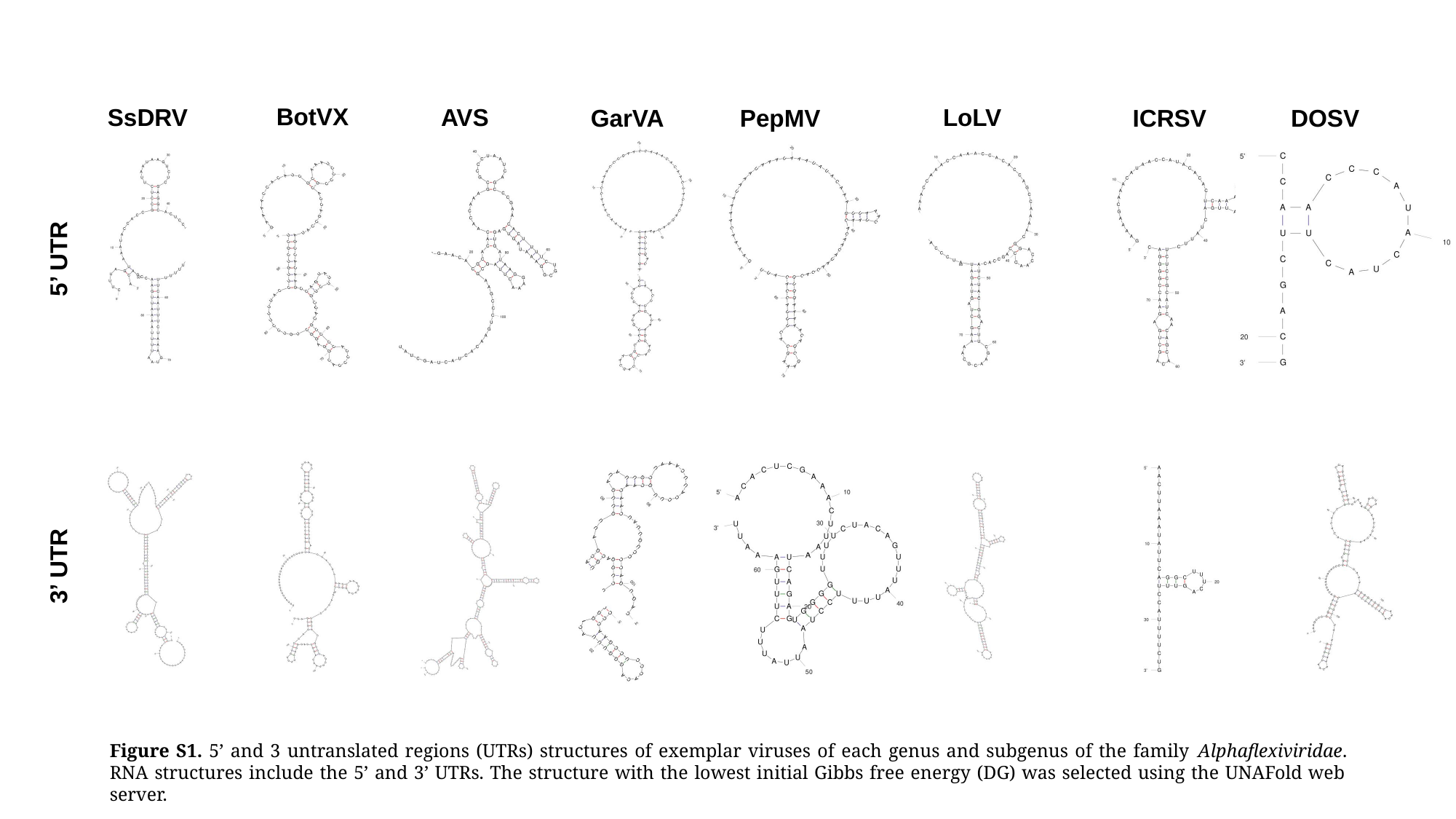

BotVX
SsDRV
AVS
LoLV
GarVA
PepMV
ICRSV
DOSV
5’ UTR
3’ UTR
Figure S1. 5’ and 3 untranslated regions (UTRs) structures of exemplar viruses of each genus and subgenus of the family Alphaflexiviridae. RNA structures include the 5’ and 3’ UTRs. The structure with the lowest initial Gibbs free energy (DG) was selected using the UNAFold web server.
