# Supplementary material for: Alphaflexiviridae in Focus: Genomic Signatures, Conserved Elements and Viral-Driven Cellular Remodeling
Source: Viruses. 2025 Apr 24;17(5):611. doi: 10.3390/v17050611 (PMC12115993; doi:10.3390/v17050611)
Supplement: Supplementary file 1 [file viruses-17-00611-s001.zip › viruses-3549170-supplementary/Supplementary_files/Figure S2.pdf]

CLUSTAL OMEGA (1.2.4) multiple sequence alignment

```
BAA05038.1_coat_protein_[Bamboo_mosaic_virus]      ------------------------------------------------------------------------------------------------------------------------ 0
AAA43830.1_coat_protein_[Foxtail_mosaic_virus]      ------------------------------------------------------------------------------------------------------------------------ 0
AXK15644.1_coat_protein_[Turtle_grass_virus_X]      ------------------------------------------------------------------------------------------------------------------------ 0
WEQ03313.1_coat_protein_[Sclerotinia_sclerotiorum_alphaflexivirus_1]      ------------------------------------------------------------------------------------------------------------------------ 0
NP_932309.1_coat_protein_[Botrytis_virus_X]      ------------------------------------------------------------------------------------------------------------------------ 0
WLG65290.1_coat_protein_[Sclerotinia_sclerotiorum_alphaflexivirus_2]      ------------------------------------------------------------------------------------------------------------------------ 0
YP_008828154.1_coat_protein_[Donkey_orchid_symptomless_virus]      ------------------------------------------------------------------------------------------------------------------------ 0
NP_569136.1_viral_coat_protein_[Garlic_virus_C]      ------------------------------------------------------------------------------------------------------------------------ 0
YP_009110672.1_coat_protein_[Garlic_virus_B]      ------------------------------------------------------------------------------------------------------------------------ 0
NP_044575.1_coat_protein_[Garlic_virus_X]      ------------------------------------------------------------------------------------------------------------------------ 0
NP_620652.1_coat_protein_[Shallot_virus_X]      ------------------------------------------------------------------------------------------------------------------------ 0
YP_008855210.1_coat_protein_[Garlic_virus_D]      ------------------------------------------------------------------------------------------------------------------------ 0
NP_659014.1_coat_protein_[Garlic_virus_E]      ------------------------------------------------------------------------------------------------------------------------ 0
NP_569130.1_viral_coat_protein_[Garlic_virus_A]      ------------------------------------------------------------------------------------------------------------------------ 0
YP_009508094.1_coat_protein_[Garlic_mite-borne_filamentous_virus]      ------------------------------------------------------------------------------------------------------------------------ 0
YP_009362673.1_coat_protein_[Alfalfa_virus_S]      ------------------------------------------------------------------------------------------------------------------------ 0
YP_009389478.1_coat_protein_[Vanilla_latent_virus]      ------------------------------------------------------------------------------------------------------------------------ 0
YP_009328896.1_coat_protein_[Arachis_pintoi_virus]      ------------------------------------------------------------------------------------------------------------------------ 0
QEM20973.1_ORF6_coat_protein_[Senna_severe_yellow_mosaic_virus]      ------------------------------------------------------------------------------------------------------------------------ 0
YP_004659204.1_coat_protein_[Blackberry_virus_E]      ------------------------------------------------------------------------------------------------------------------------ 0
WVN62093.1_coat_protein_[Rehmannia_allexivirus]      MPPLACLGAWLSSHRALANSLNGDARNKRLPQRDTMKFRAHYKLSAATPNSAVPNSRCVKLTARPRTTMAPEPPAGPSIGFCQQARSSIRSSQANSTASLTRSTLSTSTCCNGCKNWITK 120
YP_001718503.1_coat_protein_[Lolium_latent_virus]      ------------------------------------------------------------------------------------------------------------------------ 0
CAF04082.1_coat_protein_[Hosta_virus_X]      ------------------------------------------------------------------------------------------------------------------------ 0
ACN58198.1_coat_protein_[Allium_virus_X]      ------------------------------------------------------------------------------------------------------------------------ 0
AEO12144.1_coat_protein_[Tamus_red_mosaic_virus]      ------------------------------------------------------------------------------------------------------------------------ 0
AUG45972.1_coat_protein_[Babaco_mosaic_virus]      ------------------------------------------------------------------------------------------------------------------------ 0
BAJ17501.1_coat_protein_[Lagenaria_mild_mosaic_virus]      ------------------------------------------------------------------------------------------------------------------------ 0
ANW11496.1_coat_protein_[Senna_mosaic_virus]      ------------------------------------------------------------------------------------------------------------------------ 0
AAX62023.1_coat_protein_[Alternanthera_mosaic_virus]      ------------------------------------------------------------------------------------------------------------------------ 0
NP_044334.1_capsid_protein_[Papaya_mosaic_virus]      ------------------------------------------------------------------------------------------------------------------------ 0
NP_077083.1_coat_protein_[Clover_yellow_mosaic_virus]      ------------------------------------------------------------------------------------------------------------------------ 0
AAR11551.1_coat_protein_[Opuntia_virus_X]      ------------------------------------------------------------------------------------------------------------------------ 0
QIL68844.1_coat_protein_[Papaya_virus_X]      ------------------------------------------------------------------------------------------------------------------------ 0
AFI57894.1_coat_protein_[Pitaya_virus_X]      ------------------------------------------------------------------------------------------------------------------------ 0
AY366207_coat_protein_[Schlumbergera_virus_X]      ------------------------------------------------------------------------------------------------------------------------ 0
AAK69583.1_coat_protein_[Cactus_virus_X]      ------------------------------------------------------------------------------------------------------------------------ 0
AAR11546.1_coat_protein_[Zygocactus_virus_X]      ------------------------------------------------------------------------------------------------------------------------ 0
AAW30452.1_coat_protein_[Hydrangea_ringspot_virus]      ------------------------------------------------------------------------------------------------------------------------ 0
AAC54779.1_coat_protein_[Cassava_common_mosaic_virus]      ------------------------------------------------------------------------------------------------------------------------ 0
UTI93308.1_coat_protein_[Chaenostoma_potexvirus]      ------------------------------------------------------------------------------------------------------------------------ 0
CAA79765.1_coat_protein_[Plantago_asiatica_mosaic_virus]      ------------------------------------------------------------------------------------------------------------------------ 0
BAC16789.1_coat_protein_[Tulip_virus_X]      ------------------------------------------------------------------------------------------------------------------------ 0
QGT76802.1_coat_protein_[Citrus_yellow_mottle_virus]      ------------------------------------------------------------------------------------------------------------------------ 0
AJO26403.1_coat_protein_[Citrus_yellow_vein_clearing_virus]      ------------------------------------------------------------------------------------------------------------------------ 0
AAK97526.1_coat_protein_[Indian_citrus_ringspot_virus]      ------------------------------------------------------------------------------------------------------------------------ 0
WVS18183.1_coat_protein_[Hibiscus_virus_X]      ------------------------------------------------------------------------------------------------------------------------ 0
BAG06158.1_coat_protein_[Phaius_virus_X]      ------------------------------------------------------------------------------------------------------------------------ 0
CAG24011.1_coat_protein_[Lily_virus_X]      ------------------------------------------------------------------------------------------------------------------------ 0
AAW67750.1_coat_protein_[Mint_virus_X]      ------------------------------------------------------------------------------------------------------------------------ 0
BAA02086.1_coat_protein_[Strawberry_mild_yellow_edge_virus]      ------------------------------------------------------------------------------------------------------------------------ 0
ASE06184.1_coat_protein_[Euonymus_yellow_vein_virus]      ------------------------------------------------------------------------------------------------------------------------ 0
CAA29908.1_coat_protein_[White_clover_mosaic_virus]      ------------------------------------------------------------------------------------------------------------------------ 0
CAN88812.1_coat_protein_[Lettuce_virus_X]      ------------------------------------------------------------------------------------------------------------------------ 0
AAM76165.1_coat_protein_[Pepino_mosaic_virus]      ------------------------------------------------------------------------------------------------------------------------ 0
ALQ43530.1_coat_protein_[Plantain_virus_X]      ------------------------------------------------------------------------------------------------------------------------ 0
BAE44215.1_coat_protein_[Alstroemeria_virus_X]      ------------------------------------------------------------------------------------------------------------------------ 0
BAA02895.1_coat_protein_[Narcissus_mosaic_virus]      ------------------------------------------------------------------------------------------------------------------------ 0
BAG12162.1_coat_protein_[Asparagus_virus_3]      ------------------------------------------------------------------------------------------------------------------------ 0
ABG48664.1_coat_protein_[Malva_mosaic_virus]      ------------------------------------------------------------------------------------------------------------------------ 0
AAB57883.1_coat_protein_[Cymbidium_mosaic_virus]      ------------------------------------------------------------------------------------------------------------------------ 0
AAB32335.1_coat_protein_[Potato_aucuba_mosaic_virus]      ------------------------------------------------------------------------------------------------------------------------ 0
WMX21795.1_coat_protein_[Adenium_obesum_virus_X]      ------------------------------------------------------------------------------------------------------------------------ 0
AHA91822.1_coat_protein_[Cassava_Colombian_symptomless_virus]      ------------------------------------------------------------------------------------------------------------------------ 0
ARG47539.1_coat_protein_[Cassava_virus_X]      ------------------------------------------------------------------------------------------------------------------------ 0
BAA00253.1_coat_protein_[Potato_virus_X]      ------------------------------------------------------------------------------------------------------------------------ 0
QCY52828.1_coat_protein_[Euonymus_yellow_mottle_associated_virus]      ------------------------------------------------------------------------------------------------------------------------ 0
AHB87037.1_coat_protein_[Ambrosia_asymptomatic_virus_1]      ------------------------------------------------------------------------------------------------------------------------ 0
BBI37364.1_coat_protein_[Cnidium_virus_X]      ------------------------------------------------------------------------------------------------------------------------ 0
BAE66619.1_coat_protein_[Nerine_virus_X]      ------------------------------------------------------------------------------------------------------------------------ 0
ASJ78786.1_coat_protein_[Vanilla_virus_X]      ------------------------------------------------------------------------------------------------------------------------ 0
YP_009091818.1_coat_protein_[Yam_virus_X]      ------------------------------------------------------------------------------------------------------------------------ 0
```

BAA05038.1_coat_protein_[Bamboo_mosaic_virus]	-- 0
AAA43830.1_coat_protein_[Foxtail_mosaic_virus]	-- 0
AXK15644.1_coat_protein_[Turtle_grass_virus_X]	-- 0
WEQ03313.1_coat_protein_[Sclerotinia_sclerotiorum_alphaflexivirus_1]	-- 0
NP_932309.1_coat_protein_[Botrytis_virus_X]	------------MDPNLDQDTLPTHEEIDNDVDSAEEEPPEPP----------LLPDDID-DDDSHGSRTRRQV--KPPPEL------------------LRAVGACLISGHYDGGNYFR 77
WLG65290.1_coat_protein_[Sclerotinia_sclerotiorum_alphaflexivirus_2]	---M 1
YP_008828154.1_coat_protein_[Donkey_orchid_symptomless_virus]	-- 0
NP_569136.1_viral_coat_protein_[Garlic_virus_C]	-- 0
YP_009110672.1_coat_protein_[Garlic_virus_B]	-- 0
NP_044575.1_coat_protein_[Garlic_virus_X]	-- 0
NP_620652.1_coat_protein_[Shallot_virus_X]	-- 0
YP_008855210.1_coat_protein_[Garlic_virus_D]	-- 0
NP_659014.1_coat_protein_[Garlic_virus_E]	-- 0
NP_569130.1_viral_coat_protein_[Garlic_virus_A]	-- 0
YP_009508094.1_coat_protein_[Garlic_mite-borne_filamentous_virus]	-- 0
YP_009362673.1_coat_protein_[Alfalfa_virus_S]	-- 0
YP_009389478.1_coat_protein_[Vanilla_latent_virus]	-- 0
YP_009328896.1_coat_protein_[Arachis_pintoi_virus]	-- 0
QEM20973.1_ORF6_coat_protein_[Senna_severe_yellow_mosaic_virus]	-- 0
YP_004659204.1_coat_protein_[Blackberry_virus_E]	-- 0
WVN62093.1_coat_protein_[Rehmannia_allexivirus]	RQISAGNSRNRPETPAITMSPL--GRNWPRLLRTSPAWPPTKIARPSQLRSTGWLPSSHKQPRASHLVQTRHRLAPRRPPSQLCPHTCHTIQRRLAALAPFSSVKTCAPAYHLIGSENPR 238
YP_001718503.1_coat_protein_[Lolium_latent_virus]	-- 0
CAF04082.1_coat_protein_[Hosta_virus_X]	-- 0
ACN58198.1_coat_protein_[Allium_virus_X]	-- 0
AEO12144.1_coat_protein_[Tamus_red_mosaic_virus]	-- 0
AUG45972.1_coat_protein_[Babaco_mosaic_virus]	-- 0
BAJ17501.1_coat_protein_[Lagenaria_mild_mosaic_virus]	-- 0
ANW11496.1_coat_protein_[Senna_mosaic_virus]	-- 0
AAX62023.1_coat_protein_[Alternanthera_mosaic_virus]	-- 0
NP_044334.1_capsid_protein_[Papaya_mosaic_virus]	-- 0
NP_077083.1_coat_protein_[Clover_yellow_mosaic_virus]	-- 0
AAR11551.1_coat_protein_[Opuntia_virus_X]	-- 0
QIL68844.1_coat_protein_[Papaya_virus_X]	-- 0
AFI57894.1_coat_protein_[Pitaya_virus_X]	-- 0
AY366207_coat_protein_[Schlumbergera_virus_X]	-- 0
AAK69583.1_coat_protein_[Cactus_virus_X]	-- 0
AAR11546.1_coat_protein_[Zygocactus_virus_X]	-- 0
AAW30452.1_coat_protein_[Hydrangea_ringspot_virus]	-- 0
AAC54779.1_coat_protein_[Cassava_common_mosaic_virus]	-- 0
UTI93308.1_coat_protein_[Chaenostoma_potexvirus]	-- 0
CAA79765.1_coat_protein_[Plantago_asiatica_mosaic_virus]	-- 0
BAC16789.1_coat_protein_[Tulip_virus_X]	-- 0
QGT76802.1_coat_protein_[Citrus_yellow_mottle_virus]	---MSLDY-- 5
AJO26403.1_coat_protein_[Citrus_yellow_vein_clearing_virus]	---MSFDY-- 5
AAK97526.1_coat_protein_[Indian_citrus_ringspot_virus]	---MSFDY-- 5
WVS18183.1_coat_protein_[Hibiscus_virus_X]	-- 0
BAG06158.1_coat_protein_[Phaius_virus_X]	-- 0
CAG24011.1_coat_protein_[Lily_virus_X]	-- 0
AAW67750.1_coat_protein_[Mint_virus_X]	-- 0
BAA02086.1_coat_protein_[Strawberry_mild_yellow_edge_virus]	-- 0
ASE06184.1_coat_protein_[Euonymus_yellow_vein_virus]	---MSASKAE 7
CAA29908.1_coat_protein_[White_clover_mosaic_virus]	-- 0
CAN88812.1_coat_protein_[Lettuce_virus_X]	-- 0
AAM76165.1_coat_protein_[Pepino_mosaic_virus]	-- 0
ALQ43530.1_coat_protein_[Plantain_virus_X]	-- 0
BAE44215.1_coat_protein_[Alstroemeria_virus_X]	-- 0
BAA02895.1_coat_protein_[Narcissus_mosaic_virus]	-- 0
BAG12162.1_coat_protein_[Asparagus_virus_3]	-- 0
ABG48664.1_coat_protein_[Malva_mosaic_virus]	-- 0
AAB57883.1_coat_protein_[Cymbidium_mosaic_virus]	-- 0
AAB32335.1_coat_protein_[Potato_aucuba_mosaic_virus]	-- 0
WMX21795.1_coat_protein_[Adenium_obesum_virus_X]	-- 0
AHA91822.1_coat_protein_[Cassava_Colombian_symptomless_virus]	-- 0
ARG47539.1_coat_protein_[Cassava_virus_X]	-- 0
BAA00253.1_coat_protein_[Potato_virus_X]	-- 0
QCY52828.1_coat_protein_[Euonymus_yellow_mottle_associated_virus]	-- 0
AHB87037.1_coat_protein_[Ambrosia_asymptomatic_virus_1]	-- 0
BBI37364.1_coat_protein_[Cnidium_virus_X]	-- 0
BAE66619.1_coat_protein_[Nerine_virus_X]	-- 0
ASJ78786.1_coat_protein_[Vanilla_virus_X]	-- 0
YP_009091818.1_coat_protein_[Yam_virus_X]	-- 0

BAA05038.1_coat_protein_[Bamboo_mosaic_virus]	---MS------GT-------------GT	6
AAA43830.1_coat_protein_[Foxtail_mosaic_virus]	--	0
AXK15644.1_coat_protein_[Turtle_grass_virus_X]	---MS------QA-------------PT	6
WEQ03313.1_coat_protein_[Sclerotinia_sclerotiorum_alphaflexivirus_1]	---MSD-ESKPDSV---EIQ--L-----------------LDD---	17
NP_932309.1_coat_protein_[Botrytis_virus_X]	WQQS-IAALYAKAGYAGDIRFHQAAIQEYALDPVL----------PA--PR------VSYDLLVAHAGLRYQALLN-----EQLR----------------TGKTP---PADEALKDA--	152
WLG65290.1_coat_protein_[Sclerotinia_sclerotiorum_alphaflexivirus_2]	TLADEVI-------------------------------TIQRTNTPAAWSQLSSKL------QSLAAAQNWHFTAD------EYD--------Q--A----ARDYQ-ANPTALA------	57
YP_008828154.1_coat_protein_[Donkey_orchid_symptomless_virus]	--	0
NP_569136.1_viral_coat_protein_[Garlic_virus_C]	---MSG-DSLSDDL---VNA-------AMTDPS-------------	19
YP_009110672.1_coat_protein_[Garlic_virus_B]	--MG-D-----------R------SQGTNP--------------	10
NP_044575.1_coat_protein_[Garlic_virus_X]	--MG-D-----------R------NQGINP--------------	10
NP_620652.1_coat_protein_[Shallot_virus_X]	---MNE-EDLNRLN---A---------SGDL---------------	15
YP_008855210.1_coat_protein_[Garlic_virus_D]	---MNEQG--------------	5
NP_659014.1_coat_protein_[Garlic_virus_E]	----MTA--------TYCCRRTLKPLISYNIA------------QTIVSPFYIRDAKTSSTRLGLMVCVKLAQRLNLMNG-DEFSDQD---L---------MNDPT--------------	69
NP_569130.1_viral_coat_protein_[Garlic_virus_A]	---MNNPV--------------	5
YP_009508094.1_coat_protein_[Garlic_mite-borne_filamentous_virus]	---MNDPV--------------	5
YP_009362673.1_coat_protein_[Alfalfa_virus_S]	---------------------------------MSQHRTDSQSCLEMPFSSYTKSAQTLLTSLRTAMCVKFEVMFE-------------------------IIKMPESNPRVI-------	55
YP_009389478.1_coat_protein_[Vanilla_latent_virus]	--	0
YP_009328896.1_coat_protein_[Arachis_pintoi_virus]	---MA-----------------	2
QEM20973.1_ORF6_coat_protein_[Senna_severe_yellow_mosaic_virus]	---MTGTPPISPRAXAGPSTPT	19
YP_004659204.1_coat_protein_[Blackberry_virus_E]	---MPDTPANPPANQAPNPVNA	19
WVN62093.1_coat_protein_[Rehmannia_allexivirus]	TLADQSASLSKAAPLTPFALFAFSPLVFYNSHEQSPHPTPSRTCPQTRWPYYTHIVPTSYTNSRACAELSWAIVRIMSDA-ERQAAEA---AAK--ARNDAPPALPVTPPVVPTGDNRSV	352
YP_001718503.1_coat_protein_[Lolium_latent_virus]	---MSESKAETPSKSA-----EKGVASLSTS--AP--------PSSTTPT	32
CAF04082.1_coat_protein_[Hosta_virus_X]	--	0
ACN58198.1_coat_protein_[Allium_virus_X]	---MTDKT--------------------	5
AEO12144.1_coat_protein_[Tamus_red_mosaic_virus]	--MA--------------------	2
AUG45972.1_coat_protein_[Babaco_mosaic_virus]	--	0
BAJ17501.1_coat_protein_[Lagenaria_mild_mosaic_virus]	--	0
ANW11496.1_coat_protein_[Senna_mosaic_virus]	--	0
AAX62023.1_coat_protein_[Alternanthera_mosaic_virus]	--	0
NP_044334.1_capsid_protein_[Papaya_mosaic_virus]	--	0
NP_077083.1_coat_protein_[Clover_yellow_mosaic_virus]	--	0
AAR11551.1_coat_protein_[Opuntia_virus_X]	--MASTPQT-------------------	7
QIL68844.1_coat_protein_[Papaya_virus_X]	---M------------------------	1
AFI57894.1_coat_protein_[Pitaya_virus_X]	---M------------------------	1
AY366207_coat_protein_[Schlumbergera_virus_X]	---M------------------------	1
AAK69583.1_coat_protein_[Cactus_virus_X]	---M------------------------	1
AAR11546.1_coat_protein_[Zygocactus_virus_X]	--MS------------------------	2
AAW30452.1_coat_protein_[Hydrangea_ringspot_virus]	---MTTTT--------------------	5
AAC54779.1_coat_protein_[Cassava_common_mosaic_virus]	---MATPT--------------------	5
UTI93308.1_coat_protein_[Chaenostoma_potexvirus]	--	0
CAA79765.1_coat_protein_[Plantago_asiatica_mosaic_virus]	--	0
BAC16789.1_coat_protein_[Tulip_virus_X]	--	0
QGT76802.1_coat_protein_[Citrus_yellow_mottle_virus]	----------------------------------Q-H--------PNFRN-YP--YPHYCEYDKHQYCDHHPELRKPKHP--Q-----PSDPPALMSNTQAKGKEPLETTPVAPSAQGN-	71
AJO26403.1_coat_protein_[Citrus_yellow_vein_clearing_virus]	----------------------------------T-H--------PLYRS-YP--FPHYCEFDRHQLCDHHPVLKPP-TH--K-----PSAPNSLMSTNDNKGKEPLHPTPSGPNDTTPK	71
AAK97526.1_coat_protein_[Indian_citrus_ringspot_virus]	----------------------------------T-D--------PTFRN-YP--FPHYCDFDRHQHCDHDLRTNPPPTE--P-----PSRKSKLMSTSENKGKQPLHPPPTEGFPKPP-	71
WVS18183.1_coat_protein_[Hibiscus_virus_X]	---MSNTTA--APSDAEIEAARK-----ALADAPENVPQLRQER---QRELDALIARRNAPPPAPPRVGPSPVVPS	63
BAG06158.1_coat_protein_[Phaius_virus_X]	--	0
CAG24011.1_coat_protein_[Lily_virus_X]	--	0
AAW67750.1_coat_protein_[Mint_virus_X]	---MTNPTD----------D-AAA---A	11
BAA02086.1_coat_protein_[Strawberry_mild_yellow_edge_virus]	---MGD-----------------------QP--RPPVP-PAP----	13
ASE06184.1_coat_protein_[Euonymus_yellow_vein_virus]	W---------------------------YAGLEQG-D----RTRIAAFRDRTK--GSTQADYDR--------EANAFVATISEGARAG---------FNTH--YGATHPPRD-PNA---R	70
CAA29908.1_coat_protein_[White_clover_mosaic_virus]	--	0
CAN88812.1_coat_protein_[Lettuce_virus_X]	---MGT-PQAVN-----------------TPVNNPPR---------	16
AAM76165.1_coat_protein_[Pepino_mosaic_virus]	--MPDTTPVA-ATS----	11
ALQ43530.1_coat_protein_[Plantain_virus_X]	---MTD-PSKNVSG---------------GPTNPPPKV--AS----	21
BAE44215.1_coat_protein_[Alstroemeria_virus_X]	---M-AGE----	4
BAA02895.1_coat_protein_[Narcissus_mosaic_virus]	---M----	1
BAG12162.1_coat_protein_[Asparagus_virus_3]	---M-ANT----	4
ABG48664.1_coat_protein_[Malva_mosaic_virus]	---MSNSG---------------SAAAAPSQP-SAA----	17
AAB57883.1_coat_protein_[Cymbidium_mosaic_virus]	--	0
AAB32335.1_coat_protein_[Potato_aucuba_mosaic_virus]	---MVD-SKKTETP---------------QVIDASKKT-ESS---K	23
WMX21795.1_coat_protein_[Adenium_obesum_virus_X]	---MNPVPSS-SQGSESQHMVAQSLRNLPT--------------YS---A	29
AHA91822.1_coat_protein_[Cassava_Colombian_symptomless_virus]	--ME---E	3
ARG47539.1_coat_protein_[Cassava_virus_X]	--	0
BAA00253.1_coat_protein_[Potato_virus_X]	---MSAP---------------AS---T	7
QCY52828.1_coat_protein_[Euonymus_yellow_mottle_associated_virus]	---MSTLADLQA--------RLAAATND-EQRAEAQ----RALDEFNDEGANP---PAIP-PNA---E	45
AHB87037.1_coat_protein_[Ambrosia_asymptomatic_virus_1]	---MSTREELER--------QLAEAETD-EQKTELQ----SKIDDLPEDQGQGDNGGDGA-SG----G	47
BBI37364.1_coat_protein_[Cnidium_virus_X]	---MVLT-------AEETAA-SS----T	13
BAE66619.1_coat_protein_[Nerine_virus_X]	--	0
ASJ78786.1_coat_protein_[Vanilla_virus_X]	--	0
YP_009091818.1_coat_protein_[Yam_virus_X]	--	0

```
BAA05038.1_coat_protein_[Bamboo_mosaic_virus]                          GTGRGTGT---GVGGT--GGTGG-----------TG--------------GGGTGRGQQAAPQ--PWETKFT--KDDLAAIEPKPASANVPNTKQWISIQAGL-IKAGATDANFMKVLLG 91
AAA43830.1_coat_protein_[Foxtail_mosaic_virus]                         ---------------------MA-----------TQ-------------------NADVTDAT--DYKKPPA--ETEQKALTIQPRSNKAPSDEELVRIINAA-QKRGLTPAAFVQAAIV 64
AXK15644.1_coat_protein_[Turtle_grass_virus_X]                         PTRAQT-----------QSSAG-----------TG--------------S----SQVHTDVA--ALKTKLD--ASAQEAITLQPRSSKAPTDEELTRIATLA-SDRGLPTASFCEAAIL 80
WEQ03313.1_coat_protein_[Sclerotinia_sclerotiorum_alphaflexivirus_1]   ---AQGPK----VSSH---SSITADLHDQLKTALQH-----TKKTPLI-----AAPRRIVTMS--TGELTL---TALKELKHPPYSSNSVATQAQVHTILTNL-AAY--DAKIAAPQLLE 109
NP_932309.1_coat_protein_[Botrytis_virus_X]                            VRKAAQAA----YDNA---VKTG-DYTPLIDIAFKG-----VDINKHASDVAQLAK--MSVTM--DGTHIKF--TAGEMPKDKVITSNSMASPNTVMNILNLI-TTS--ANVTAVTCGIE 250
WLG65290.1_coat_protein_[Sclerotinia_sclerotiorum_alphaflexivirus_2]   --DTANPT----QGEK---NSVA-SAVHNLLL-AAH-----NA-R-------------ASRQV--P-AVMSFSLTDAQINGAHTVIANSVATPTQITSILAEK-V-L--TVSVTPTALLE 140
YP_008828154.1_coat_protein_[Donkey_orchid_symptomless_virus]          -------------------------------------------------------MSIPKPS--D-ALFPDA-PDSFLILDETISSNSIATLAEVQKIHTAL-G------ENAPKISLA 53
NP_569136.1_viral_coat_protein_[Garlic_virus_C]                        TQGLNQAT----SAQG---SR------PG-LV-SGP-----GSQTPSKNPR-PSSTQGGPTVT--N-ELLPS--ESELEAVANDVTSNSVATQSTIREILDLL-RAR--KPSATPKDLFS 110
YP_009110672.1_coat_protein_[Garlic_virus_B]                           AQGLNAQV----QGDG---------------T-SGQ-----SQRNPNRNPP-PR-PQADLNAQ--D-NIMPS--ESDLAAIAGDVTSNSVATKETVKEILSNL-QAR--RINATPKDLFS 95
NP_044575.1_coat_protein_[Garlic_virus_X]                              NQGLNAQV----QGDG---------------A-SNQ-----NQ-RPNRNPP-PR-PLADSNAQ--N-SIMPS--ENDLAAIASDVTSNSVATQETIREILSTL-QAS--GPAATPKDLFS 94
NP_620652.1_coat_protein_[Shallot_virus_X]                             NGANNQRI----PAGP---SGVN-QPIPSVTA-GGQ-----NQFRPSGGLGNQGSRPTESSNQ--D-ELLPT--EAEIEAITSDVESNSVAPKATIREILDTL-QAK--RQNATPKDLFS 113
YP_008855210.1_coat_protein_[Garlic_virus_D]                           NT-STQSP----RGTV---NPPG-PPQPNVSA-FGQ-----GQTRPSGSRDPPNAN-PITNEQ--D-ELMPA--VTEFENLANDVESNSLASRATIRDILDML-QAT--RQGATPKDLFS 101
NP_659014.1_coat_protein_[Garlic_virus_E]                              NAGTRPPP----QSSG---SQSE-QARPNVTA-FGQ-----NQLRPTGSREPTNGSNMGTNEQ--N-DLMPA--VNDFEALTADVESNSVASRTTVREILDML-QAK--RQGATPKDLFS 167
NP_569130.1_viral_coat_protein_[Garlic_virus_A]                        DPSTSQQM----SGTT---GQVS-QPRPNVNP-FGQ-----SQTRPLGNRDPQNNS-NASDDS--D-ALMPA--VTDFETLTKDVESNSVASRLIIRDILDML-QAK--RQGATPKDLFS 102
YP_009508094.1_coat_protein_[Garlic_mite-borne_filamentous_virus]      DPSTSQQM----SGST---SQVS-QPRPNVNP-FGQ-----TQLRSLGNRDPQNNSSNASNES--D-ELMPA--LAEFETLTKDVESNSVASRTIIREILDLL-QAK--RQGATPKDLLS 103
YP_009362673.1_coat_protein_[Alfalfa_virus_S]                          --PPNPNE----TN-T---S---------VP--PQG-----N-------QTRPPAPDNNTAGD--D-LLMPS--LEEIQLITREIDSNTVATKQTVTEILDLL-KAD--HREADRSDLFQ 134
YP_009389478.1_coat_protein_[Vanilla_latent_virus]                     -------M----DTST---TNIG-TGS----S-SQK-----NPLT---KDQPPPPTAQDAGET--D-SLMPT--LQELEAIMPTIAANKVATRPMITAILTQV-RGQ--HASATATDLVE 84
YP_009328896.1_coat_protein_[Arachis_pintoi_virus]                     --SNAPRP----EDSA---RPIS-GAVPNTPP-NNQ-----SR------------PETSAPTR--D-PLLPS--REEMQLVTRDVQANKIASRQQVEAILQQL-------RGGNVNDLMS 82
QEM20973.1_ORF6_coat_protein_[Senna_severe_yellow_mosaic_virus]        TSPTATPP----AAAA---NQAG-RVAPQVPA-NVQ-----SAIR---APEPGPATANQEQAM--D-DILPS--PQELETIGADISANKIATPQVIRTILANA-QRV--QPSATALDLVD 114
YP_004659204.1_coat_protein_[Blackberry_virus_E]                       PNPPPPPP----RNPA---S--------------------------------------GGSSA--N-PLIPA--DDELATLGTTVQANKVATQDAVSAVLAEL-QQG--FPSATAIDLVS 86
WVN62093.1_coat_protein_[Rehmannia_allexivirus]                        PVPPTPPP----TGSD---SSAG-QG----P----G-----SNVR--------PPQTPTNVST--N-PLMPT--FEELGDVAT--FSNSIATQQTVRDVLAMA-QAQ--YPGVTAKDLIT 433
YP_001718503.1_coat_protein_[Lolium_latent_virus]                      AQAKQTPP---PVA--TTARPMASRLPRTIAAEGGGTEKKQSHLAEDRI-AQYLPKQDAVDHSNLAALLQPF--TEASYEREFDVKVNGIASKAELTAVAETWASRLNVPKENSAVLAQE 144
CAF04082.1_coat_protein_[Hosta_virus_X]                                ------------------------------------M--------------ASDAPTPPAAPSP--VTFTAPT--QEQLTSLALPIISTRLPSPDVLNQISVKW-QELGVPTASISSTAIA 66
ACN58198.1_coat_protein_[Allium_virus_X]                               ------------------------------------A--------------ATNVLSKAVNSNA--IPFTTPK--PEELSAMTFTLNNRSLPTPGELIAIAEQW-KTLGVPGEAVTTHALQ 71
AEO12144.1_coat_protein_[Tamus_red_mosaic_virus]                       ------------------------------------S--------------PPSPANQPKVESN--NPFKAPT--LDRLKLMTFDVNSTLLPSTEEFKLISEDW-AKLGISGDSLTENAMK 68
AUG45972.1_coat_protein_[Babaco_mosaic_virus]                          ----------------------------------------------------MSGKSESSNTGN--SPFPNLT--KETMASFNFKPSSNLLPSEEELKIISTLL-VAAKIPNASTTIVAMD 64
BAJ17501.1_coat_protein_[Lagenaria_mild_mosaic_virus]                  -------------------------------------------------------------MSA--TPFPAVT--PELLAELKLGPTSNLLPTQDQLKIIADLI-VAAKVPAASTTKVALE 55
ANW11496.1_coat_protein_[Senna_mosaic_virus]                           ----------------------------------------------------------------MAFTAPT--EEMMATFKPIPASNLLPTQEQLKTVTDLF-RAAKVPDASMTSVALE 52
AAX62023.1_coat_protein_[Alternanthera_mosaic_virus]                   ---------------------------------------------------------------MS--TPFPQIT--QEQMNAFTPHATSNLLPSSEQLTTIASLL-VAAKVPAASTTTIALE 54
NP_044334.1_capsid_protein_[Papaya_mosaic_virus]                       ------------------------------------------------------MSKSSMSTPN--IAFPAIT--QEQMSSIKVDPTSNLLPSQEQLKSVSTLM-VAAKVPAASVTTVALE 62
NP_077083.1_coat_protein_[Clover_yellow_mosaic_virus]                  ----------------------------------------------------------MTDTKK--TLFSAPT--DEQLDTLTLTIESNLVPSISELEAIAKDW-KTLGLQEADFTANAIK 58
AAR11551.1_coat_protein_[Opuntia_virus_X]                              ----SSTP--------------------------VQ--------------SS-AARNPINNHG--GPFDSLT--TDQINSLSLTVTSSLLPSTQALQAIANAF-TSLGASQAQVTNLALE 77
QIL68844.1_coat_protein_[Papaya_virus_X]                               ----STPP--------------------------AD--------------KK-ATSSTTSNAP--LLFPTLS--NVDLSTLQFTTTSSLLPSDDDLSLIFQEF-NKLGATSTNATQVALE 71
AFI57894.1_coat_protein_[Pitaya_virus_X]                               ----ATQT--------------------------AQ--------------SSSSVGARTNPTS--GPFQTLS--MSQLTALPLSVTSSLLPSSDDLTAIATAL-QALGATPANLTLVSLE 72
AY366207_coat_protein_[Schlumbergera_virus_X]                          ----STTP--------------------------SS--------------ASNSIGSRVPTAQ--GPFASLT--SSQLNVLPLSVTSSLLPSPEDLVTISQAF-ITLGAAQTNLTQVSLE 72
AAK69583.1_coat_protein_[Cactus_virus_X]                               ----STTG--------------------------VQ--------------SSQSSGPRSTPQS--GPFQTLS--SSQLAALSLGVTSSLLPSPAELVSISQAL-TTLGASATNLTPLSLE 72
AAR11546.1_coat_protein_[Zygocactus_virus_X]                           ----NTAG--------------------------VQ--------------SSQTSGVRAPATP--SPFQTLT--AAQLAALTLGVTSSLLPSPEELVQLSNAL-TALGASSSNVSQVALE 73
AAW30452.1_coat_protein_[Hydrangea_ringspot_virus]                     ----TTNP--------------------------IP------------------PQAGTSTPS--AAFAPLS--QEVLETLSFPVTSNLVPSPTELAAIADAW-TTLGVPAAETTKHALA 72
AAC54779.1_coat_protein_[Cassava_common_mosaic_virus]                  ----STTP--------------------------TT--------------AT-ITQAATTPLS--ALSTAPT--DEELSRLDLKPASNLVASADALSAIAADW-ASLKVPTAQLMRHALD 75
UTI93308.1_coat_protein_[Chaenostoma_potexvirus]                       -----------------------------------------------------------M--SLSKAPT--AEALKAMKFETTSTLVPTAAELDSISKEL-TDLKVGADQQLGHALA 53
CAA79765.1_coat_protein_[Plantago_asiatica_mosaic_virus]               -----------------------------------------------------------M--ALNTAPT--ADALAAMAFPVSSPSVPTAQELDTITSGL-TTLGVPTDSLLSHALA 53
BAC16789.1_coat_protein_[Tulip_virus_X]                                -----------------------------------------------------------M--ALNTAPN--PEALAAMTLEVSSPAVPTPAELDTIAAGL-TTLGVPADSLISHALA 53
QGT76802.1_coat_protein_[Citrus_yellow_mottle_virus]                   PTAVPVPPPDSKGKSAPIPEPIEKRISQAFHAEPKT--------------HNNGGPPAEFNPN--NMNAVPL--NLLNLNLRYSPVTNSIANPKQTEAIGKAWCRILHIDPSNVFLYAVD 173
AJO26403.1_coat_protein_[Citrus_yellow_vein_clearing_virus]            PIPVPTPSVTPTAAGKENQEPIEKRITHAFHAEAKT--------------HNNGVSPPAFNPN--NMNAVPL--NLLNLNLRYSPVTNSIANPKQTEAIGKAWVRILNIDPANVFLYAID 173
AAK97526.1_coat_protein_[Indian_citrus_ringspot_virus]                 PPPSSTPTTPTPPDQTKAPEPIEKRIIHAFHAEPKT--------------HTNGEAPPAFNPN--NMNAVPL--NLLNINLKYSPVTNSIANPKQTEAIGKAWVRILQIDPANVFLYAID 173
WVS18183.1_coat_protein_[Hibiscus_virus_X]                             P---ANPP--APANGAQRSTSAESGAARRYL-------------------REAAARESRRATS--SMHYVPT--MEEIRGLVLETTQASVATAGTVDEIGRFW-VSRGAPNNNVGLIALD 154
BAG06158.1_coat_protein_[Phaius_virus_X]                               -------------------------------------------------------MATTNTV--SKFGPAN--MQAWMDLKYEPTSESLVSEGELLKIQEQW-AAIGVT-GDFFTVAFQ 58
CAG24011.1_coat_protein_[Lily_virus_X]                                 ----------------------------------------------------------------MTTFVPD--AKTWADTAYTAQSESVATAEELQSIATLW-EGIGIPAANFFDVAFQ 52
AAW67750.1_coat_protein_[Mint_virus_X]                                 A---------------------------------AA--------------AAASAAKGKETAL--DSLGPPD--PQFLKDFSYVVATDSVATRGMVEAIRGKW-ERLGVPAASFFGAALQ 79
BAA02086.1_coat_protein_[Strawberry_mild_yellow_edge_virus]            ----GSNP--LPMGST-----------PPVLP--GR--------------TPNPNANVANQVG--DPFRVLT--PEELA-APISAASNKVATREQILGIVADL-NALGFVGD-PALGLFD 93
ASE06184.1_coat_protein_[Euonymus_yellow_vein_virus]                   T---GTPP--AAAR--------------RARRG-PV--------------ETGPNNAEEVNGE--SMTSQVN--MKFMEDIKYHSTSNNVADSIVLENIAADW-KSQGLPPQQCLRAAIE 151
CAA29908.1_coat_protein_[White_clover_mosaic_virus]                    ---------------------------------------------------------MATT--TATTPPS--LTDIRALKYTSSTVSVASPAEIEAITKTWAETFKIPNDVLPLACWD 57
CAN88812.1_coat_protein_[Lettuce_virus_X]                              ----PTP---------------------------VL--------------QRPPANSSGPNLA--DPNRAPS--MEDLAQVKYVSSSSAVATPAQITKLGEWF-VKVGIEPNSTGPAMWD 86
AAM76165.1_coat_protein_[Pepino_mosaic_virus]                          ----SAP---------------------------PT--------------AKDAGAKAPSDFS--NPNTAPS--LSDLKKVKYVSTVTSVATPAEIEALGKIF-TAMGLAANETGPAMWD 81
ALQ43530.1_coat_protein_[Plantain_virus_X]                             ----GSA---------------------------PP--------------QGKTPEPTPTDTS--DPTRGPS--LSQLRQVKYVSATTSVATPEEIKLLGSLY-ERMGIDKNEVAPAMWD 91
BAE44215.1_coat_protein_[Alstroemeria_virus_X]                         ----NTP---------------------------PT--------------PPTAPAGSAADLA--NPNRSPT--VSDLAKLNYQSTTTSVATPEEITALGVLF-TSAGVDPNSVPLAMWD 74
BAA02895.1_coat_protein_[Narcissus_mosaic_virus]                       ----ATP---------------------------ST--------------QTTDPKPANADLS--DPNRAPS--LEDLKKIKYESTTTAVATPAEIQLLGDLF-KKLGLDANSVAPAMWD 71
BAG12162.1_coat_protein_[Asparagus_virus_3]                            ----TKD---------------------------ST--------------STKAPEPVPVDLS--DPTRAPS--LKDLQAIKYVSTTTAVATPDEIKLLGDLF-KKLGVDGASVGPAMWD 74
ABG48664.1_coat_protein_[Malva_mosaic_virus]                           ----KKP---------------------------AE--------------NIPSQEPQPADPA--DPTRAPT--LEDLKAINYVSTTTAVATPAEIKLLGDLF-RKKGIDANAVAPAMWD 87
AAB57883.1_coat_protein_[Cymbidium_mosaic_virus]                       ------M---------------------------GE---------------PTPAPAATYSAA--DPTSAPK--LADLAAIKYSPVTSSIATPEEIKAITQLWVNNLGLPADTVGTAAID 68
AAB32335.1_coat_protein_[Potato_aucuba_mosaic_virus]                   T---SHA---------------------------GR--------------VQFLSAPKQFSAS--DVRSSPT--LTDLDEIAYEVRTTSIASPAEIEAVSQLWIMNTEIPADKVALIAID 95
WMX21795.1_coat_protein_[Adenium_obesum_virus_X]                       T---ERPY--TPLSFQ--------SILNRVMDT-SD--------------TDTASTVILSDLS--DPTISPS--LADLKRITYKTHSNSVASPETVKAIAKEW-VENGVPADQVALAAWS 116
AHA91822.1_coat_protein_[Cassava_Colombian_symptomless_virus]          T---RQPA-----------------------------------------------RPVTSNTAFNAG--DMCSAPS--LSDLDNVHHTSMNASIATSDQIKAIGALW-VQLGVPQASLAFTAWD 72
ARG47539.1_coat_protein_[Cassava_virus_X]                              -------M-----------------------------------------------ATTTTTTAFQAE--DMCAAPS--LDDLSKVTHLPSNSSVATPEQIRAIGALW-TQLGVPPAALAFTAWD 65
BAA00253.1_coat_protein_[Potato_virus_X]                               T---QATG--STTS------------------T-T---------------TKTAGATPATAS--GLFTIPD--GDFFSTARAIVASNAVATNEDLSKIEAIW-KDMKVPTDTMAQAAWD 82
QCY52828.1_coat_protein_[Euonymus_yellow_mottle_associated_virus]      N---QAPP--QNAG------------------G-GL--------------AANAPQG-PQGAT--PMSRAPT--LAVFEAFQYRVESNAVATPAQIDEISRVW-ATLGVPADSMFTTALD 121
AHB87037.1_coat_protein_[Ambrosia_asymptomatic_virus_1]                D---RGPP--PPSS------------------G-----------------RGTGQG-SGPPL--SMTTGPT--LTQLGQLKYKPQTTAVATRDQINYIAGLW-QKNGVPQDKLALAAWD 120
BBI37364.1_coat_protein_[Cnidium_virus_X]                              S---TAPP--PPPP------------------I-AP--------------QSNSQPSPTDFS--NPLAMPA--LKDLVA--AGSASNAVAEPSTISAIANSI-GLLGLNRANIAKGFWD 87
BAE66619.1_coat_protein_[Nerine_virus_X]                               ------------MA------------------N-TS---------------ASAALPAQSKTD--DMTAPPD--NKDLEALQYIPESNAVATADQIHAIAALW-KSIGVPAAKLGPYAWD 69
ASJ78786.1_coat_protein_[Vanilla_virus_X]                              ------------MA--------------------TP---------------APTPTP-ATNTA--DPFAEPS--ESDLKNLKVEIVSNAVATRAQITAIATAI-KAVGVADKDIAPTFWD 67
YP_009091818.1_coat_protein_[Yam_virus_X]                              ---------------------------------------------------MATPAP-KVDLS--DPLAAPS--NDDLTLQDFKTESNSVATTDQIKAIAAQL-IAIGVPSDTIASTFWD 63
                                                                                                                                                                       :
```

BAA05038.1_coat_protein_[Bamboo_mosaic_virus]	LSLEAFDRGSSEATTWDGI-TE----GVEHRAAANA-IKEANCPIHKVTYYLAKPTFAIRQSKNLPPANFAKKNVPSQYKWCAFDAFDGLYDPTCLASEL-PYDAPSEIDRMASATFKTI	204
AAA43830.1_coat_protein_[Foxtail_mosaic_virus]	FT---MDKGATDSTIFTGK-YN----TFPMKSLALR-CKDAGVPVHKLCYFYTKPAYANRRVANQPPARWTNENVPKANKWAAFDTFDALLDPYVVPSSV-PYDEPTPEDRQVNEIFKKD	174
AXK15644.1_coat_protein_[Turtle_grass_virus_X]	MAMEAMDKGATDSTVFTSK-SG----TFETKSLAMA-CKDAGVPVHKLCYFYTKPAFANRQKAGLPPARWANENVPVAYKWAAFDTATALFDPYVLESAI-PFDPPSAAAMQAHEVFRKD	193
WEQ03313.1_coat_protein_[Sclerotinia_sclerotiorum_alphaflexivirus_1]	FVMLCVDQGSSSATHYAGTIAG-VEAHTDVNEIARH-IK-QVCSLRKLCMYYAKYAFHLCVVHNRPPANWQRKGFTDSTKYAAFDFFNGVTNGAALNPPGGMVRQPTAEEITAHALNAHL	226
NP_932309.1_coat_protein_[Botrytis_virus_X]	FAIACAHQGSSRYTRHTGT-ST---GGSTFELIAAH-VK-EHCTIRQFCSYFAKVVWNHLLTHATPPVNWAKHGFTLDSRYAAFDFFDAVTNAAALPPKNGLIRAPTSEEIRAHNLNAHL	364
WLG65290.1_coat_protein_[Sclerotinia_sclerotiorum_alphaflexivirus_2]	LALHCADQGSSQFTRFPPSLSI---HSMNANALAEV-VK-SHCTLRQMCMYLAKAVFNHYVHTRKAPANWARKGFTYETRLASFDFFSGVTHSAAYEPPAGLLRLPTPEELKAHALNGQL	255
YP_008828154.1_coat_protein_[Donkey_orchid_symptomless_virus]	LAFACADQGASAYVIPVGK-H----GTMELSRAADI-VR-KVCTLRQFCMYYSKVVYDSMKSRDRAPANWVRKGFSYETRFAAFDFFSGTYSAAAPDPPYTLRYRPTPEEIAMHNKNAYA	166
NP_569136.1_viral_coat_protein_[Garlic_virus_C]	LAWACYHNGSSRYTNLATD-AP---CGMSHAELKDL-VE-EFCTLRQFCGFYAKTCYVTGRQQNKLPANWARKGFQDESKFAAFDFFNAVSSDSAPNPPGGMRFKPTQAEILGHSLNAKM	224
YP_009110672.1_coat_protein_[Garlic_virus_B]	LAWACYHNGSSKFTTLTTD-AP---CGMPHAELKDL-VE-DYCTLRQFCGYYAKTCYVTGKQQNKPPSNWSRKGFQEESKFSAFDFFNAVLSDSSPAPPGGMRFKPTQDEILGHSMNAKM	209
NP_044575.1_coat_protein_[Garlic_virus_X]	LAWACYHNGSSRFTTMTTD-AP---CGMPPAELKDL-VE-DYCTLRQFCGYYAKTCYVTGKQQNKPPANWSRKGFQEESKFAAFDFFNAVMSDSSPTPPGGMRFKPTHEEIVGHSMNAKM	208
NP_620652.1_coat_protein_[Shallot_virus_X]	LAWACYHNGSSRFVNLNTD-AP---CGITHADLKTL-WK-ASATLRQFCSYYAKSCYVSGKQQKKPPANWSRKGYPEEAKFAGFDFFNAVLSESSPAPPGGMRFKPTQAEILGHSMNAKM	227
YP_008855210.1_coat_protein_[Garlic_virus_D]	LAWTCYHNGSSRFVTLSTK-AP---CGMPLSELKDL-VE-NFCTLRQFCGFYAKACYVTGKQQKKPPASWSRKGYQDDAKFAGFDFFNAVLSDFSPAPPGGMRFKPTDAEILAHSMNAKM	215
NP_659014.1_coat_protein_[Garlic_virus_E]	LAWTCYHNGSSRFVTLATN-AP---CGMPHSELKDL-VE-NHCTLRQFCGFYAKACYVTGRQQNKPPANWSRKGYQDDSKFAAFDFFNAVLSDSSPVPPGGMRFKPTDAEILGHSMNAKM	281
NP_569130.1_viral_coat_protein_[Garlic_virus_A]	LAWTCYHNGSSRFVTLTTD-AP---CGMAHSELKDI-VE-NSCTLRQFCGFYAKACYVAGKQQNKPPANWSRKGYQEDSKFAAFDFFNAVLSDSSPSPPGGMRFKPTDAEILAHSMNAKM	216
YP_009508094.1_coat_protein_[Garlic_mite-borne_filamentous_virus]	LAWTCYHNGSSRFVTLTTD-AP---CGMAHSELKDV-VE-DYCTLRQFCGFYAKACYVTGKQQNKPPANWSRKGYQEDSKFAAFDFFNAVLSDSSPNPPGGMRFKPTDAEILAHSMNAKM	217
YP_009362673.1_coat_protein_[Alfalfa_virus_S]	LAWFCYHNGSSRFLVARGE-TS---HRIPLAKIKNV-VE-LKCTLRQFCMYYAKTCYNTGKAQMIPPASWSTRGYPENAKFAAFDFFSGVTNNAVPTPAGGMAYYPTAEEIASQSLNADL	248
YP_009389478.1_coat_protein_[Vanilla_latent_virus]	LAINCMHNGSSRLTMVDGA-TS---KSIPFTTIVDA-IK-QQCTLRQFCMYYAKVCYNIARERKIPPANWLAKGYSEDTKYAAFDFFAGVMNPASPSPPGGMKYTPSPAEMAASAVNGQM	198
YP_009328896.1_coat_protein_[Arachis_pintoi_virus]	LVWACFHNGSSRLTTLRGQ-SP---SGLELSYIKDV-VE-EHCTLRQFCTFYAKVCYNTAKELKTPPANWASKGFKEDTQYAAFDFFSGTMSPAAITPEGGMKYKPTPDEIKAHSANALM	196
QEM20973.1_ORF6_coat_protein_[Senna_severe_yellow_mosaic_virus]	VAWACYHNGSSVLTVLNAA-TP---SGITHAELKSI-IL-KECTLRQFATFYAKIVYSMGXEKKQAPANWMAKGFSEETKYAAFDFFSGVFSDAAPAPRGGMKWHPTANEIKAHNLNAKM	228
YP_004659204.1_coat_protein_[Blackberry_virus_E]	LAWACYHNGSSRYVTLEGS-SA---HGIPLASIKDV-VE-NHCTLRQFCMYYAKLCYNKGRSDKIPPANWAAKGFKAESMYAAFDFFNGVLNEAVPKPRTGMKFVPTEAEKAAHAVNAIM	200
WVN62093.1_coat_protein_[Rehmannia_allexivirus]	LAMSCYHNGASKYVTLEGS-SP---AGVPFSTLKDI-VE-QHCTLRQFCSYYAKYCYVLGKKTHTPPANWNRRGFMQEAQYAAFDFFNAVLSDAAPNPPGGMAFHPTPAEIKGHSLNAKM	547
YP_001718503.1_coat_protein_[Lolium_latent_virus]	IAIHCYHNGSSEQTDFNLK-SS-QVAGLNLEAAVGV-IK-EILTLRQFAAYYATFVWNWGIKNEIPPANWVAKGYTDETKYAAFDTFSYVGSPLGLR--ITPTRKPTNNEYMAASVNARE	258
CAF04082.1_coat_protein_[Hosta_virus_X]	LCMACYHSGSSGSTLIPGL-AP--GTTVNYTSLAAA-VK-SLATLREFARYFAPIIWNYAIEHKIPPANWAAMGYKENTKYAAFDTFDSILNPAALQPTGGLIRQPTEEELLAHQANSAL	181
ACN58198.1_coat_protein_[Allium_virus_X]	LTMFCYHSGSSPNTEITGD-SA--TPKVTLAQLAGV-VM-QHTTLRKFCRFFAPLVWNWALDHKIPPASWQANNFPPDEKYAAFDFFDGVTNGGSMLPADGLRRQPTPKELIAHATAQST	186
AEO12144.1_coat_protein_[Tamus_red_mosaic_virus]	LATFCYHSGSSTTTEMRGE-SS--KPGVSLSRLAGV-VN-MHTSLRKFCRYFAPLIWNIHVESKIPPAAWQSKDFKEGEKFAAFDFFDGVENVGSIKPPGGLIRKPTDKERIANATARAL	183
AUG45972.1_coat_protein_[Babaco_mosaic_virus]	LVNFCYDNGSSVYTVISGE-SS--ITGITLAQIASI-VKASGTSLRKFCRFFAPVIWNLRTD-KVPPANWESAGYKPTEKFAAFDFFDGVENPAAMQPPGGLIRSPNQAERIANQTNKQV	179
BAJ17501.1_coat_protein_[Lagenaria_mild_mosaic_virus]	LVNFCYDNGSSSYTNIQGP-SS--VEGVSLSQIASA-IKASGTSLRKFCRYFAPIIWNLRTD-QKPPANWEAAGYKPSAKFAAFDFFDGVENPAAMQPPQGLIRSPTQEERIANHTNKQV	170
ANW11496.1_coat_protein_[Senna_mosaic_virus]	LVNFCFDNGSSSYTAVSGQ-SS--IDGVTLNQLASM-VKASGTSLRKFCRYFAPVIWNLRVD-KVPPANWEAAGYKPGEKFAAFDFFDGVENPAAMQPPAGLKRSPTQAERIANATNKQV	167
AAX62023.1_coat_protein_[Alternanthera_mosaic_virus]	LVNFCYDNGSSTYTVVVGP-SS--LAEVSLSQVANI-VKASGTSLRKFCRFFAPIIWNLRTD-KTPPANWEANGFKPTEKFAAFDFFDGVENPAAMQPPGGLVRTPSQAERIANATNKQV	169
NP_044334.1_capsid_protein_[Papaya_mosaic_virus]	LVNFCYDNGSSAYTTVTGP-SS--IPEISLAQLASI-VKASGTSLRKFCRYFAPIIWNLRTD-KMAPANWEASGYKPSAKFAAFDFFDGVENPAAMQPPSGLIRSPTQEERIANATNKQV	177
NP_077083.1_coat_protein_[Clover_yellow_mosaic_virus]	IAWFCYHSGSSESVQVQGN-ST--SDKIPLYQLAGV-VR-QHSTLRRFCRYFAKVIWNYALRKNQPPANWASQNYKEADRFAAFDFFEGVSSSAALSPPGGLIREPSPNERMANETNKNV	173
AAR11551.1_coat_protein_[Opuntia_virus_X]	LVNYCYDNGSSPETLFRGN-SS---IGVPFSKLASI-IS-EHATFRQFARYFAPLIWNARISLNKPPAAWEAWHYNEEERFAAFDFFDGVLNSAALMPKDGLIRKPTRAEINASQTNRNV	191
QIL68844.1_coat_protein_[Papaya_virus_X]	VVNYCFDNGSSPDTTFKGT-SK--TLGVPLLKLANA-VT-QHTTLRQFCRYFAKLIWNYRLSTNKPPAAWEAWHYKPEQQFAAFDFFDGVLNEAALKPSDGLVRRPNELERLANQTNRNV	186
AFI57894.1_coat_protein_[Pitaya_virus_X]	LVNYCFDNGSSPETIFKGN-SA--TLGIPLSRVANA-VT-QHTTLRQFCRYFAKLIWNYRLEKNKPPAAWEAWAYKPEQKFAAFDFFDGVLNEAALNPTDGLTRIPNEAERLANQTNRNV	187
AY366207_coat_protein_[Schlumbergera_virus_X]	LVNYCFDNGSSPETVFKGN-SA--TLGVPLSKVANA-VT-QYTTLRQFCRYFAKLIWNYRLEKNKPPAAWEAWAYKPEQKFAAFDFFDGVLNEAALNPMDGLVRIPNEAERLANQTNRNV	187
AAK69583.1_coat_protein_[Cactus_virus_X]	IVNYCFDNGSSPETVFKGD-ST--VLQMPSPKSP-C-HH-PITTLRQFCRYFAKIIWNYRVSKNLPPAAWEAWAYKPEQKFAAFDFFDGVLNEAALNPIDGLVRVPNEAERLANQTNRNV	186
AAR11546.1_coat_protein_[Zygocactus_virus_X]	VVNYCFDNGSSPETVFKGN-SN--LLQIPLSKVAQA-IT-QVTTLRQFCRYFAKLIWNYRVSRNLPPAAWEAWAYKPEQKFAAFDFFDGVLNEAALQPTDGLVRLPNEAERLANQTNRNV	188
AAW30452.1_coat_protein_[Hydrangea_ringspot_virus]	LVNFCFDSGSSATTRFTGA-SP--TPTIPLSALAGA-VL-ELVPIRKFCRYFAQYIWNARLTANAPPASWEAWNFPENEKFAGFDFFDGVLNTASLKPPQGLVRKPTEAERVANATAKSL	187
AAC54779.1_coat_protein_[Cassava_common_mosaic_virus]	LVNFCFDSGSSKYTTVEGS-SP--TPTIPRAALAGA-VR-KHTTLRQFCRYYAKIIWNARVKANIPPAGYANAHIKPEQAFAGFDFFDGVMNVAALEPSGGLVREPTPQEIIAAETARSL	190
UTI93308.1_coat_protein_[Chaenostoma_potexvirus]	LVNFCFDSGSSKATVITGA-SP--TATVPLSQIAGI-VK-KHTTLRKFCRYFAKVIWNVRVSAAMPPAGYARANIKTEHQWAGFDFFDGLLNAAALNPQGGLTRDPSPAEITANETARSL	168
CAA79765.1_coat_protein_[Plantago_asiatica_mosaic_virus]	LVNACFDAGSSSFVTLSGP-SP--TPTISLAQIAGV-VK-VTTTLRKFCRFYAKIIWNARLARNLPPAGFARANIKFEHRWAGFDFFDGLLNPAALEPPGGLSRTPTPDEVTANETARSL	168
BAC16789.1_coat_protein_[Tulip_virus_X]	LVNACFDAGSSQFTTISGP-SP--TPTITLAQLAGV-VK-VSTTLRKFCRFYAKLIWNARLSRNRPPAGFARAYVKTGQKWAGFDFFDGLLNPAALEPLGGLTREPTPDEITANETARSL	168
QGT76802.1_coat_protein_[Citrus_yellow_mottle_virus]	LARACADAGTSPEADIIGA-NEDFDPVVERSALAGV-VR-DFCPLRAFCAYYSRVVWNLMIKADQPPANWMKSGVDESAKFAAFDFFHGVLSPASLY--VPLERHPTAAERIANQAMFAV	288
AJO26403.1_coat_protein_[Citrus_yellow_vein_clearing_virus]	LARACADAGSSPEADIIGA-NEDLNPVVERNALALV-VR-DFCPLRAFCAYYSRVVWNLMIKADQPPANWMKSGVDENAKFAAFDFFHGILSPASLY--VPLERHPTSAERIANQAMFAV	288
AAK97526.1_coat_protein_[Indian_citrus_ringspot_virus]	LARACADAGSSPEADIIGA-NEDLNPVVERNALAGV-VR-DFCPLRAFCAYYSRVVWNLMIKADQPPANWMKSGIDEGAKFAAFDFFHGVLSPASLY--VPLERHPTAAERIANQAMFAV	288
WVS18183.1_coat_protein_[Hibiscus_virus_X]	IAWQCKDNGSSRFTQLAGI-SP-YWPNTERYVITGH-IK-QFTTLRRFCMYYAKMIWVMMLEHQMPPANWVSLGYKEEHKYAAFDFFQGVLNGAALAPD-TLIRDPSPQEIQAHMNNASI	269
BAG06158.1_coat_protein_[Phaius_virus_X]	VAMVCSDAHSGSQTVLPGM-CN-QHPNIPLSAIGGI-IR-NVTTLRRFCRYYAKFVWNYRVANALPPANWAELEFTEETKYAAFDFFDGVTNVAALDPPNGLVRNPSEREFQASRINRYA	174
CAG24011.1_coat_protein_[Lily_virus_X]	LAMRCSDGHASSLTVLSGN-CT-VAPTVTLKAAAGL-VK-AVLPLRQFCRYYAKFVWNWRLSHDLPPANWADSQFPAEARFAAFDFFDGVTNSAAPQPPDGLIRPPTELELSAAQTAKFA	168
AAW67750.1_coat_protein_[Mint_virus_X]	LALACSDSHASSLTVLVGE-CA-AAPSVGLRDLAAS-VK-TICQLRHFCRFYAKFVWNYRVTHDLPPASWAAQGFPFEARFAAFDFFDGVTNSAALEPAQGLIRPPTEIELKAAHTGKFV	195
BAA02086.1_coat_protein_[Strawberry_mild_yellow_edge_virus]	LAFHCYDIGSSPSAQPVGP-SP-FG--CSRMQVAAV-VR-NHCTLRQLCMFYAPSVWNKAVRDNRPPGNWSNLQFTPETKFAAFDFFDGVLNPASQE--VPLWRQPTPQEIYASATHKDV	205
ASE06184.1_coat_protein_[Euonymus_yellow_vein_virus]	LTRYFADVGASEQTDVTGQ-GE-GI-DLEREVLAAT-VA-SHCTIRQFCSYYAKVVWNMMIYDDVPPANWARFNFPHEQRFAGFDFFEGVLSPAALEPRGGLLRKPSRDEINAYMTNKHV	266
CAA29908.1_coat_protein_[White_clover_mosaic_virus]	LARAFADVGASSKSELTGD-SA-ALAGVSRKQLAQA-IK-IHCTIRQFCMYFANIVWNIMLDTKTPPASWSKLGYKEESKFAGFDFFDGVNHPAALMPADGLIRGPSDAEILAHQTAKQV	173
CAN88812.1_coat_protein_[Lettuce_virus_X]	LARAYADVQASRSAMLTGA-SA-ADPNVSRQSLARQLDA-VFLTPRQFCMYFAKVVWNILLTTQVPPASWAKLGYSYDCRFAAFDFFDGVLNPAALDPADGLVRQPSPREIQAHQVAKSA	203
AAM76165.1_coat_protein_[Pepino_mosaic_virus]	LARAYADVQSSKSAQLIGA-TP-SNPALSRRALAAQFDR-INITPRQFCMYFAKVVWNILLDSNIPPANWAKLGYQEDTKFAAFDFFDGVTNPASLQPADGLIRQPNEKELAAHSVAKYG	198
ALQ43530.1_coat_protein_[Plantain_virus_X]	LARAYADVQSSQSAVLVGT-TP-SNPAITRQALSRQVLT-INVTPRQFCSYFAKVVWNMCIDSGIAPANWVKMGLQEPERFAGFDFFEAVLSPAALEPEGGLVRQPTMREIQAHALTKAG	208
BAE44215.1_coat_protein_[Alstroemeria_virus_X]	LARAYADVQSSRSAMLVGS-TP-SNKSITRQTLARKLDQ-VNITPRQFCMYFAKVVWNILISSNTAPAGWAKLGYPEECKFAAFDFFDGVLSPAALDPADGLIRQPNTREIQAHSTAKFG	191
BAA02895.1_coat_protein_[Narcissus_mosaic_virus]	LARAYADVQASRSAVLSGT-TP-SNPAITRQALARQFYV-INITPRQFCMYFAKVVWNLLLDSNVPPAGWAKQGLPDDCKFAGFDFFEGVLSPAALDPADGLIRPPSQREIQAHSTAKYG	188
BAG12162.1_coat_protein_[Asparagus_virus_3]	LARAYADVQSSRSATLSGT-TP-SNPAITRQALARQFYV-INITPRQFCMYFAKVVWNMMIDSNVPPAGWVKHGLPEDCKFAGFDFFEGVLSPSSLDPADGLIRQPSQREVQAHSTAKYG	191
ABG48664.1_coat_protein_[Malva_mosaic_virus]	LARAYADVQASRSAILSGS-TP-SNPSITRTALAKQLYS-IDLTPRQFCMYFAKIVWNMMLATHTPPANWAKQGLPEDCKYAGFDFFEGVLSPSALEPADGLIRMPNQKEIQAHSTAKYG	204
AAB57883.1_coat_protein_[Cymbidium_mosaic_virus]	LARAYADVGASKNATLLGF-CP-TKPDVRRAALAAQIFV-ANVTPRQFCAYYAKVVWNLMLATNDPPANWAKAGFQEDTRFAAFDFFDAVDSTAALEPAE-WQRRPTDRERAAHSIGKYG	184
AAB32335.1_coat_protein_[Potato_aucuba_mosaic_virus]	MARAYADVGASRKAVLLDA-PA-LAPTVARSRLAQLMAG-AGISPRQFCSYYAKIVWNLMLHKNEPPANWAKIGFKEDYKFAAFDFFDAVDSPAALEPSQ-WVRHPTDKERAAHGVVKWA	211
WMX21795.1_coat_protein_[Adenium_obesum_virus_X]	LAIYCADNGSSDQTEFEGS-IS-DL-SVSYDTLARI-TK-KHTTLRKFCSYYAKIVWNLLIERQSPPAGWAKWNYRKSEAFAAFDFFDAVTNPAALEPKEGLVRQPTQNELLAKGTNKMA	231
AHA91822.1_coat_protein_[Cassava_Colombian_symptomless_virus]	MARHCADIQATKTSTMIGI-SP-YC-GLARSLLAGT-IK-SVTTMRRFCSYYAKVVWNMLLKENKPPANWAKMGFLEETKFAAFDFFDAVLSPAALEPKGGLIREPTQLEITAYQTAKFV	187
ARG47539.1_coat_protein_[Cassava_virus_X]	LARHCADIQATKASTMIGI-SP-PC-NIQRSTLAGA-VK-SVTTMRRFCSYYAKVVWNMLLKENTPPASWAKMGFIEEAKFAAFDFFDAVRSPAALEPKGGLIREPTAIEVNAFQTAKFV	180
BAA00253.1_coat_protein_[Potato_virus_X]	LVRHCADVGSSAQTEMIDT-GP-YSNGISRARLAAA-IK-EVCTLRQFCMKYAPVVWNWMLTNNSPPANWQAQGFKPEHKFAAFDFFNGVTNPAAIMPKEGLIRPPSEAEMNAAQTAAFV	198
QCY52828.1_coat_protein_[Euonymus_yellow_mottle_associated_virus]	LARHCADVGSSPYVSLIGR-SP-PC-NLQRDALAAA-IR-TQCTLRQFCMFYAKFVWNLLKMSNTPPANWQKLEFRETEKFAAFDFFDGVLHEAALKPPQGVISPPSPAELAASKTNAYV	236
AHB87037.1_coat_protein_[Ambrosia_asymptomatic_virus_1]	LARHCADVGSSDAARMVDY-SP-AGQNITRQTLAGL-VK-TVCTLRQFCMFYAKVVWNMMLKTEKPPASWQKWEYRYTERYAAFDFFQGVSNEAALNPTDGLFRQPTDAEMAASHTNRYV	236
BBI37364.1_coat_protein_[Cnidium_virus_X]	IALHCADVGSSASVTLVGR-CE-PL-GETRAEIARV-IK-QHCTLRQFCMFYSRIVWNILIKEKRAPAGWMRHNYTEADRYAAFDFFSGVLNSASLQ--VPLIRQPTATEILAHNTNARV	200
BAE66619.1_coat_protein_[Nerine_virus_X]	LARHCADVQSSSQAKIVGT-PP-GESKLTRQMLAAS-IK-SICTLRQFCMYFAPVVWNMMILTDQPPANWSKLNYRESERFAAFDFFDGVTHTAALHPIGGLIRPPSEAERVANAAQKGL	185
ASJ78786.1_coat_protein_[Vanilla_virus_X]	VARHCADVGSSPSVILRGK-TP-FG--VERRQIAGI-IT-GHCTLRQFCMFYAKIVWNIMLLSDTPPANWQAQNYRVEDRFAAFDFFTGVSHTAALNPKSGLLRAPTPEEILANQTNARV	181
YP_009091818.1_coat_protein_[Yam_virus_X]	VARHCADVGSSAFITLVGT-TS-TG--VKRSTVAGV-IR-LNCTLRQFCMFYAKIVWNKMLLDDSPPANWQALNYNEAERFAAFDFFTGVTHKAALNPASGLVRQPTPAEILANQTNARV	177
	. . : : . : : * : ..** *.	

```
BAA05038.1_coat_protein_[Bamboo_mosaic_virus]                     QIKIANDQKGFNL-NYNPNVTQARLPNAPLPALPEPTSD-----------------   242
AAA43830.1_coat_protein_[Foxtail_mosaic_virus]                    NLSQAASRNQ-LL-GTQASITRGRLNGA--PALPNNGQYFIE----APQ-------   215
AXK15644.1_coat_protein_[Turtle_grass_virus_X]                    NLSQAAMRNQ-LL-GNQAAITRGRLDGA--PALPPPATYFIE----PPTTS-----   236
WEQ03313.1_coat_protein_[Sclerotinia_sclerotiorum_alphaflexivirus_1]  LIARSRQNDI-IS-TAAEITGMRTQAS-SIRARL----TYGPD-------------   262
NP_932309.1_coat_protein_[Botrytis_virus_X]                       LINASRQDDQVSS-SAQYTAAIAQAGG-FKRPQI----GWGE--------------   400
WLG65290.1_coat_protein_[Sclerotinia_sclerotiorum_alphaflexivirus_2]  LIAASRSELTLSN-SAAYTGAIAQAGG-FQHPQI----GWHSN------GN-----   294
YP_008828154.1_coat_protein_[Donkey_orchid_symptomless_virus]     LIEMSRRAGN-SS-TLGVITAAQQTQQPSRH--L----SLGNL------N------   202
NP_569136.1_viral_coat_protein_[Garlic_virus_C]                   SIIESRKATNMVS-TRADVIAQQQIHEPPKPLML----TF----------------   259
YP_009110672.1_coat_protein_[Garlic_virus_B]                      SIIESRKASNMVS-TRADLLAQQQIHEAPKPLML----TF----------------   244
NP_044575.1_coat_protein_[Garlic_virus_X]                         SIIESPKASNMVS-TRADSLAQQQIHEAPKPLML----TF----------------   243
NP_620652.1_coat_protein_[Shallot_virus_X]                        SIVESRQSSHMVS-TRADLLGRQQINEQPKPPMI----TF----------------   262
YP_008855210.1_coat_protein_[Garlic_virus_D]                      SIVESRRSSNMVS-TRADLLAQQQIHEQPKPPMI----TF----------------   250
NP_659014.1_coat_protein_[Garlic_virus_E]                         SIVESRRASNMVS-TRADILAQQQIHEQPKPPMI----TF----------------   316
NP_569130.1_viral_coat_protein_[Garlic_virus_A]                   SIVESRRATNMVS-TRADLLAQQQIHEQPKPPMI----TF----------------   251
YP_009508094.1_coat_protein_[Garlic_mite-borne_filamentous_virus] SIVESRRATNMVS-TRADLLAQQQIHEQPKPPMI----TF----------------   252
YP_009362673.1_coat_protein_[Alfalfa_virus_S]                     AIAGSRTNPHQYS-TRGNMMGMQQVRARDPPPMI----TFGE--------------   285
YP_009389478.1_coat_protein_[Vanilla_latent_virus]                AILEARQQEDQYS-TRGNMLAMRQVRATPRPPMI----TFE---------------   234
YP_009328896.1_coat_protein_[Arachis_pintoi_virus]                AIIDSRDQADQYS-TRGNMLAMRQERA-PPPPLI----TFQ---------------   231
QEM20973.1_ORF6_coat_protein_[Senna_severe_yellow_mosaic_virus]   AILESRQQDNQFS-TRGNMLAMQQVRTAPPPPLI----AFD---------------   264
YP_004659204.1_coat_protein_[Blackberry_virus_E]                  AITESRQQANQFS-NRGNMLAMQQIRTPAPPPLI----TFDN--------------   237
WVN62093.1_coat_protein_[Rehmannia_allexivirus]                   AIVESRQQENQHS-NRGNLAAMQQVNAAPTAPLI----TFPS--------------   584
YP_001718503.1_coat_protein_[Lolium_latent_virus]                 KIIQSRGKGM--V-TNSPMFSDGTTHQGIPLHP------KLP---------LS---   293
CAF04082.1_coat_protein_[Hosta_virus_X]                           HIFDSL--RNDFA-STDGRVTRGHITSNVNSLN------YLP----APEGSS----   220
ACN58198.1_coat_protein_[Allium_virus_X]                          NIFTASQNQLQTA-STHLLHTKGRFSTEPTKQY------YLP----GPEA------   225
AEO12144.1_coat_protein_[Tamus_red_mosaic_virus]                  KLFEAAAQQNRMA-SNSVHYTQGRYSDTAPQIQ------FLP----DPE-------   221
AUG45972.1_coat_protein_[Babaco_mosaic_virus]                     NLFQTAAQGNNLA-SNSAFITKGQISTSTPSIQ------FLP----SPE-------   217
BAJ17501.1_coat_protein_[Lagenaria_mild_mosaic_virus]             NLFQAAAQDNNFA-SNSSFITRGQLSTSSPTIQ------YLP----PPE-------   208
ANW11496.1_coat_protein_[Senna_mosaic_virus]                      NLFQAAAQDNNFA-SNSSFITRGQLSTNAPTVQ------FLP----PPE-------   205
AAX62023.1_coat_protein_[Alternanthera_mosaic_virus]              NLFQAAAQDNNFA-SNSAFITKGQLSSNSPTIQ------YLP----PPE-------   207
NP_044334.1_capsid_protein_[Papaya_mosaic_virus]                  HLFQAAAQDNNFT-SNSAFITKGQISGSTPTIQ------FLP----PPE-------   215
NP_077083.1_coat_protein_[Clover_yellow_mosaic_virus]             HLYQTASRGSNLA-TTSTVATKGAYSTNASNAG------FPY----HRPE------   212
AAR11551.1_coat_protein_[Opuntia_virus_X]                         HLFENKAQKSRYL-SNSALVTKGIADSEGPRVQ------FLP----GPE-------   229
QIL68844.1_coat_protein_[Papaya_virus_X]                          HLFENNVQKSRAL-STSTLVTKGVQGSDSPRVQ------FLP----EP--------   223
AFI57894.1_coat_protein_[Pitaya_virus_X]                          HLFESNSQKSRAL-TTSALVTKGLQGSESPRIQ------LLP----SPE-------   225
AY366207_coat_protein_[Schlumbergera_virus_X]                     HLFESNAQKSRAL-TTSALVTKGLQGSESPKIQ------LLP----SPE-------   225
AAK69583.1_coat_protein_[Cactus_virus_X]                          HLFESNAQKNRAL-TTSALVTKGLQGSESPRIQ------FLP----GPE-------   224
AAR11546.1_coat_protein_[Zygocactus_virus_X]                      HLFESNAQKSRVL-TTSALVTKGLQGSETPRIQ------FLP----GPE-------   226
AAW30452.1_coat_protein_[Hydrangea_ringspot_virus]                HLFEAATQRSNLA-STATQFTRGRLTDTSPTIQ------FLP----APE-------   225
AAC54779.1_coat_protein_[Cassava_common_mosaic_virus]             NLFEAQSKGNNLA-TNATQVTRGRLSSSEPQVQ------FLT----GVDE------   229
UTI93308.1_coat_protein_[Chaenostoma_potexvirus]                  SLFESNASGDNLA-STSTHLTRGKISSSAPQIQ------YLP----GPSD------   207
CAA79765.1_coat_protein_[Plantago_asiatica_mosaic_virus]          NLFEARASYSNLA-STSTQFTRGQLSNTAPQVQ------FLP----APSD------   207
BAC16789.1_coat_protein_[Tulip_virus_X]                           GLFESRANSNNLA-TTSTQFTRGQLSNTSPTVQ------FLP----SPED------   207
QGT76802.1_coat_protein_[Citrus_yellow_mottle_virus]              KIANAPGNGSELA-MDHVAFTKGRITQTSNQSK-------------TP-FAT----   325
AJO26403.1_coat_protein_[Citrus_yellow_vein_clearing_virus]       KIANAPGNGTDLT-MDHVAFTKGRTTQHSGLRP-------------TP-FNI----   325
AAK97526.1_coat_protein_[Indian_citrus_ringspot_virus]            KIANAPGNGSELT-MDHVAFTKGRITADSKPRP-------------TP-FNT----   325
WVS18183.1_coat_protein_[Hibiscus_virus_X]                        AYHDSVVHSGIKS-TTDLRITGGTIGMKPQLLI-------EG----PDD-------   306
BAG06158.1_coat_protein_[Phaius_virus_X]                          AIARVASSGFT---TTAAEVTKGRATAYQVHLL--------D----AP--------   207
CAG24011.1_coat_protein_[Lily_virus_X]                            ALARVRGSGF--V-TTAAEITHGRAEVSRTMLL--------S----PP--------   201
AAW67750.1_coat_protein_[Mint_virus_X]                            ALATSGTSSLT-L-SNHAAVTHGRAETARPTIL--------P----P---------   228
BAA02086.1_coat_protein_[Strawberry_mild_yellow_edge_virus]       ATYRAASKAHDRI-SNSTLLTKGASRSTPPALL--------P----GPDA------   242
ASE06184.1_coat_protein_[Euonymus_yellow_vein_virus]              HIARANKGRSRLG-TTAAELTHGELYEGEGA--Y----ELEN----P---------   302
CAA29908.1_coat_protein_[White_clover_mosaic_virus]               ALHRDAKPTWHKR-CQLC------------------------------------------   190
CAN88812.1_coat_protein_[Lettuce_virus_X]                         SLARQRISEGNFV-SSLSDVTHGRIGGINSM--Y----AIEA----PPEF------   242
AAM76165.1_coat_protein_[Pepino_mosaic_virus]                     ALARQKISTGNYI-TTLGEVTRGHMGGANTM--Y----AIDA----PPEL------   237
ALQ43530.1_coat_protein_[Plantain_virus_X]                        ALARQKIQNGNYI-SNLAEVTRGRVGGVNTM--Y----AIEE----PPEL------   247
BAE44215.1_coat_protein_[Alstroemeria_virus_X]                    ALARQRISNGNFV-STLAEVTHGRVGGVNAM--Y----SIEA----PPEA------   230
BAA02895.1_coat_protein_[Narcissus_mosaic_virus]                  ALARQRYRMETSFPPWLKSLTVGSAVSTPCTPLK----HLQNCNRNTSKLKLVCGL   240
BAG12162.1_coat_protein_[Asparagus_virus_3]                       ALARQRIQNGNFV-SNLAEVTRGRAGGVNSM--Y----AIEA----PPEL------   230
ABG48664.1_coat_protein_[Malva_mosaic_virus]                      SLARQRIQNGNYV-SNLAEVTHGRAGGVNAM--Y----AIEA----PPEF------   243
AAB57883.1_coat_protein_[Cymbidium_mosaic_virus]                  ALARQRIQNGNLI-TNIAEVTKGHLGSTNTL--Y----ALPA----PPTE------   223
AAB32335.1_coat_protein_[Potato_aucuba_mosaic_virus]              SLSRERLQEGTSI-TTVAELNKGHLGGYNNL--P----ALMA----PPS-------   249
WMX21795.1_coat_protein_[Adenium_obesum_virus_X]                  SLHEAAKQTLHTV-SNYAGVTQGRLPRPTLPA-------IEA----PEY-------   268
AHA91822.1_coat_protein_[Cassava_Colombian_symptomless_virus]     ALNRAEVHRGNLT-SNLAEVTRGQVGTKPTIQL------LN---------------   221
ARG47539.1_coat_protein_[Cassava_virus_X]                         AINRASVAQGNLT-STLAEVTRGQAGTKPTLQL------LN---------------   214
BAA00253.1_coat_protein_[Potato_virus_X]                          KITKARAQSNDFA-SLDAAVTRGRITGTTTAEAV----VTLP----PP--------   237
QCY52828.1_coat_protein_[Euonymus_yellow_mottle_associated_virus] AISRARQQQPTKL-TFAAEVNKGQLRVEEM-KY------LEA----P---------   271
AHB87037.1_coat_protein_[Ambrosia_asymptomatic_virus_1]           HVHRAGQGSSDYL-TLATEVHKGRLQGTRV-EY------LEA----P---------   271
BBI37364.1_coat_protein_[Cnidium_virus_X]                         SIYRTRIDQSQKA-TTAVEITGGTAR-PTPLL-------LLP----PP--------   235
BAE66619.1_coat_protein_[Nerine_virus_X]                          HIYKAAQQKNQLT-STAVEFTRAQVGSAPS-LT------LLP----PN--------   221
ASJ78786.1_coat_protein_[Vanilla_virus_X]                         SIFRARAQQNNKA-TTAVEVTGGQLTGGLNPVA------LSP----P---------   217
YP_009091818.1_coat_protein_[Yam_virus_X]                         AIFRSRAQSNNLA-TTATELTQGRITGGQNPIY------LEA----PEQ-------   215
```

Figure S2. Amino acid alignment of coat protein (CP) sequences of members of the family *Alphaflexiviridae*. Multiple sequence alignment of 71 CP sequences using Clustal Omega. Highlighted amino acids correspond with the more conserved region selected for the phylogenetic analysis of Figure 7.
