# Supplementary material for: Alphaflexiviridae in Focus: Genomic Signatures, Conserved Elements and Viral-Driven Cellular Remodeling
Source: Viruses. 2025 Apr 24;17(5):611. doi: 10.3390/v17050611 (PMC12115993; doi:10.3390/v17050611)
Supplement: Supplementary file 1 [file viruses-17-00611-s001.zip › viruses-3549170-supplementary/Supplementary_files/Figure S3.pdf]

CLUSTAL OMEGA (1.2.4) multiple sequence alignment

```
NP_619747.1__triple_gene_block_1_[Potato_aucuba_mosaic_virus]                 --------MEY---SFLVRLL--DHYGFERTTEKIVP-GQPIVVQGIAGCGKTTLLRNFHQEYPSIPIYSCFP------QKISENSEELQ-LLAK-ARF----TASAILDEYLAHKFDY-   93
NP_054026.1__triple_gene_block_1_[Cymbidium_mosaic_virus]                     --------MEL---AYLVRLL--EHNKFERTNLPL---SSPLVVHGIAGSGKSTILTTFHHHYPAYPIFSHSP------TLLDPSNRIYQ-QCITTDWV----PGGAIVDEYNYKALDY-   92
NP_663725.1__triple_gene_block_1_[Pepino_mosaic_virus]                        --------MER---STLINLL--QLHHFEP-KLSV---EGTIVVHGIAGTGKTTLLRTLFSAYPSLVIGSPRP------CYLDKQNKISQ-VCLSCFPN----THCDIVDEYHLLESFL-   91
YP_319828.1__triple_gene_block_1_[Alstroemeria_virus_X]                       --------MAL---FYLQTLL--LNSDFTRTTTPT---ASPLIIFGTAGCGKTSIIRALASQFPELHFSSFHP------IVLLPNIRKKQ-HLA--NPN----EATDVLDEFLAG-PNP-   89
YP_001960941.1__triple_gene_block_1_[Lettuce_virus_X]                         --------MAV---PLLHKLL--LENEFQRTATPL---SYPIIIFGTAGSGKTTIIRLLAAAYPDLHFSSFRP------CLLLPNTRRSQ-VAA--NPL----TPTDVLDEFLAG-PNP-   89
NP_040779.1__triple_gene_block_1_[Narcissus_mosaic_virus]                     --------MDC---KYLLELL--DSYSFIRSSRSF---SSPIIIHGVAGCGKSTIIQKIALAFPELLIGSFTP------ALLDSNSGRKQ-LAV---TS----DPLDILDEYLGG-PNP-   88
YP_001715613.1__triple_gene_block_1_[Asparagus_virus_3]                       --------MEI---SYIVDLL--NFLGFTRSSRPF---SLPLVVHGVAGSGKTHLLRKVSLHFPELVHCSFTP------QLIDPNSGRRQ-LPA---SE----TPTDLLDEYLGG-PNP-   88
NP_570727.1__triple_gene_block_1_[Scallion_virus_X]                           --------MEV---SYIVELL--DFLGFTRSRRPF---SLPLVVHGVAGSGKTYLLRKVSSHFPHLVHCSFAP------QIIDSNSGRKQ-APV---TS----EPTDLLDEYLGG-PNP-   88
YP_667845.1__triple_gene_block_1_[Malva_mosaic_virus]                         --------MEL---DYLIKLL--EFNNFPRTNLDF---SLPLVVHGVAGCGKSTIISKLAKAFPTLVVASFTP------QILDGNTGRKQ-VAV---DG----SPVDILDEYLSG-PTP-   88
YP_009186835.1__triple_gene_block_1_[Plantain_virus_X]                        --------MEL---EYLINLL--DFYGFVRSSRSF---SLPLVIHGVAGCGKSTIISKVSCAYPKLISASFSP------QLIDPDSGRRQ-SVV---DG----TTVDLLDEYLAG-NNP-   88
YP_001718500.1__triple_gene_block_1_[Lolium_latent_virus]                     --------MED-F----FNCL--RSRGFERTDEPLPSDSRRIVVHGVGGSGKTSLIEAFAIANEWVRAYTLTS-HER-FDISGRGISQYK-----GQPI--DEKLWTILDEYGQT-DNPE   95
NP_569127.1__triple_gene_block_1_[Garlic_virus_A]                             -----MLEMKT---DLLLQII--TNNDFSRTAEPL---TEPLVIHGVPGSGKSTLVKALI-LNRSTIAFTLGS-PYG-QTLTHPGVEKFTKGRPIPN---ISHCETRILDEYQLS-EGS-   99
NP_659011.1__triple_gene_block_1_[Garlic_virus_E]                             --------MKT---DLLIQTL--TNHQFQRTSEPL---QEPLVVHGVPGSGKSTLIKLLT-DCRSTFACTLGA-PYG-RNLASPGIR---SPRPTDN---LEDYETRILDEYQLG-VNR-   93
YP_008855207.1__triple_gene_block_1_[Garlic_virus_D]                          --------MKT---DLLLQIV--TNHGFERTEIPI---REPIVIHGVPGSGKSTLVKALL-KFQSTVACTLGA-PYG-RTLASPGIT---TPEPTSA---LTDYETRILDEYQLG-DES-   93
NP_620649.1__triple_gene_block_1_[Shallot_virus_X]                            --------MKT---DLLLQIL--SNNNFTRTSEPI---KEPLIIHGVPGSGKSTLVRALV-TYRSTVACTLGA-PYG-SNLAFPGVT---SPGLTQS---LTDHETRILDEYQLG-TES-   93
NP_569133.1__triple_gene_block_1_[Garlic_virus_C]                             -----MIEMKT---ALLLQLL--DNANFSRTTTPI---TDTIVVHGVPGCGKSTLIKDLV-TRHSTIAYTLGA-PYG-RSLIHSGVNH--ISRIGDN---LQDFETRILDEYQLG-NEE-   97
YP_009110669.1__triple_gene_block_1_[Garlic_virus_B]                          -MFPNVFEMKT---SLLIQLL--TNCGFQRTTVPL---EELIVIHGVPGCGKSTLIKTLI-THQSVVAYTLGI-PYG-KTLAHPGIQR--PTNIL-D---IQEAETRILDEYQLG-SKV-   100
NP_044572.1__triple_gene_block_1_[Garlic_virus_X]                             --------MKI---QLLIQLL--NNFGFHRTAEPV---REPLIVHGVPGCGKSTLIKTLI-THQSVSAYTLGV-PYG-KTLAHPGVLN--PPTLE-D---IFDFETRILDEYQLG-AKE-   93
WVN62090.1__triple_gene_block_1_[Rehmannia_allexivirus]                       --------MKT---NLFELHL--AAAGFQRTNLPL---SRPIVVHGVPGCGKSTLLIQLL-ADPNVRVYTCGV-PTG-ACLAHPGISD---QLPSSP---PPADQFVILDEYQLA-AEQ-   93
YP_009362669.1__triple_gene_block_1_[Alfalfa_virus_S]                         --------MNL---TQLETDL--LSAGFQRTQQPL---SFPVVIHGVPGCGKSTFIKAAL-ANPNTVARTLGA-PYG-DSLATEGVRT---HSPNES----FTQPLRILDEYQLG-ERS-   92
YP_009328893.1__triple_gene_block_1_[Arachis_pintoi_virus]                    --------MSL---TQLEDRL--ITAGFTRTNQPL---AYPIVVHGVPGCGKSTLIKTLI-EIVGFKAATTTQ-PYG-RTLSSCGVTS---HPLEDH----QRATIRILDEYQNV-APT-   92
YP_009389474.1__triple_gene_block_1_[Vanilla_latent_virus]                    --------MKI---LELEALL--LTAGFIRTNLPL---QSPITIHGVPGSGKSTIIKKLI-QRQDVIAYTLGA-PYG-KDLSQPGVLP---YTEATP---K-TTKFVILDEYQLD-LNI-   92
YP_004659201.1__triple_gene_block_1_[Blackberry_virus_E]                      -MFCLNLKMKL---NELDFLL--TSQGFERTNYPL---SFPLVIHGVPGCGKSTLIRTLL-VDINTAARTCGT-PYG-ATLQHPGVLA---PRDHPI---APSIDRRILDEYQLA-DTR-   100
YP_010798341.1__triple_gene_block_1_[Senna_severe_yellow_mosaic_virus]        --------MKI---VDVEREL--LKAGFTRTLNPL---RFPLVVHGVPGSGKSTLIKNLI-RDPAVKARTLGA-PYG-KNLVHAGVLT---HVRPSP---APCAEISLLDEYQLG-SEE-   93
NP_620716.1__triple_gene_block_1_[White_clover_mosaic_virus]                  --------MDH-I----HLLL--SAHGFTRTRLAK-S--KPIVVHAIAGSGKSTVIRKILSDLPTPKAYTLGK-PDP-YSLSNPTIKAFA-----QFKR----GTLDILDEYGQL-PLTD   90
NP_042584.1__triple_gene_block_1_[Bamboo_mosaic_virus]                        --------MDNRI----TDLL--TRSGYLRTSEPRGA-GQQLVVHAVAGAGKTTLLREILNTIPGVTIHTAGT-PDP-PNLTLNYIKGQE-----P-PC--P-KKYTILDEYPAR-AKW-   92
NP_040989.1__triple_gene_block_1_[Foxtail_mosaic_virus]                       --------MDSEI----VERL--TKLGFVKTSHTHIA-GEPLVIHAVAGAGKTTLLRSLL-ELPGVEVFTGGE-HDP-PNLSGKYIRCAA-----P-PV--A-GAYNILDEYPAY-PNW-   91
YP_009552763.1__triple_gene_block_1_[Turtle_grass_virus_X]                    --------MDLEL----TRRL--LASGYTRTEHPRAP-GAPIVVHAVAGAGKTTFLRSLL-EFRETEVYTAGT-HDP-PSLTGKHIRCAQ-----P-PT--P-GAFNILDEYPAW-PTY-   91
YP_010087745.1__triple_gene_block_1_[Euonymus_yellow_mottle_associated_virus] --------MDK-F----VSLL--TEFEFQRTREPISS-DRPLVIHGVAGCGKSTLVLKFLRLEPSASAFTSAK-ESA-RNLLGRQIEPIT-----SLPAEIPNDRILLLDEYPKA-N---   93
YP_002332930.1__triple_gene_block_1_[Potato_virus_X]                          --------MDI-L----ISSL--KSLGYSRTSKSLDS--GPLVVHAVAGAGKSTALRKLILRHPTFTVHTLGV-PDK-VSIRTRGIQKPG-------PI--PEGNFAILDEYTLD-NT--   89
YP_009389420.1__triple_gene_block_1_[Euonymus_yellow_vein_virus]              --------MEV-L----TRLL--NENRFLRTLEPI-S--KPLVIHACAGAGKSTIIRSVLNSVPGARAYTFGK-ADK-KNLSGQFIESAC-----CHPK--PEASFRILDEYLVS-D---   89
NP_042696.1__triple_gene_block_1_[Cassava_common_mosaic_virus]                --------MDS-F----IDEL--TVAGFTRTSLPF-S--KPLVVHAVAGAGKSTVLRNFCKLEPTAVAYTHGK-PDP-PNLEQTHIRVVQ-----N-PV--K-GHFCLLDEYTAQ-GVS-   89
UTI93305.1__triple_gene_block_1_[Chaenostoma_potexvirus]                      --------MDQ-L----HTAL--ENAGFERTKLPI-S--KPLVVHAVAGAGKSTLLRNFAAACPEALAQTCGP-ADP-PNLEQTHIREHF-----G-PQ--S-NKINILDEYPLL-EP--   88
NP_620837.1__triple_gene_block_1_[Plantago_asiatica_mosaic_virus]             --------MDS-I----INAL--TSNNFQRTNTPI-S--KPLVVHAVAGAGKTTLIQNLLPEHPNLAAQTAGS-PQT-PNLTGAFIRKLT-----C-PE--S-NKINLLDEYAAL-QP--   88
NP_702989.1__triple_gene_block_1_[Tulip_virus_X]                              --------MET-I----LTAL--EKAGFTRTKLPL-S--KPLVIHAVAGAGKTTLIQSLLAEHPNLSAQTAGV-PQN-PTLDGAYIRKLT-----I-PE--S-NKLNILDEYAAL-HP--   88
NP_077080.1__triple_gene_block_1_[Clover_yellow_mosaic_virus]                 MRFSQITSMNT-L----TNLL--LSEGYSRTTKPL-S--EPLVVHAVAGAGKSTLIRKLLAQSDAYRAHTNGP-PDP-PTLACTSILPFT-----SNPP--Q-HTFNILDEYPIG-Q---   96
YP_002647028.1__triple_gene_block_1_[Allium_virus_X]                          --------MNT-L----VDTL--VEAGFIRTHEPL-S--NPLVVHAVAGAGKSSLIRRLLETDATFRAFTRGP-PDP-PSLDATAIQAFQ-----PNPP--S-HLFNILDEYPAG-E---   88
YP_004849315.1__triple_gene_block_1_[Tamus_red_mosaic_virus]                  --------MNS-V----FELL--ESEGFERTKEPI-S--KPVVVHAVAGAGKSTLIRKLLERNPEIRAFTHGV-PDP-PSLGCNDIGPFQ-----SNPP--S-HTINILDEYPAG-D---   88
YP_224085.1_triple_gene_block_1_[Hydrangea_ringspot_virus]                    --------MDT-L----VSEL--QRLGYTRTNLPL-S--RPLVIHAVAGAGKSTLLRHLLTLPQPFYVQTHGI-PDP-PNLSQRYIRPAN-----V-PL--S-NHFNILDEYLRS-Q---   87
YP_002308465.1__triple_gene_block_1_[Hosta_virus_X]                           --------MAT-F----ASFLSSTRPDFERTNTPL-T--KPLVIHAVAGAGKTTLLRDFLRANPLTNAQTLGT-PDC-PTLDGAYIRPFS-----G-PV--A-NLVNILDEYTAH-----   88
YP_054408.1__triple_gene_block_1_[Opuntia_virus_X]                            --------MEN-L----VAML--TANDYTRTDRAF-S--KPLIVHAVAGAGKTSLIRRFMKENAQVSAQTHGT-PDP-PNLTRKMITKVT-----A-PK--S-NHFNILDEYCAF-P---   87
QIL68841.1__triple_gene_block_1_[Papaya_virus_X]                              --------MEK-L----IEIL--TADGYSRTDLPI-S--KPLVIHAVAGAGKTETIRRFLKVWPATNAQTLGT-PDK-PNISRKMIRAFT-----G-PK--A-NMFNILDEYCAV-P---   87
NP_148781.1__triple_gene_block_1_[Cactus_virus_X]                             --------MET-L----TALL--IANGYTRTDLPI-S--KPLVIHAVAGAGKTTLIRQFLHQHSATNAQTLGT-PDK-PNLSRKMIRQFS-----M-PK--A-NHFNILDEYCAQ-P---   87
YP_054403.1__triple_gene_block_1_[Zygocactus_virus_X]                         --------MEH-L----ISLL--IANGYTRTDTPI-N--KPLVVHAVAGAGKTTVIRQFLKECPAVNAQTLGT-PDK-PNLTRKMIREYS-----M-PK--A-NHFNVLDEYCAQ-P---   87
YP_009046883.1__triple_gene_block_1_[Pitaya_virus_X]                          --------MDR-L----IDLL--TANGYSRTDHPI-S--KPLVIHAVAGAGKTTLIRQFLKENPAINAQTLGT-PDK-PNLSRKMIRPHT-----G-PK--A-NHFNILDEYCAQ-P---   87
YP_002341560.1__triple_gene_block_1_[Schlumbergera_virus_X]                   --------MDA-L----ISLL--TTNGYSRTDLPI-S--KPLVIHAVAGAGKTTLIREFLKLHSSINAQTLGT-PDK-PNLSRKMIRQYS-----M-PK--A-NHFNILDEYCAQ-P---   87
YP_009664729.1__triple_gene_block_1_[Lagenaria_mild_mosaic_virus]             --------MNL-F----IEML--KENGFERTGLPL-S--SPLVVHAVAGAGKTTLIRKYLASNPNSLAFTHGI-PDR-RNLENRYLREFD-----S-PV--P-GFFCILDEYPGG-E---   87
NP_044331.1__triple_gene_block_1_[Papaya_mosaic_virus]                        --------MNH-F----INLL--VAEGFVRTNEPL-T--DQLVVHSVAGSGKSTLIRKFLEEQPLARAYTHCR-ADP-PNLEGRFIQPFK-----G-PC--P-DHFNILDEYCKE-P---   87
YP_009270631.1__triple_gene_block_1_[Senna_mosaic_virus]                      --------MNH-F----INAL--TEGGFERTKVPI-S--EPLVVHAVAGSGKTSLVRSFIKDYNVARAYTHGV-PDP-QNLEGKFIQAYK-----Q-PN--P-THFNILDEYCAE-P---   87
YP_459945.1__triple_gene_block_1_[Alternanthera_mosaic_virus]                 --------MNH-F----INLL--IEEGYVRTNEIL-S--DTLVVHAVAGAGKSTLIRKFIHQHPQARAYTHGV-PDP-PNLEGRFIQAFK-----N-PD--P-NHFNILDEYCAE-P---   87
YP_009448188.1__triple_gene_block_1_[Babaco_mosaic_virus]                     --------MNH-F----IEVL--TAQGFVRTNEPI-S--EPLVVHAVAGAGKSSLVRQYISENPNSRAFTHGV-PDP-PNLTGRFIRPFR-----G-PI--E-GCFNILDEYSAE-P---   87
YP_010087352.1__triple_gene_block_1_[Cnidium_virus_X]                         --------MSN---TLLFNLL--ARAEFERTDTPS---STPIVIHAVAGAGKSSLLRQLTAYYKVGTAAK----PDP-PSFKGDHF---------TACV----EGIDILDEYQSA-QTI-   84
YP_009091815.1__triple_gene_block_1_[Yam_virus_X]                             --------MSN-FRTNIVELF--TAQGFERTSLPI---STPIVIHGVAGSGKTRILEDVLDKFPVSVYSNIP---KP-KRFNGRNF---------TEKI----AGALIVDEYQLG-FT--   86
WEC26234.1__triple_gene_block_1_[Nerine_potexvirus_1]                         --------MEN-I-----LQL--LNLHFQRTNLPL---SKPVVIHSCAGAGKTTLIQTILQNHPSATAYTHSTHYASSRDLAGYNLRPF-----SQYTP----GHLDIIDEYLAG-SIP-   90
YP_446993.1__triple_gene_block_1_[Nerine_virus_X]                             --------MEN-I-----INK--LQTRFTRTSLPI---SKPIIIHCCAGAGKTTFIRELLEQFPEVQAYTHSQHLAAEKTISGRNFHHF-----SEYQP----GYLDVIDEYLAG-PIP-   90
YP_010087333.1__triple_gene_block_1_[Ambrosia_asymptomatic_virus_1]           --------MDV-I----ISKI--LESGFTRTDEPISE-GNQVVVHAIAGSGKTRLIKELLDAHNFIRAYTHGPVPS--RGISGRSVIKATSQPPQGLPE----GTYTILDEYLAG-PS--   95
QJX15395.1_triple_gene_block_protein_1_[Carnation_latent_virus]               --------MEV-V----CKVL--ADRGFQRVRSKL---SVPIIVNCVPGAGKTSAIFDILASDSRFKAVTFGA-KQE-RKIEGLIIDRVA---SGCRPP----AEFLIVDEYTEG-DYG-   91
NP_620643.1__triple_gene_block_1_[Strawberry_mild_yellow_edge_virus]          -------MVEF-T----KRLL--LERNFERTNRPI---TGPIVVHAVAGAGKSSVINTVSLTFQLICWTTLPEEKASFNCLHLRHL---------DGPA----FPGAFVDEYQLA-DTD-   88
YP_009389480.1__triple_gene_block_1_[Vanilla_virus_X]                         --------MAD-YTTNIENFL--VSLGFIRTPTPLNHKTRPIVFHAVAGAGKTTALIKIIQDYPVTVATNSD------TLCLTSGAQIV-----KYTPG----VRYNIVDEYQLC-PEI-   92
WVS18184.1__triple_gene_block_1_[Hibiscus_virus_X]                            --------MDQ-L----IRLL--EQHGFQRTSQPL---THPLVVHAIAGAGKTSCLRDLARASNEFQITTQGL-PLA-RDL-DSAGSG-------TNPD----AAIFILDEYQAA-DSW-   86
YP_010797204.1__triple_gene_block_1_[Cassava_Colombian_symptomless_virus]     --------MER-I----IEEL--NREKFIQVSNIT---EYPIVVHAVAGAGKTTLLHRLLKDLDL-RICTTAQ-PSG-FSILGRKISR-------FSIS----EPCDILDEYPGA-EHP-   86
YP_009357227.1__triple_gene_block_1_[Cassava_virus_X]                         --MCSHGLMDA-I----LREL--KQSGFVQIRNHL---QNPLVVHAVAGAGKTTLLNKLAACSDL-IIHSAAY-PSG-NSLSGNSVQI-------HNPS----VTPDILDEYLLV-PDY-   92
WMX21792.1__triple_gene_block_1_[Adenium_obesum_virus_X]                      --------MDN-L---VITEL--TRLQLTRTSRPL---SKPIIIHLVAGAGKTTIIRKLLTIHHTIRAFTCGT-PDP-PSLTGTHILPW-----KSERP----GTTTLLDEYPLA-TDY-   90
YP_010798309.1__triple_gene_block_1_[Citrus_yellow_mottle_virus]              --------MDF-A-----ELL--SSKEFTRTKLPL---SEPIVIHAIAGAGKTHLLEQYSRHNPSNTFFTPLD-NKS-NSLLLSPF---------SSDC----SSASFIDEYPLC-EIP-   84
YP_009124989.1__triple_gene_block_1_[Citrus_yellow_vein_clearing_virus]       --------MDL-P-----ELL--LSKNFIRTRLPL---AKPIVIHAVAGAGKTQLLEEFARSSPSTKIYSPVK-HHS-NSLLLSPF---------HKAL----SEASIVDEYPLS-QIH-   84
NP_203554.1__triple_gene_block_1_[Indian_citrus_ringspot_virus]               --------MDF-A-----ELL--ESKAFTRTRLPL---SKPIVVHAVAGAGKTSLLENYARINPAARIYTPIA-QQS-NSLLLSPF---------TQSL----EQADIVDEYPLS-TLH-   84
YP_224135.1__triple_gene_block_1_[Mint_virus_X]                               --------MDS-Y-----RAE--LASAFTRTSLPL---SKPIVVHTVAGAGKTTFIRRLIRHAPFPTAITGGT-PDP-PHISGQRI---------TAPP----GPANIVDEYPLV-DW--   83
YP_263304.1__triple_gene_block_1_[Lily_virus_X]                               --------MEF-C-----GGT--LRTRFQRTSVPN---SPVTVVHTVAGSGKTTFIRNLLIDHPQLVARTYGT-PDV-PNLLGYGI---------RDRH----GDAHIVDEYPAA-DLR-   84
YP_001655011.1__triple_gene_block_1_[Phaius_virus_X]                          --------MEL-Y-----TGE--LQQKFHRTNVPL---KFPIVLHTVAGAGKTTFVRQLINKYSHLTACTFGT-PDP-PNLLGRYI---------TAPC----PFPDVVDEYPLC-EEY-   84
                                                                                      :                                  .    * **:  :             .                                     .:**:
```

```
NP_619747.1__triple_gene_block_1_[Potato_aucuba_mosaic_virus]    --QKC-----LAVFADPLQYS-HLGALRPHYQTSKHIGLVLVLLILSLR-NWIPIESLL--SE---EKTILKECDPYATD-------PIGQIIASNHEVLNYIKPQAVEAICSCEVLGKE   192
NP_054026.1_triple_gene_block_1_[Cymbidium_mosaic_virus]    --SRC-----LAVFGDPLQLP---HSLQPHYYSSRTHRYGPKLTSLLNDLFHLSITS-L--AP---V-DSLDYADPFAVD-------PSGFTIAD-EEVYNFVSQQVPGTLLPLDTVGLE   187
NP_663725.1__triple_gene_block_1_[Pepino_mosaic_virus]    --EPK-----LAIFGDPCQCTYIERLRVPHYTSFRTHRFGKSTAEILNKLFDLNIVS-V--KK---EDDIVEFFNPFEVD-------PTEHISASEEEVLDFVSDQ-VVTTSSEELAGLE   190
YP_319828.1__triple_gene_block_1_[Alstroemeria_virus_X]    --EVR-----IAKFCDPLQYN-CETLPEPHFISETTYRFCPRTCELLNDIFKTTLKS-K--VL---EICKVACVDPYAVD-------PVGKVIAIEQELFPILSAHGLTVHSPDFLTGQT   188
YP_001960941.1__triple_gene_block_1_[Lettuce_virus_X]    --AVS-----LAKFCDPLQYN-CTDLPLPHYTSNHTYRFCPATCDLLNQLFSTTLSS-R--LP---TAASITRPDPYSQD-------PSGTVVALEQDLLDILKQHGAFPKKPAELTGQT   188
NP_040779.1__triple_gene_block_1_[Narcissus_mosaic_virus]    --VVR-----LAKFCDPLQYS-CEQPEVPHFTSLLTWRFCVRTTALLNGIFGCQIKS-R--RE---DLCHLTHENPYTTD-------PKGVVVAHEQEVINLLLQHGCPVTPTQHLWGLT   187
YP_001715613.1__triple_gene_block_1_[Asparagus_virus_3]    --VVR-----LLKVCDPLQYN-CPDPEIPHFQSLTTRRFCPLTTTLLNSLFGTNIVS-A--VP---TCCRIEIQDPYSTD-------PIGTVVTFSPEIHTLLSRHGCQPTPISELWGLN   187
NP_570727.1__triple_gene_block_1_[Scallion_virus_X]    --LVR-----LLKVCDPLQYD-CPEPEVPHYVSLTTRRFCPLTTTLLNSLFGCDIVS-L--VD---RNCHIQIHDPYTDD-------PIGTVVSLSPDIGALLLRHGCATTPIADLWDVN   187
YP_667845.1__triple_gene_block_1_[Malva_mosaic_virus]    --SVR-----LALFCDPLQYS-CEKPRLPHFISLTTHRFCPLTADFLNSKFGCEIIS-L--RQ---DSCEIVEADPFATD-------PEGVVITFEPEVKSILERHQCFPTDISTLWGKN   187
YP_009186835.1__triple_gene_block_1_[Plantain_virus_X]    --IVR-----LAKFSDPLQYD-CQSPEEPHYRALHSHRFCPATAQLLNKIFGCHITS-K--LN---TSAVIRFADAFTED-------PEGQVITFEPELESLLTAHGCPTTPVADLWGRN   187
YP_001718500.1__triple_gene_block_1_[Lolium_latent_virus]    -----TLPPFNVLATDPYQAFRC-QPLRAHFVSLRSYRVPHHIAQAITQYTGFPIEAAGTDLH-----EGKYTVGPWTDK-------LRQQILVEDGEIHYQLSRRQCPHKLITDAIGEQ   197
NP_569127.1__triple_gene_block_1_[Garlic_virus_A]    -----DTSPFNLLIGDPFQGTL---HLKAHYIKSHSYRVPAPICHFLR-TLDYEIFGS---RP-----GEIVKLPVYSKNPT----APSGQVLHLGAASCSLTRRHNVHSKSPADVQGLE   198
NP_659011.1__triple_gene_block_1_[Garlic_virus_E]    -----DISPFNVLVGDPFQGTL---HFRAHFTKNLSHRVPRTICTYLR-LFDFEIFGE---NQ-----GTISFPPVYSDQPS----TPRGRVIHLGNVSRDLTRSHNICSLDPAAVQGLE   192
YP_008855207.1__triple_gene_block_1_[Garlic_virus_D]    -----IAVPFNILIGDPFQGHL---HYRAHFVKRVSHRVPKSVCDFLT-TLDYDITGT---SD-----GDVVQLPVYSSAPG----PPLGQVLHLGLASRQLTKSHNVCSRAPSEVQGLE   192
NP_620649.1__triple_gene_block_1_[Shallot_virus_X]    -----DLKPFNVLVGDPFQGNL---HLKAHYVKSFSHRVPRIICNFLQ-SLGYEIAGS---KP-----GELAQLPIYGPNPS----GPTGQVLHLGPLSRRLTQSHGVCSKLPSEVQGLE   192
NP_569133.1__triple_gene_block_1_[Garlic_virus_C]    -----NVKDFTILFGDPFQGTF---QLPAHFVKRLSHRVPRQICNYLE-SLDYDITGE---TE-----GEINFPPIYAANAT----GPIGTVLHLGPISRQLTKTFGICSKLPTEVQGLE   146
YP_009110669.1__triple_gene_block_1_[Garlic_virus_B]    -----DLEPFNVLIGDPFQGHS---TYRAHFVKTFSHRVPRPVCEFLN-SLGYDIQGD---RE-----GDLKFVPVFQNNSK----GPKGVVIHLGPISCQLTRTHRVPSKTPSEVQGLE   199
NP_044572.1__triple_gene_block_1_[Garlic_virus_X]    -----DFKAFNILFGDPFQGDT---HLPAHFIKSLSHRVPRQICQFLN-SLQYEIQGE---RE-----GTLNHLPVFEANST----GPRGVVLHLGPISCQLTRTYRVASKTPAEVQGLE   192
WVN62090.1__triple_gene_block_1_[Rehmannia_allexivirus]    -----QLDTFHAVLGDPFQGPL---SRRAHFTKSYSHRVPRPVAELLT-AFDFTIQSD---VE-----GQVEAVRVHSGNEA---AFLTPTCLHLGAISRDLAHSHGICSKAPREVQGRE   193
YP_009362669.1__triple_gene_block_1_[Alfalfa_virus_S]    -----TTSKYNLLFGDPYQGPF---RLPPHFIKSVSHRVPRPVANFLR-SRGFNLESE---TP-----GVLITAHPFHPDSATQ-ILSSDKTFHLGTSSKQLCSSHQVNSYCPSELAGSE   194
YP_009328893.1__triple_gene_block_1_[Arachis_pintoi_virus]    -----EWEKYQVLIGDPFQGPY---SSVAHYVKATSHRVPKQVADFLR-ARSFDIYSD---KE-----GSLTTTNPYSPKVAQQ-F-QNATILHLGPISKQLLQSHSVPSICWKEFAGQE   193
YP_009389474.1__triple_gene_block_1_[Vanilla_latent_virus]    -----TTEGISAIFGDPYQGNT---DRPAHYISIISHRVPKPVTDFLT-TLDFEIQSF---KP-----GTLTTVHPYKATQFQIT--PEIPVIHIAEISQALLSSHSVHSHSPQEVSGLE   193
YP_004659201.1__triple_gene_block_1_[Blackberry_virus_E]    -----TIADFNVLFGDPYQGSF---RLSPHYTKSLSHRVPKPVCEFLR-TRGFDIAGE---RS-----GRITTANPYSPELN-STDWLKHTILHLGPASAALTHSHAICSRSSAEVQGLE   202
YP_010798341.1__triple_gene_block_1_[Senna_severe_yellow_mosaic_virus]    -----DTVGYTILVGDPFQGPF---SLPAQFIKEISHRVPKVVCNFLT-TRGYSISGE---RP-----GSLTTRPATITTIDAHTNPCKGTVFHLGEASKXLTHSHGICSSHPTEKSGLE   196
NP_620716.1__triple_gene_block_1_[White_clover_mosaic_virus]    LDSS-----FEFIFTDPYQAPTD-NLFEPHYTLETTYRFGPNTCNLLNQAFQSNITSL---VT----KDNISFGSPYLVD-------PVGTILAFQPDTYLILCLHQASFFKVSDVIGYQ   190
NP_042584.1__triple_gene_block_1_[Bamboo_mosaic_virus]    PEEAW-----DMLIADNLQHTG--PTTRPHYIKHTTYRLGPQTVKTLQ-SLGYQLEFQ---HRADQQDQGFSFTGLFDGP-------IYGQPITLDTAAHNLALAHGLPALQATQTRGLE   194
NP_040989.1__triple_gene_block_1_[Foxtail_mosaic_virus]    RSQPW-----NVLIADNLQYKE--PTRRAHYTCNRTHRLGQLTVDALR-RVGFDITFA---GT-QTEDYGFQEGHLYTSQ-------FYGQVISLDTQAHKIAVRHGLAPLSALETRGLE   192
YP_009552763.1__triple_gene_block_1_[Turtle_grass_virus_X]    TSEHW-----QALFADNLQHSG--PTLRAHYTCSLTYRFGEQTAEALR-HIGFTITPR---PD-SNPCAGLSWDNLYVGY-------IFGQVITLDQEAHRLATAHGLSPITAEESRGLE   192
YP_010087745.1__triple_gene_block_1_[Euonymus_yellow_mottle_associated_virus]    LRDPWNLKSFQVLLCDPLQFQE--QVLPAHYISNVSKRFGKNTCDLLKAKLDITCSSS---RF-----DSVTEASCYEVD-------PEGHVIGLDTDICHLARQHSLSTDDPTNCLGQT   196
YP_002332930.1__triple_gene_block_1_[Potato_virus_X]    TRNSY-----QALFADPYQAPE--FSLEPHFYLETSFRVPRKVADLIA-GCGFDFETN---SQ---EEGHLEITGIFKGP-------LLGKVIAIDEESETTLSRHGVEFVKPCQVTGLE   188
YP_009389420.1__triple_gene_block_1_[Euonymus_yellow_vein_virus]    DGEEY-----DAVFCDPLQVKG--TARRPHFICTTSQRFGWHTADLLR-KLGIELNSS---KE-----DLVLIQPLFEGE-------PEGVILAWEPEVCALLDDHLVEFKKPSEVIGET   186
NP_042696.1__triple_gene_block_1_[Cassava_common_mosaic_virus]    NIEGW-----SALLADPLQHDS--PALRPHFVKYLSHRICPKTATLLQ-ELGIKIQSA---RQ---DEAEINFTGLFDGP-------IIGTVIHLDQIAKKLASSHGIHSYCPRELRSFE   188
UTI93305.1__triple_gene_block_1_[Chaenostoma_potexvirus]    LRGAW-----AAVFADPLQYST--GALPPHYVKKTSHRLGVHTAQLIS-KLICPVSSS---ST---KPDSVTFSGIFEGQ-------LLGQIIALDDLTASLLARHSVPFACPEQVRSQE   187
NP_620837.1__triple_gene_block_1_[Plantago_asiatica_mosaic_virus]    LKGSW-----DVVLADPLQHPG--LALRPHFIKSVSHRLCPATTRLIS-KLVCPCTSS---RT---EESTIQFSGLFEGP-------LLGTVIALDQTTQALLTAHGAHFLCPTAALGLE   187
NP_702989.1_triple_gene_block_1_[Tulip_virus_X]    LKGSW-----DVVCADPLQHPN--TALRPHFLKTTSHRLCPATTGLIS-KLICPCSSS---RP---EPTKITVTGLFESP-------LFGTIISLDDTCHKLLQAHGLQPHCPESVLGLE   187
NP_077080.1__triple_gene_block_1_[Clover_yellow_mosaic_virus]    SKG-Y-----KALFADILQHRN--NHQSPHFIKTTSHRLGPSTAKLIRETLNIPLEGT---GD----AELKSILPVLGSP-------LFGILIALTPEVATLLKAHGAKFYCPDQVLGLE   194
YP_002647028.1__triple_gene_block_1_[Allium_virus_X]    VKGPW-----AALFADPLQHRA--HPRRPHFIKRCSHRLSASTAALLT-SLGIPITGT---GQ----GNFTSAHGVFEGP-------LIGKIISLDNHISLLLSNHSVPYATPDDVLGQE   186
YP_004849315.1__triple_gene_block_1_[Tamus_red_mosaic_virus]    VSGAF-----HVLFADPLQHRK--PKRRPHFVKRVSHRLGPNTENLLR-KVGISITGA---GS----AEVKTQQSIFEGA-------LFGKITAVDSNVGNLLANHGVKFSYPDDVLGLE   186
YP_224085.1_triple_gene_block_1_[Hydrangea_ringspot_virus]    SKDPG-----TPSSRTP-SNTQ--KTASPHFTRNVTHRFGPDTCSLLS-SLGIDIVSA---PD--SEQDTLTLSPIFEGT-------LQGQILALDTDCARLLEAHGVPFLCPRRVLGLE   186
YP_002308465.1__triple_gene_block_1_[Hosta_virus_X]    RHGSW-----DVLIADPLQHYE--RAKLPHYICKRSHRLCPATARLLR-KLGLDIHSY---RE---DESEISFSDIFSGQ-------LEGTVLPLTPLCKDLLERHSCPFKCPSEFIGEQ   187
YP_054408.1__triple_gene_block_1_[Opuntia_virus_X]    LKGSW-----DACFADPLQHPD--FAQEPHFIKETTHRFGPETCELIK-SLGIPICPL---GP----SQVLTIKGLFEGE-------IFGKVICLDSDIAALACRHRIPISQPKQTIGLE   185
QIL68841.1__triple_gene_block_1_[Papaya_virus_X]    LKGSW-----DAVFADPLQHPD--HALDPHFTKSVSHRVGPETCDILS-DIGIEVTSV---GE----SQTVSRTGVFDGE-------LHGVLIALDLEIERLVRNHGLPVLCPRSTIGLQ   185
NP_148781.1__triple_gene_block_1_[Cactus_virus_X]    LKGSW-----DAVFADPLQHPD--YALEPHFIKETSHRLGPSTCELIS-SLGILIYPD---SE----DQVVTRKGVFESE-------LFGVVIALDEAIFQLASRHGLKPLCPKATIGLQ   185
YP_054403.1__triple_gene_block_1_[Zygocactus_virus_X]    LKGSW-----DAVFADPLQHPD--FTLEPHFIKETSHRLGRSTCELIS-SLGIFMLPD---AE----DQPVNIQGVFESE-------IHGVVIALDEYIHKLAVRHGLKPFCPKATIGLQ   185
YP_009046883.1__triple_gene_block_1_[Pitaya_virus_X]    LKGSW-----DAVFADPLQHPD--YALEPHFIKETSHRLGPSTCRLIS-ELGILIYPG---AE----DQLVTRKGVFEGE-------LYGTVIALDKPIQELARRHSIEALCPKSTIGLQ   185
YP_002341560.1__triple_gene_block_1_[Schlumbergera_virus_X]    LKGSW-----DAVFADPLQHPD--FAVEPHFIKETSHRLGAQTCQLIS-DLGILIYPG---AE----DQPVSRKGVFEGE-------LYGTVIALDSYIRDLAQRHGVKTLCPKATIGQQ   185
YP_009664729.1__triple_gene_block_1_[Lagenaria_mild_mosaic_virus]    YKEKW-----DVLIADPLQHEN--EPMSPHFIKATSHRFGKNTEDLLT-GLGFNLIGL---KE----SDRVNISHIFGGP-------ILGEVIALEPAARHLLLDHGLKPHCPRSVLGQE   185
NP_044331.1__triple_gene_block_1_[Papaya_mosaic_virus]    ISAKF-----QVLIADPLQYRT--QHLRPHYVNHKSHRLGPETCKLLS-SLGIKVESH---RR---DRDVVTLSGIFGSP-------ILGQAIALDRSASDLLRAHGIQALCPIESIGQE   186
YP_009270631.1__triple_gene_block_1_[Senna_mosaic_virus]    LTSGW-----QVVLADPLQHRG--TFLEPHYIKRVSHRIPKSTEEILE-KAGFQIKAV---KE----AGVCSHTGIFEGP-------LRGKLITLDRTVQDLLVNHGLSPSCPTEVLGQE   185
YP_459945.1__triple_gene_block_1_[Alternanthera_mosaic_virus]    LSGSW-----NVLIADPLQHRS--QALRPHYIKRESHRLGAATCELLT-RVGLPVLSN---KT----EDQVDSQGIFEGP-------LFGTVIALDSTVRALLIKHGISPLCPAEVLGSE   185
YP_009448188.1__triple_gene_block_1_[Babaco_mosaic_virus]    IVGNW-----QVLIADPLQHKP--QALTPHYIKETSHRLGSNTCALLT-SLGIRVNST---RT----DDFVDYSGIFDKP-------LFGEVIALDRPTLDLLESHGILPHCAQKVLGQE   185
YP_010087352.1__triple_gene_block_1_[Cnidium_virus_X]    -PKSA-----SILFGDPLQDPSQAHFLRPHYLKTKSYRIGPEIAQALSQILAIQAFGS----------SKLLRGSAYLED-------PVGLVIAFEDQVCAILRKHQVEHSHPSEIRGVE   181
YP_009091815.1__triple_gene_block_1_[Yam_virus_X]    --DDN-----SILVGDCFQPGHI--SRPAHFLSLKSFRFGSVTAQHLSNILGLPVVGN-SEIQ-----DKIEVLPLFEAE-------LEGAVFCSSEDTCNLLESHQVKHYHKDCVQGAT   184
WEC26234.1__triple_gene_block_1_[Nerine_potexvirus_1]    --PTA-----CFLFADPYQYPPT--NIRAHYTSLITHRFGPTLAAYLT-TLGYHITSSLPAQP-----TELEFLNLWDWT-------PSGTCITHDEHLRNLLVSHSIPTLRPCEAIGLE   188
YP_446993.1__triple_gene_block_1_[Nerine_virus_X]    --ETC-----HFCFADPYQYNID--ALPAHFICNKSYRFGTLTADYLN-SLGYQVRSAG--GS-----DSIRFIDLAHWE-------PEGICITQDSDILNLLTRHGISAYHPCETLGLQ   186
YP_010087333.1__triple_gene_block_1_[Ambrosia_asymptomatic_virus_1]    --EGY-----HCLLADPFQYAQE--PKRAHFIGTHSHRFGRNTAALLT-SLGFTCSSA---RS-----DVVLHTDYWKFR-------PTGQAIAFQAEVCANLRANNLEHTNDTEALGKT   190
QJX15395.1_triple_gene_block_protein_1_[Carnation_latent_virus]    -KLDP-----FVIFGDPNQSRTD-SCLRPHWLSFKTFRFGRSTCPYLN-KLGFKIESE---KE-----DSFKVCKASECD-------IEGQLIVYGQQALTLANWYGLEFLCLEAARGRT   188
NP_620643.1__triple_gene_block_1_[Strawberry_mild_yellow_edge_virus]    -LSEA-----AFLFGDPLQYPGP-AAQVPHFVKLFSHRCGLNSASLIR-ELGIAFEAS---KL-----DSVQHLDPYSSD-------PEGTILAFEPEVQAALASHSLDFLCLDEFRGKQ   185
YP_009389480.1__triple_gene_block_1_[Vanilla_virus_X]    --PDA-----DIIIGDPLQIGAT--HTLAHYSLNRTLRFGRQTAAFVQAVTGIHIEAT---FD-----DILEKGDPFTRD-------PVGQIICGSPVACRLLQWHKVEHLHPAQAVGKT   188
WVS18184.1__triple_gene_block_1_[Hibiscus_virus_X]    --ENF-----DVLIGDPLQGPER-TYPNPHFTKARSFRFGRTTCALLQ-FLGIRAESD---KE-----DQVVIRHPVGAE-------PEGQLIHLCSEGKNRFTQAGYSSLSPWEVLGAT   182
YP_010797204.1__triple_gene_block_1_[Cassava_Colombian_symptomless_virus]    --KAS-----KLLLADPFQYSA--NLSGAHFIKLHTHRFGKNTCALLNRIFNHHFTSD---KE-----DSVVFAGLYQGE-------PIGQLITLEEETFNLCLNHQLTPLRPCELYGRE   182
YP_009357227.1__triple_gene_block_1_[Cassava_virus_X]    --KAS-----KLLIADPLQYSA--KPPLAHFIKATTHRFGQSTCALLRSLLKIEVDSD---RP-----DTVVISRFFEGE-------PEGAIIAFGEEAYHLISAHQLKPFRPCEVYGLS   188
WMX21792.1__triple_gene_block_1_[Adenium_obesum_virus_X]    --QAA-----DFCFADPIQHNIT--EPYPHFTSNFTHRVPEPICNHLR-SLTFDIHST---KA-----GSLTFENIFQGE-------PIGTIIAWEPEIIRLLNSHQAHYLRPCQALGLQ   185
YP_010798309.1__triple_gene_block_1_[Citrus_yellow_mottle_virus]    --PSA-----KYLLADPIQYLGNPNLRKPHYICPVSHRFGSQTAALLS-SLGIEAYSR---KQ-----DIVVSEDIFSFE-------PEGQVIACDPETQALARRHSLDFLRPCESIGRT   181
YP_009124989.1__triple_gene_block_1_[Citrus_yellow_vein_clearing_virus]    --ENV-----EYIFADPIQYLGNPNLRKPHYICASSHRFGHSTAALLT-KLGIETYAH---KE-----DTVRVDNIFQAE-------PDGQIIACDRPTQELAAKHTLDYLRPCESIGLT   181
NP_203554.1__triple_gene_block_1_[Indian_citrus_ringspot_virus]    --PGV-----EYVLADPIQYLGSKDLLKPHYICPTTHRFGHSTAEFLT-SLGIETYAH---KP-----DRLTIANIFKTE-------PHGQVIACDLDTQQLAARHSLDYLRPCQSIGKT   181
YP_224135.1__triple_gene_block_1_[Mint_virus_X]    --AGA-----DVIFADPLQHRGP--TLPAHYTSSITHRFGRATCELLS-KFGITAESN---KE-----DEVFFGWAFADD-------PEGAVICLDAEAQSLASWNGLEHLKPCEALGAT   178
YP_263304.1__triple_gene_block_1_[Lily_virus_X]    SHPPV-----SIVFADPLQHHGE--VRPAHFTCSHTHRFGKSTCSLLG-TLGVYCTSD---KE-----DSVTFAGAYLAE-------PVGTLIALGEDAELVLDNHRVEYFTPCQALGLT   181
YP_001655011.1__triple_gene_block_1_[Phaius_virus_X]    --SGV-----KLLVADPLQHRRG--VLPAHYTGTHTFRFGKSTCDLLK-TLGVVATSD---KE-----DIVSRHPAFNFD-------PEGLIIALGPEAVELLEAHSLPFLRPCQALGLT   179
                                                                                     .          ::                                                                                    .
```

```
NP_619747.1__triple_gene_block_1_[Potato_aucuba_mosaic_virus]              FQTVSCYYQS-----HKLEDSAEERRGLYIAISRAKSAVLLFDL--D------------------------------      232
NP_054026.1_triple_gene_block_1_[Cymbidium_mosaic_virus]                   YSSVSFYCSD-----LR-SCVVLRPLSSLHRSHPRQGQPHHLRF--QCQV---------------------------      229
NP_663725.1__triple_gene_block_1_[Pepino_mosaic_virus]                     FAETTFYCTT-----LA-AAVAENPAKTFISLTRHTHKLTIGEL--NARSNS-------------------------      234
YP_319828.1__triple_gene_block_1_[Alstroemeria_virus_X]                    IPEVSVYTLN-----LK-RAVAEHPHLLFIALTRHSKTLHLFDL--NAGPDTTA-----------------------      234
YP_001960941.1__triple_gene_block_1_[Lettuce_virus_X]                      LPAVAFYCSS-----LQ-DAYDADPAATFIALTRHTTKLEIFEL--DARPDPTA-----------------------      234
NP_040779.1__triple_gene_block_1_[Narcissus_mosaic_virus]                  IPVVSVYITS-----IA-SLSTVDRANLFLSLTRDSKALHIFEF--DAWSHATC-----------------------      233
YP_001715613.1__triple_gene_block_1_[Asparagus_virus_3]                    IRVVSCYVDS-----LE-EALLNHRAPLFLALTRHTAELHIFLF--DARTDAAYELRKC-LQDPRHRGS--------      247
NP_570727.1__triple_gene_block_1_[Scallion_virus_X]                        LSVVSCYLHS-----LE-EALTHYRAPLFLTLTRHTAELHIFLF--DARTDAAHELRNR-LQDFGHRSS--------      247
YP_667845.1__triple_gene_block_1_[Malva_mosaic_virus]                      LETVSVYLSS-----FD-TCLDSFRTDLFLSLTRHTKKLLVFDF--NAWPDSSDEL---------------------      235
YP_009186835.1__triple_gene_block_1_[Plantain_virus_X]                     IPYVSVYTSS-----IG-YALENYRSSLFLALTRHQKQLLIFDL--DARADPTHEL---------------------      235
YP_001718500.1__triple_gene_block_1_[Lolium_latent_virus]                  WPTVTVVFDR----SISPESARPLRCLFYIAATRSSNELNIRTYVPTRTPGL--IKASLPHTSCRLCCRPRDLPSEN      268
NP_569127.1__triple_gene_block_1_[Garlic_virus_A]                          FPEVTLVYHS-----TERL---RSRANFYIAATRALNRLCIIT---DEILPALENSAEP-TTTCG------------      251
NP_659011.1__triple_gene_block_1_[Garlic_virus_E]                          FEEVTLIYHS-----SELS---KNREGFYIAATRALWRLNVIS---DNPPPSLDELCPP-AGSL-------------      244
YP_008855207.1__triple_gene_block_1_[Garlic_virus_D]                       FDEVTLVYHS-----TEFQ---KDRVGFYIAATRAVNRLNLVT---DTHLPQITNR---------------------      237
NP_620649.1__triple_gene_block_1_[Shallot_virus_X]                         FEEVTLVYHS-----SEFE---RNRVGFYIAATRALGRLNLIT---DTTLEIPHELCPT-S----------------      241
NP_569133.1__triple_gene_block_1_[Garlic_virus_C]                          FEELTLVYHS-----SELQ---ANRELFFVAVTRAKRLLNVLT---DSKHRPPTDKARS------------------      244
YP_009110669.1__triple_gene_block_1_[Garlic_virus_B]                       FKEVTLIFHS-----NELP---GNSEAFFIAATRANECLNIIT---DQTPPRLDL----------------------      243
NP_044572.1__triple_gene_block_1_[Garlic_virus_X]                          FKEVTLVYHS-----SELT---ADPNAFFIAATRACGCLGLIT---DKVSHIS------------------------      234
WVN62090.1__triple_gene_block_1_[Rehmannia_allexivirus]                    FEEVTVIFHS-----SEL----ANRVDLFVAFTRARRRLVLLS---DLTWDELRSATRS-K----------------      241
YP_009362669.1__triple_gene_block_1_[Alfalfa_virus_S]                      FQRVALVYHS-----TEL----KNKTAFYVACTRTRHTLCLVS---DQFN-EFCTTT--------------------      238
YP_009328893.1__triple_gene_block_1_[Arachis_pintoi_virus]                 DRSVTLVYHS-----TEL----QERTGFYVAATRALENLTLVS---DEFN-EFHTTP--------------------      237
YP_009389474.1__triple_gene_block_1_[Vanilla_latent_virus]                 FDVVVVVFHS-----SEL----RHRSRLYIACTRAKRHLHLIS---DKFD-EFCTTS--------------------      237
YP_004659201.1__triple_gene_block_1_[Blackberry_virus_E]                   FENTTVVYHS-----SEK----TQTVPFYVCCTRASQSLTLTS---DEFH-EFHTSA--------------------      246
YP_010798341.1__triple_gene_block_1_[Senna_severe_yellow_mosaic_virus]     FEHITVVYHS-----SEL----ADRSGFYIACTRCTSSCTLIS---DEFY-ELRTTS--------------------      240
NP_620716.1__triple_gene_block_1_[White_clover_mosaic_virus]               WPTVTLYLAC-----KISEIPEEERHLLFIGLTRHTESLLILG--PDAFDSSP------------------------      236
NP_042584.1__triple_gene_block_1_[Bamboo_mosaic_virus]                     YDTTTIISST-----PLPTV--KDKVGLYIAFTRHRKACHIRA--PGINPTCDVASSHGPASSSGQTT---------      253
NP_040989.1__triple_gene_block_1_[Foxtail_mosaic_virus]                    FDETTVITTK----TSLEEV--KDRHMVYVALTRHRRTCHLYT--AHFAPSA-------------------------      236
YP_009552763.1__triple_gene_block_1_[Turtle_grass_virus_X]                 FDTTTVVTTQ----PTLSSL--PERHLVYVALTRHRKQCHLRT--SAIATTPGQH----------------------      239
YP_010087745.1__triple_gene_block_1_[Euonymus_yellow_mottle_associated_virus]  YPTVTVLSCE-----ASLKH--VPQHLAYIALTRHRTKLIILS--PNYEPLKSEHASDSST----------------      248
YP_002332930.1__triple_gene_block_1_[Potato_virus_X]                       LKVVTIVSAA-----PIEEI--GQSTAFYNAITRSKGLTYVRA--GT------------------------------      226
YP_009389420.1__triple_gene_block_1_[Euonymus_yellow_vein_virus]           FDCVSVITES-----LIDNV---DFESLYVALSRHCEKLVVLS--ADPRDLLLISDASHSA----------------      237
NP_042696.1__triple_gene_block_1_[Cassava_common_mosaic_virus]             TDVVTVLSTS-----PITEL--NDKVGLYIACTRHTTALHVRA--PPPYPSN-------------------------      231
UTI93305.1__triple_gene_block_1_[Chaenostoma_potexvirus]                   FPEVTVVSAS-----PLDKV--INKSLLYVALSRHTEKLHVLS--PPPHPSS-------------------------      230
NP_620837.1__triple_gene_block_1_[Plantago_asiatica_mosaic_virus]          FDTVTVVSAL-----PLEEV--ADKVGLYISLSRHRSQLHVRS--PPPHPSH-------------------------      230
NP_702989.1_triple_gene_block_1_[Tulip_virus_X]                            FPVVTVVSSL-----PLHEI--VEKHRLYISLSRHTQELHVRC--PPPHPTH-------------------------      230
NP_077080.1__triple_gene_block_1_[Clover_yellow_mosaic_virus]              FPVVTVLSTL-----PLASL--PDKSSVYIALSRHTQELHVRS--PDTPHPAP------------------------      238
YP_002647028.1__triple_gene_block_1_[Allium_virus_X]                       FPITTVISAL-----PLHAV--CDKVGLYIALSRHTSELHVLC--PNPPHPTT------------------------      230
YP_004849315.1__triple_gene_block_1_[Tamus_red_mosaic_virus]               FPVVTVFSAL-----PVSEI--CDKEGAYIALSRHTEELHVRA--PGSFNSTS------------------------      230
YP_224085.1_triple_gene_block_1_[Hydrangea_ringspot_virus]                 FPVVTVVSLS-----PLKQH--PSPQELYIALTRHRKELHVLA--PPPYPTSRPH----------------------      232
YP_002308465.1__triple_gene_block_1_[Hosta_virus_X]                        DDIITVVSEI-----PLSKH--PDKTALYRALTRHTRRLNVLA--PPPYPTP-------------------------      230
YP_054408.1__triple_gene_block_1_[Opuntia_virus_X]                         FPTVTVLSSL-----PLSQV--EDVTGVYISLTRHTKELHVRA--PPETHSTA------------------------      229
QIL68841.1__triple_gene_block_1_[Papaya_virus_X]                           FPKVTVLSSI-----PLTHI--EDVTSVYIALTRHTKELHVRA--PPDPDTTC------------------------      229
NP_148781.1__triple_gene_block_1_[Cactus_virus_X]                          FPVVTVLSSL-----ALEQV--EDSTALYIALTRHTKELHVRC--PTEADATT------------------------      229
YP_054403.1__triple_gene_block_1_[Zygocactus_virus_X]                      FPIVTALSSL-----PLEQV--DDSTGLYIALTRHTQALHVRC--PTEAHAAP------------------------      229
YP_009046883.1__triple_gene_block_1_[Pitaya_virus_X]                       FQQVTVLSSL-----PLESV--EDSTELYIALTRHTREIHVRC--PPSTLTPA------------------------      229
YP_002341560.1__triple_gene_block_1_[Schlumbergera_virus_X]                YPVVTVLSSL-----PLESV--ENSTELYIALTRHTQELHVRC--PNPPHPST------------------------      229
YP_009664729.1__triple_gene_block_1_[Lagenaria_mild_mosaic_virus]          FTEVTVVSFK-----PLSQF--KDSADLYIALTRHSETLHVRT--TGIPHPTP------------------------      229
NP_044331.1__triple_gene_block_1_[Papaya_mosaic_virus]                     YPVVTVVSSE-----PLRNV--RFKDQVYIALSRHTEQLHVLS--PEFPHTTSRPQ---------------------      233
YP_009270631.1__triple_gene_block_1_[Senna_mosaic_virus]                   FPVVTLFSSS-----PLNEV--KAKHLLYIALTRHTKELHVRA--PPFSHTTP------------------------      229
YP_459945.1__triple_gene_block_1_[Alternanthera_mosaic_virus]              FEETTVVSEV-----PLSQV--RFKHALYIALTRHRKSLHVRA--PPLPDTPRRSL---------------------      232
YP_009448188.1__triple_gene_block_1_[Babaco_mosaic_virus]                  FHTTTVLSSV-----PLNLV--REKHLLYIALTRHKHKLHVRA--PALTHTAK------------------------      229
YP_010087352.1__triple_gene_block_1_[Cnidium_virus_X]                      VDTVTLYTST----SVLPKE---HHSDLYVALTRSLAKTLWLTL--DATDARHPTADV-------------------      230
YP_009091815.1__triple_gene_block_1_[Yam_virus_X]                          FNTVTVVAED----RELSSL---TIPERYVLQSRHRQTLRILC--P-------------------------------      221
WEC26234.1__triple_gene_block_1_[Nerine_potexvirus_1]                      FPAVDYVTPS----TPSRAT---VTHLDYIALTRHTLKLRVIT---DDTN---------------------------      228
YP_446993.1__triple_gene_block_1_[Nerine_virus_X]                          FNCVDYLVPK----LPTPAT---ADANTYLALTRHSSRLNVVS---DAADSAT------------------------      229
YP_010087333.1__triple_gene_block_1_[Ambrosia_asymptomatic_virus_1]        YKEVAFYHTDWEELKEEDYP---DRHKLYICLTRHRSKLTLVS--PDHPNYPQVVQQVTPASDAPTTSS--------      254
QJX15395.1_triple_gene_block_protein_1_[Carnation_latent_virus]            FEVCTVLTDY----ESVTDE---IXGDLFVLLTRHRSKCVVLNG--DATLAAT------------------------      232
NP_620643.1__triple_gene_block_1_[Strawberry_mild_yellow_edge_virus]       WPVCTLYVST----KNLCDL---DRPSVYVALTRHYERLLIMSF--DAADTSA------------------------      229
YP_009389480.1__triple_gene_block_1_[Vanilla_virus_X]                      FDSVTVYASS----KDLSDI---PLQHRYVILTRHRTLLLILA--PGH-----------------------------      227
WVS18184.1__triple_gene_block_1_[Hibiscus_virus_X]                         FPTVTLLLEK----RI-KEY---PPHEVYIACSRHCERLVILT--PNAS----------------------------      221
YP_010797204.1__triple_gene_block_1_[Cassava_Colombian_symptomless_virus]  YNEVTVLLSE----TW-THY---PPHLLYLALTRHQTKLLILS--DAVSSTT-------------------------      224
YP_009357227.1__triple_gene_block_1_[Cassava_virus_X]                      FPVVTVAFEQ----EV-DKY---PPHLVYLALSRHTEKLIILS--DALASSS-------------------------      230
WMX21792.1__triple_gene_block_1_[Adenium_obesum_virus_X]                   FDTVTVITLE----PK-------PTHLYYIACTRSLNKLIIKCLSPHRLTTLGN-----------------------      228
YP_010798309.1__triple_gene_block_1_[Citrus_yellow_mottle_virus]           FHTTTVLISD----KLTPET---LTKEIYIGLTRHTSKLIILS--PHAATAST------------------------      225
YP_009124989.1__triple_gene_block_1_[Citrus_yellow_vein_clearing_virus]    FPRTTILISH----ELTADT---LTKEIYIGLTRHSNHLLILT--PDASTTSS------------------------      225
NP_203554.1__triple_gene_block_1_[Indian_citrus_ringspot_virus]            FKDTTILISH----ELNRDT---LTKEIYIALTRHTNSVTILT--PDAPSTSS------------------------      225
YP_224135.1__triple_gene_block_1_[Mint_virus_X]                            FPVVTVISGT----PL-EEA---DAVDRYIALTRHTRLLRILL----------------------------------      213
YP_263304.1__triple_gene_block_1_[Lily_virus_X]                            FPSVTLLTDA----PI-EDQ---PPVSRYIALTRHTDSLLILN----------------------------------      216
YP_001655011.1__triple_gene_block_1_[Phaius_virus_X]                       TSVVTLLTDK----PL-DEQ---DPVDAYISITRHTDKLNILS----------------------------------      214
```

Figure S3. Amino acid alignment of triple gene block 1 (TGB1) sequences of members of the family *Alphaflexiviridae*. Multiple sequence alignment of 66 TGB1 sequences using Clustal Omega.
