# Supplementary material for: Alphaflexiviridae in Focus: Genomic Signatures, Conserved Elements and Viral-Driven Cellular Remodeling
Source: Viruses. 2025 Apr 24;17(5):611. doi: 10.3390/v17050611 (PMC12115993; doi:10.3390/v17050611)
Supplement: Supplementary file 1 [file viruses-17-00611-s001.zip › viruses-3549170-supplementary/Supplementary_files/Figure S4.pdf]

CLUSTAL OMEGA (1.2.4) multiple sequence alignment

```
YP_010797205.1__triple_gene_block_2_[Cassava_Colombian_symptomless_virus]      ---------------MPLVPPPDPNKTYQVGIVAAAICAFCYIFVSDNRSFTGDRENAFPNGGSLSY-CKTANFNRPSGIRFHS-S--SHF-----HVLLLIFSLILAIKLFGQR-----   91
YP_009357228.1__triple_gene_block_2_[Cassava_virus_X]                          ---------------MPLQAPPDPNRTYQLVGVCTAVICICYFLTQDNRGFSGDRENSFPNGGKLSY-CKTAVFHPPNHRDSQA-G--AHI-----FLLVIALT-ALIIFLSRRT-----   90
WVS18185.1__triple_gene_block_2_[Hibiscus_virus_X]                             ---------------MPLEEKRSHHEAIKIAVIGLTLVAFTWAVTRSTLPTVGDPYHNLPHGGHYQDGTKRVDYCGPRKAIHSK-PSEHLW------VLLLILALSAAIYATSRP----A   94
YP_002332931.1__triple_gene_block_2_[Potato_virus_X]                           ----------MSAQGHRLTAPVNSEKVYIVLGLSFALVSITFLLSRNSLPHVGDNIHSLPHGGAYRDGTKAILYNSPNLGSRVS-LHNGKN-----AAFAAVLLLTLLIYGSKYISQRNH   104
QIL68842.1__triple_gene_block_2_[Papaya_virus_X]                               ----------MSGRPLTLTPPADLSKVYLPLAIGIALTLVIYAITRSTLPYVGDSTHALPHGGFYKDGTKTIHYHGIGRNFRTS----AGL-----LPLLLAFSLPLIIYVSSLPFLRSS   101
YP_054409.1__triple_gene_block_2_[Opuntia_virus_X]                             ----------MSGHPLRLTPPPDYSKAVTSLTIGLTLVLAVYAVTRSTLPTVGDSSHSLPHGGFYKDGTKTVKYLGPSD---NH----SKH-----IPLLAVLVIIALIYASSFLSSGRR   98
YP_009046884.1__triple_gene_block_2_[Pitaya_virus_X]                           ----------MSGAPLRLSPPPDYTKVVFPLTLGVGIALIAFTLTRPTLPTVGDSQHSLPHGGWYRDGTKTVAYNSPHY---YS----SGF-----IPFLAVLGLTLLIYVSSLCSGRGA   98
NP_148782.1__triple_gene_block_2_[Cactus_virus_X]                              ----------MSGAPLRLTPPPDHTRTLFPLTLGLGLSLVVFALTRSTLPSVGDSSHSLPHGGWYRDGTKTVFYSGPKK---TA----INW-----SPPIFVLFLTLALYVSYLFESRSR   98
YP_002341561.1__triple_gene_block_2_[Schlumbergera_virus_X]                    ----------MSGAPIHLTPPPDQTKVIFPLTVGLGLTLVVYSLTRSTLPHVGDSTHSLPHGGTYRDGTKSVNYYQPSH---PV----SNH-----LPLIFVLGLIVAIYVSTLFSNRSR   98
YP_054404.1__triple_gene_block_2_[Zygocactus_virus_X]                          ----------MSGVPLRLTPPPDYTKTLLPLTVGLGLTLVIFSLTRSTLPTVGDSSHSLPHGGWYRDGTKLVHYHKPQS---PG----TSW-----TPILLVLGLSAAIYVSHLFSTRSC   98
NP_620717.1__triple_gene_block_2_[White_clover_mosaic_virus]                   ---------------MPLTPPPNPQKTYQIAILALGLVLLAFVLISDHSPKVGDHLHNLPFGGEYKDGTKSIKYFQRPNQHSLS-KT--LAKSHNTTIFLLILGLIVTLHGLHYFNNNRR   102
UTI93306.1__triple_gene_block_2_[Chaenostoma_potexvirus]                       -----------MSSAHHLTPPADFSKPLLALACGISLAIITLSLTRSTLPFSGDPNHSLPFGGLYKDGTKTILFNKPGTST--K-TNHTAW------ALGAIFCLTAIITARALADHT--   98
YP_001655012.1__triple_gene_block_2_[Phaius_virus_X]                           ---------------MPLQPPTDYTRIYSFLVIGVSCVTILYLLTRSTLPHVGDNIHHLPHGGCYRDGTKSIYYGSAKRGS-HP-YPDKLW------AFLAISGLVFAIYWTRPRRAC--   95
NP_042697.1__triple_gene_block_2_[Cassava_common_mosaic_virus]                 -----------MSGHHHLTPPTDYSKAVLAAVIGISSSIFIHTITRSTNPHVGDNIHALPHGGYYRDGTKTVYYGGPGPTAVSA-SAGKFW------AFLCIFTISGFLFFSNRHRT---   99
NP_620838.1__triple_gene_block_2_[Plantago_asiatica_mosaic_virus]              -----------MSGAHHLTPPTDYGKPVLAASIGISLALLVYTATRSTLPHVGDNLHALPHGGRYVDGTKSISYFSPSASK--T-RDPFPF------AFLLILTLSGLILLLSRRRSN--   98
NP_702990.1_triple_gene_block_2_[Tulip_virus_X]                                -----------MSGAHHLTPPTNLGKPVLAVSVGLSLALLAYTATRSTLPHVGDTTHSLPHGGRYVDGTKRIDYFRPHSPN--S-NPTYPF------ILLTILILSGLILLLSRRGPA--   98
YP_010087353.1__triple_gene_block_2_[Cnidium_virus_X]                          ---------------MPLTPDT-QQQTFRTLIACVCVVAAFWALTRSTLPHVGDPSHSLPFGGWYKDGTKSFAFNGQQSGPNSN-HK--SL------ALVLALVIIIVLHALARRSNNNQ   95
NP_042585.1__triple_gene_block_2_[Bamboo_mosaic_virus]                         -----------MDQPLHLARPLDNTRAYLVLAIGVASALFLYTLTRNTLPHTGDNIHHLPHGGRYVDGTKGILYNSPTSSYPSS-SL----------PFSMVIALATTLFLITRNILNPA   98
NP_040990.1__triple_gene_block_2_[Foxtail_mosaic_virus]                        MSLSQGTGAPAISTPLTLRPPPDNTKAILTVAIGIAASLVFFMLTRNNLPHVGDNIHSLPHGGSYIDGTKSINYRPPASRYPSS-NL----------LVSLHQYSPQYSFSSHSHI----   105
YP_009552764.1__triple_gene_block_2_[Turtle_grass_virus_X]                     MSLSHATGSSATSAPLQLQRPPDNTKSYLVLAIGVATAVVIYMLTRSTLPHVGDNIHSLPHGGNYCDGTKSIAYNKPARNFPSS-NL----------LGFAPVLLALALFAVTCPLVTQG   109
YP_010087334.1__triple_gene_block_2_[Ambrosia_asymptomatic_virus_1]            ---------------MPLQPPPNYIPVLVPIAVGIGLALLVVTFRSNQLPNQGDNIHSLPHGGRYRDGTKQVFYNAPQQFQHTH-A-TPAW-----ALLA-TVSLALIITCLSRCRHN--   95
WMX21793.1__triple_gene_block_2_[Adenium_obesum_virus_X]                       ---------------MPLTPPPNHIRELTPFLLGFALVLAIFTCTRNTLPHTGDNIHSLPFGGSYIDGTKRINYLPPKGQGTFF-PS---HAQRAHLPAILVCILPLVIFLLHKATTRNN   101
NP_203555.1__triple_gene_block_2_[Indian_citrus_ringspot_virus]                ---------------MPLQPPPDHTWAVRIIALGLAVTALIFTSTRDTSRHVGDPSHSLPFGGHYRDGSKVIHYNSPRSSKPSN-H-TPYL-------LFAPIGIILLIHALHRLGNSA-   95
YP_010798310.1__triple_gene_block_2_[Citrus_yellow_mottle_virus]               ---------------MPLQPPPDHSWTYRILALGTAFALFTLTLTRDTSRHVGDPSHSLPFGGYYRDGSKVVHYNSPRATRPTS-N-TTLW-------ALLPITLILVIHALHRLNHRD-   95
YP_009124990.1__triple_gene_block_2_[Citrus_yellow_vein_clearing_virus]        ---------------MPLQPPPDHTWAFRLLALGAALALLTFTLNRDTSRHVGDPSHSLPFGGYYRDGSKVVHYNSPRATKPST-P-SFL--------YLAPILLILLIHAVNRFTNPR-   94
NP_044332.1__triple_gene_block_2_[Papaya_mosaic_virus]                         ----------MSSHQNFLTPPPDHSKAILAVAVGVGLAIVLHFSLSYKLPSPGDNIHSLPFGGTYRDGTKSIIYNSPHRGPGQS-G---AL-----PIITVFAIIECTLHVLRKRDNPVR   101
YP_009448189.1__triple_gene_block_2_[Babaco_mosaic_virus]                      ----------MSELPRSLTPPSDNSSAILAVAVGIGLALFTFTLLSYKLPVPGDNIHSLPFGGYYRDGTKSISYNSPRSQASAS-K---SV-----PALLVLAL-VAAIYGITWGDKNRA   100
YP_009664730.1__triple_gene_block_2_[Lagenaria_mild_mosaic_virus]              ----------MSGRQGFLTPPPDHSKTFLALAVGVALALVIHSLLSYRLPTPGDNIHSLPFGGSYIDGTKRIFYNSPRAQSPSS-K---IW-----ALGCIAVC-LSLLHVFKTGDRTRS   100
YP_459946.1__triple_gene_block_2_[Alternanthera_mosaic_virus]                  ----------MSGLPHSLTPPADHSKPVLAAVVGVSLALVINSFLVYRLPSPGDNIHQLPFGGSYRDGTKSIHYNSPRAQSQIS-G---AS-----PFLIILIL-SALIYALSCRGGHHR   100
YP_009270632.1__triple_gene_block_2_[Senna_mosaic_virus]                       ----------MSEHLHSLTPPPDHSRSVLAVAVGLGVALVVFSFRNYSLPTPGDNIHHLPFGGSYRDGTKSVHYSGPRGGQTTF-W---AW-----PIVAIPAL-SLLIYVLSQRSRDKS   100
YP_009389421.1__triple_gene_block_2_[Euonymus_yellow_vein_virus]               ---------------MPLTPPRDYSTVFVVALAAFSLVAFTFTATRSTLPFVGDNIHSLAHGGCYRDGTKSIQYFPPTPVGSAL-K--GGY----LAAAITCIVIPGVLYAVHRSNL---   95
YP_446994.1__triple_gene_block_2_[Nerine_virus_X]                              ---------------MPLTAPPDYTHILPIAIVSIAVALSLYTITRNTLPHTGDNIHHFPHGGRYRDGTKSITYCPPQRNLSPF-NSTGY----SIIPTALAILLPAAIYLSSKCFNSRT   100
NP_620644.1__triple_gene_block_2_[Strawberry_mild_yellow_edge_virus]           ---------------MPLTPPPDHSITYRILAVGLCSCCAIYAATRSTLPHTGDNLHSLPYGGKYSDGTKSICYSGPGPTPDIP-TH---------LPALLVLVLVVAIYASSRLDFSV-   94
YP_010087746.1__triple_gene_block_2_[Euonymus_yellow_mottle_associated_virus]  ---------------MPLTPPPDHSTSLKHLAIGVAIAITIYTASRSNLPHVGDNIHSLPHGGSYRDGTKQILYCSPGSVNSIS-TLAKLFRSTPFVAFLLAAVLPAIIYVSSSFPGLHR   104
YP_002308466.1__triple_gene_block_2_[Hosta_virus_X]                            -----------MSSPHRLTPPPNYTPVLLAVVIGVGLAVVTNQLTRSTLPHVGDNIHSLPHGGNYKDGTKSVIYRGPAPFQRSH-STAPPF--------NAVLLLTFAIWFLSCRTRRAA   100
YP_009389481.1__triple_gene_block_2_[Vanilla_virus_X]                          ---------------MPLIHPPDHTNAIRIAAVFLGVTLTIYALRVNTLPHVGDNIHALPHGGRYQDGTKTIFYNSPFRSQVPS-TL----------PAILALILPAIIYALSRHHRTRV   94
YP_224136.1__triple_gene_block_2_[Mint_virus_X]                                ---------------MPLAPPPDYSKAVLAVAVGLAAALLVHQLTRSTLPHSGDNIHSLPFGGSYVDGTKRIHFGGPSRGS--S---GHLW-----LPALIVAILPGIIWLGRPR-----   90
YP_263305.1__triple_gene_block_2_[Lily_virus_X]                                ---------------MPLTPPPDYTKPFIAVVVGGTLAAFVLLLTRNTLPHTGDNLHSLPHGGTYCDGTKRIRYGGPHRSHVPE-LPAKSW-----ALIT-VVAILIALHFSCLRTHRVH   98
YP_224086.1_triple_gene_block_2_[Hydrangea_ringspot_virus]                     -----------MSSPLRLTPPPDPTKALIPAVVGVSVAAAIFLLTRSTLPHAGDNIHSLPHGGRYRDGTKAVDYCSPHRDP--F-KPTQLH-----AALL-VLALLALIHLSGCLHRRP    100
YP_002647029.1__triple_gene_block_2_[Allium_virus_X]                           ----------MSSAPIHLTPPPDHSKVFISVVVGVSIALCVFLLNKNYLPHVGDNLHSLPHGGTYCDGTKSINYRGPSHHT--S-STTPLW-----AVIA-TLTLPLAIFLLNARRTSHH   101
YP_009091816.1__triple_gene_block_2_[Yam_virus_X]                              ---------------MPLTPPPDHSTTFRIAAATVGLALIFFTLTRSTLPHVGDNIHHLPHGGCYRDGTKTIKYNSPSANA--N-NT-PAW-----ALPA-VLILSALIYATSRLASCSV   95
NP_077081.1__triple_gene_block_2_[Clover_yellow_mosaic_virus]                  ----------MSGAPIHLTPPPDHSKTLFAAVVGVSLAVLVFTLTRSTLPHTGDNIHSLPHGGHYRDGTKVIGYNSPNGTR--S-GPQKTI-----PLVL-AIILPAIIYALSHRRS---   98
YP_004849316.1__triple_gene_block_2_[Tamus_red_mosaic_virus]                   ----------MSGRQVHLTPPVDHSNSVLALVVGVSIALVVFSITRNTSPHVGDNIHSLPHGGCYKDGTKSIYYGKPGNSV--R-QPARGA-----ALVL-VIFLPILIYAIHKFESRRT   101
NP_054027.1_triple_gene_block_2_[Cymbidium_mosaic_virus]                       --------------MPGLVPPPDHSKSLFVLAIGITVVSALFVLKSHTFPIAGDNIHRFPSGGQYKDGTKQINYCPPTHARYPKY-PDYKW-----LAATAAIVIPLC-LYISYHPGNNI   99
YP_001718501.1__triple_gene_block_2_[Lolium_latent_virus]                      -------MSSTSEPTYQLAPPDSLKQVYLTLAAGFAVGLGIFLLRTNTLPHTGDNIHHLPHGGCYRDGTKSIRYNSPGVATSSN-----IF-----LPAVAVLC-ILALLHVPFFQPDRV   102
QJX15396.1_triple_gene_block_protein_2_[Carnation_latent_virus]                ---------------MPLLPPPDHTKAVLAISIGVVVAVVIFALRSNNLPAVGDNVHSLPHGGFYKDGTKTVFYGSKVNAGSVSEHTARIL-----SKPYAAFALICGVLFLSFFNKKKC   100
YP_004659202.1__triple_gene_block_2_[Blackberry_virus_E]                       ---------------MSFTPPPDFSKVYLAAVAGMCATLAIHIQISSRRPHVGDNIHHLPHGGHYQDGNKRIIYAGSHYSNQLQ--TGDH------WAAILVAVLILAILVSERCLRPSN   97
YP_010798342.1__triple_gene_block_2_[Senna_severe_yellow_mosaic_virus]         ---------------MSFAPPPDYTKPLVAAVAGITIALSIFAVSQDHTPHVGDNIHSLPHGGYYQDGNKKIAYAGPNLRPNNS--NTHHF-----WALITIFLVSALIVLSSRLQRNSH   98
YP_009362670.1__triple_gene_block_2_[Alfalfa_virus_S]                          ---------------MSFAPPPDYTKVFLAATIGAALALTTLFATRNTAPHVGDNIHHLPHGGLYQDGNKRIAYAGPGTGAHPR----QHF-----LPAIAVVLLTLGIIISERFKRPTR   96
YP_009328894.1__triple_gene_block_2_[Arachis_pintoi_virus]                     ---------------MSFTPPPDYTKVWIAAAVGGSIAFISFILVQDRTPHVGDNIHHLPHGGSYQDGNKRVLYQGPAHRGANK----SSW-----APAAAVLLLSLAILASELSKRRRR   96
WVN62091.1__triple_gene_block_2_[Rehmannia_allexivirus]                        ---------------MSFAPPPDQSKVYIAAIIGASIGLAIHLLRTNRLPHVGDHSHFLPHGGSYIDGNKAVSYCGPGVPKPTA----HEH-----WPIIAIILLTVAVACSDFVYRRRR   96
YP_009389475.1__triple_gene_block_2_[Vanilla_latent_virus]                     ---------------MSFAPPPDHSKTYTALAIGAGAAVILFVLRQNTLPHVGDNIHHLPHGGCYQDGNKRITYGRL--GNTST----HSW-----HVLLLIFLLSAAIYISS---HRRF   91
YP_009110670.1__triple_gene_block_2_[Garlic_virus_B]                           ---------------MSFTPPPDNSKSILAIAVGAAAALIVFTIRTNHSASVGDNTHRLPYGGQYRDGNKLVNYNGPHTGASPP----SIL-----WPALSAIILTLVIHFRS---GTRR   93
NP_044573.1__triple_gene_block_2_[Garlic_virus_X]                              ---------------MSFTPPPDHTKSFLALATGAAAGLLIYTLRSNQLAPVGDNTHQLPHGGRYRDGNKFVSYNGPNTGASSH----SIW-----WPAITAITLTIVIHFLS---STRR   93
NP_620650.1__triple_gene_block_2_[Shallot_virus_X]                             ---------------MSFAPPPDYSKIYLALGCGLGLGFVVYASRVNHLPHVGDNTHNLPHGGQYCDGNKRVLYSGPKSGSSPT----NNL-----WPFITVIALTLAILLTS---CPRR   93
NP_569134.1__triple_gene_block_2_[Garlic_virus_C]                              ---------------MSFTPPPDYNKVYLTLAIGAAVGILVHTLRSNHLTHVGDNTHHLPHGGRYCDGNKRIHYNGPQAGSTHV----SSV-----LPFFAAITLTLVIHFIS---CRRR   93
YP_008855208.1__triple_gene_block_2_[Garlic_virus_D]                           ---------------MSFAPPPDYSKVYITLAGGLATGILIYTLRSNHLPHVGDNTHNLPHGGRYCDGNKQIHYYKPNSGGQHG----GSF-----VPLFFVFLLSLAILLLS---CPRR   93
NP_569128.1__triple_gene_block_2_[Garlic_virus_A]                              ---------------MSFAPPPDYSKAYITLAGGAALGILVYTLRSDHLPHVGDNTHHLPHGGRYCDGNKQIHYFKPNSGSPSG----GSY-----IPPLIIFTLTLAILLLS---RPRR   93
NP_659012.1__triple_gene_block_2_[Garlic_virus_E]                              ---------------MSFAPPPDHSKVYITLAGGAALGIFVYTLRSNQLPHVGDNTHSLPHGGRYCDGNKQIHYFKPNAGGQSG----SSH-----LPLYAVFFLTLAILLLS---RPRR   93
NP_663726.1__triple_gene_block_2_[Pepino_mosaic_virus]                         --------------MPGLTPRADLTDTYKIIAIAFLLSACIYFQNSHYQPVAGDNLHRLPFGGQYQDGTKKISYFPQQQSYFHS-GNKLNV-----LILIFILTLGI--VLTNKFSFSFS   98
NP_619748.1__triple_gene_block_2_[Potato_aucuba_mosaic_virus]                   --------------MPGLTPPKDYTASVKVLAIAVLRFATIFAIRANHQPQVGDSSHSLPFGGHYKDGTKEVLYYPQNSKVALK-GFDNKI-----VA--FIVCLSILIYVS----LPRL   94
YP_319829.1__triple_gene_block_2_[Alstroemeria_virus_X]                        --------------MPGLTPPPNYENVYKLVALGFICVGIILATKSNHNHHTGDQQHSLPFGGIYRDGTKTVSYFRPSDPITH----SNKW-----LAFSSVITISLIIWLCSKFSPGDR   97
YP_001960942.1__triple_gene_block_2_[Lettuce_virus_X]                          --------------MPGLTPPPNHEHTFQIIAIGFLCCGIIYALRTNHAPHTGDQQHSLPFGGHYQDGTKRVIYNSPSYPNVR----CSKL-----LALAAILGLSAFILGN-QWYSRRS   96
YP_009186836.1__triple_gene_block_2_[Plantain_virus_X]                         --------------MPGLTPPTNFEQVWKIAAIGLLTCGSIYALRANHQPHVGDNLHALPHGGLYRDGTKQITYCSPQKTHAY----NHKW-----TAAAAIAILSLLIYAQSRFSLGNR   97
NP_040780.1__triple_gene_block_2_[Narcissus_mosaic_virus]                      --------------MPGLTPPVNYEQVYKVLAIGFLLCASIYCLRSNHLPHVGDNIHSLPHGGNYADGTKRVQYFRPHSSTST----NHKY-----TALCAVLTLSLLIFAQTRLAAGNR   97
YP_001715614.1__triple_gene_block_2_[Asparagus_virus_3]                        --------------MPGLTPPTNYESVFKILAIGALSCLSIYSLRTNHLPHTGDNIHHLPHGGNYADGTKRVQYFRPSAPVHG----SSKF-----TAACAILFLTLLILAQSQWPARAV   97
YP_667846.1__triple_gene_block_2_[Malva_mosaic_virus]                          --------------MPGLTAPTNYEQVYKILAIGVLSCLSIYALRASHLPHVGDNLHHLPHGGNYVDGTKRISYFKPHTSHGA----NYKW-----SAACAIGFLSLLIFAQTRFNSRPV   97
                                                                                                :                                  **  : :  **      *   :
```

```
YP_010797205.1__triple_gene_block_2_[Cassava_Colombian_symptomless_virus]      --------SVHCPTCRG-----------------------------      100
YP_009357228.1__triple_gene_block_2_[Cassava_virus_X]      --------PHSCPQCRG-----------------------------      99
WVS18185.1__triple_gene_block_2_[Hibiscus_virus_X]      --------A--APLHPSGHLHFVPPPPAREGMRAHDHGGVPDFNQL      130
YP_002332931.1__triple_gene_block_2_[Potato_virus_X]      --------TCACGNNHSSH---------------------------      115
QIL68842.1__triple_gene_block_2_[Papaya_virus_X]      --------TRSCPHCSQQHPH-------------------------      114
YP_054409.1__triple_gene_block_2_[Opuntia_virus_X]      --------TGTCSSCGTAHG--------------------------      110
YP_009046884.1__triple_gene_block_2_[Pitaya_virus_X]      --------TRSCPHCCGQH---------------------------      109
NP_148782.1__triple_gene_block_2_[Cactus_virus_X]      --------AGSCSHCGSNHT--------------------------      110
YP_002341561.1__triple_gene_block_2_[Schlumbergera_virus_X]      --------GRHCNHCGAKHA--------------------------      110
YP_054404.1__triple_gene_block_2_[Zygocactus_virus_X]      --------ARHCPHCGAKHTESA-----------------------      113
NP_620717.1__triple_gene_block_2_[White_clover_mosaic_virus]      V-S----SSLHCVLCQNKH---------------------------      116
UTI93306.1__triple_gene_block_2_[Chaenostoma_potexvirus]      --------HRVCRCCSKLH---------------------------      109
YP_001655012.1__triple_gene_block_2_[Phaius_virus_X]      --------PCTCPRCVEPSAVPNNN---------------------      112
NP_042697.1__triple_gene_block_2_[Cassava_common_mosaic_virus]      --------NSSCSCRPNLGAN-------------------------      112
NP_620838.1__triple_gene_block_2_[Plantago_asiatica_mosaic_virus]      --------PHSCPSCGTPHA--------------------------      110
NP_702990.1_triple_gene_block_2_[Tulip_virus_X]      --------PARCPCCQRPH---------------------------      109
YP_010087353.1__triple_gene_block_2_[Cnidium_virus_X]      C-A-------------RFVN--------------------------      101
NP_042585.1_triple_gene_block_2_[Bamboo_mosaic_virus]      P-TTPRIYAPLCLHCHRNHPPC------------------------      119
NP_040990.1__triple_gene_block_2_[Foxtail_mosaic_virus]      ----------------------------------------------      105
YP_009552764.1__triple_gene_block_2_[Turtle_grass_virus_X]      P-ASYTRRISICHQCGTAPSNSSSPHS-------------------      135
YP_010087334.1__triple_gene_block_2_[Ambrosia_asymptomatic_virus_1]      -----------PVCCTSGPR-ARVYHIH------------------      111
WMX21793.1__triple_gene_block_2_[Adenium_obesum_virus_X]      P-G----H--TCTTCSSQTL--------------------------      114
NP_203555.1__triple_gene_block_2_[Indian_citrus_ringspot_virus]      -------HICRCTHCMPHSQ-T------------------------      109
YP_010798310.1__triple_gene_block_2_[Citrus_yellow_mottle_virus]      -------HSCRCNHCISTSQ-T------------------------      109
YP_009124990.1__triple_gene_block_2_[Citrus_yellow_vein_clearing_virus]      -------HSCSCTHCQPIPR-T------------------------      108
NP_044332.1__triple_gene_block_2_[Papaya_mosaic_virus]      P-Q-----HSDCPNCS------------------------------      111
YP_009448189.1__triple_gene_block_2_[Babaco_mosaic_virus]      R-G-----VRACPCHLHSPQ--------------------------      114
YP_009664730.1__triple_gene_block_2_[Lagenaria_mild_mosaic_virus]      S-S-----SCNCTCCT------------------------------      110
YP_459946.1__triple_gene_block_2_[Alternanthera_mosaic_virus]      A-R-----LHRCPCCS------------------------------      110
YP_009270632.1__triple_gene_block_2_[Senna_mosaic_virus]      G-G-----VHICPCCSYMVE-LRH----------------------      117
YP_009389421.1__triple_gene_block_2_[Euonymus_yellow_vein_virus]      H-C----KPVPCPNCRTHVA-SPQ----------------------      113
YP_446994.1__triple_gene_block_2_[Nerine_virus_X]      H-P----H--SCSHCQPNSA-TMRGTS-------------------      119
NP_620644.1__triple_gene_block_2_[Strawberry_mild_yellow_edge_virus]      --N----YRCSCRVHNRSGQ--------------------------      108
YP_010087746.1__triple_gene_block_2_[Euonymus_yellow_mottle_associated_virus]      S--------CNHPSHTHH----------------------------      114
YP_002308466.1__triple_gene_block_2_[Hosta_virus_X]      I-G----I-HVCHTCSQTRE-QQ-----------------------      116
YP_009389481.1__triple_gene_block_2_[Vanilla_virus_X]      V-T----AWADTHHHHHHPA--------------------------      109
YP_224136.1__triple_gene_block_2_[Mint_virus_X]      A-A----HVCRCPLCAAARP-P------------------------      106
YP_263305.1__triple_gene_block_2_[Lily_virus_X]      R-C----VLCHT---TSG----------------------------      108
YP_224086.1_triple_gene_block_2_[Hydrangea_ringspot_virus]      P-D----IPCSCPAHPPPP---------------------------      114
YP_002647029.1__triple_gene_block_2_[Allium_virus_X]      S-T----LRCGHAACASGLD-STNP---------------------      120
YP_009091816.1__triple_gene_block_2_[Yam_virus_X]      ---------TSTRYCIRSDN-NL-----------------------      108
NP_077081.1__triple_gene_block_2_[Clover_yellow_mosaic_virus]      --P----HPCSNPLCPALHN-QG-----------------------      114
YP_004849316.1__triple_gene_block_2_[Tamus_red_mosaic_virus]      R-V----SACGHIACPSGGA-TQH----------------------      119
NP_054027.1_triple_gene_block_2_[Cymbidium_mosaic_virus]      R--------RICPCCNTYHHP-------------------------      112
YP_001718501.1__triple_gene_block_2_[Lolium_latent_virus]      R--------RRCCRFYWCADPHH----PTV----------------      120
QJX15396.1_triple_gene_block_protein_2_[Carnation_latent_virus]      N--------CTCDRCKRLA---------------------------      111
YP_004659202.1__triple_gene_block_2_[Blackberry_virus_E]      R---------SCPHHH------------------------------      104
YP_010798342.1__triple_gene_block_2_[Senna_severe_yellow_mosaic_virus]      ---------PVCVH--------------------------------      103
YP_009362670.1__triple_gene_block_2_[Alfalfa_virus_S]      S--------CRC----------------------------------      100
YP_009328894.1__triple_gene_block_2_[Arachis_pintoi_virus]      V--------HHCVTCTTR----------------------------      106
WVN62091.1__triple_gene_block_2_[Rehmannia_allexivirus]      P---------TCHHHSH-----------------------------      104
YP_009389475.1__triple_gene_block_2_[Vanilla_latent_virus]      R---------VELHCAHCHR--------------------------      102
YP_009110670.1__triple_gene_block_2_[Garlic_virus_B]      R---------ICIHCSESTSAHQ----S------------------      108
NP_044573.1__triple_gene_block_2_[Garlic_virus_X]      R---------VCVRCSESTLPH------------------------      106
NP_620650.1__triple_gene_block_2_[Shallot_virus_X]      R---------VCIRCSQHH---------------------------      103
NP_569134.1__triple_gene_block_2_[Garlic_virus_C]      R---------VCIRCSEPH---------------------------      103
YP_008855208.1__triple_gene_block_2_[Garlic_virus_D]      R---------LCVRCSEPH---------------------------      103
NP_569128.1__triple_gene_block_2_[Garlic_virus_A]      R---------ICIRCSESH---------------------------      103
NP_659012.1__triple_gene_block_2_[Garlic_virus_E]      R---------LCVRCSESH---------------------------      103
NP_663726.1__triple_gene_block_2_[Pepino_mosaic_virus]      RTT----HQHSCYNTHSAT----NNTQPLSGHH-------------      123
NP_619748.1__triple_gene_block_2_[Potato_aucuba_mosaic_virus]      F-A----SNNVCRTCNSSTVAQ------------------------      111
YP_319829.1__triple_gene_block_2_[Alstroemeria_virus_X]      R-P----LPPVCSHCTSH----------------------------      110
YP_001960942.1__triple_gene_block_2_[Lettuce_virus_X]      V-T----RIHTCVHCSNVPPSL------------------------      113
YP_009186836.1__triple_gene_block_2_[Plantain_virus_X]      P-D----PVRVCRHCPQAGNDVHGHHHG------------------      120
NP_040780.1__triple_gene_block_2_[Narcissus_mosaic_virus]      I-T----SVSICHHCSSQGSLSGGNHGRVSGHSE-----LPTT---      130
YP_001715614.1__triple_gene_block_2_[Asparagus_virus_3]      R-C----SVRVCGHCHPDSTMPSNSDR-------------------      119
YP_667846.1__triple_gene_block_2_[Malva_mosaic_virus]      S-T----SVRVCAHCTSNSSVPSDNHR-------------------      119
```

Figure S4. Amino acid alignment of triple gene block 2 (TGB2) sequences of members of the family *Alphaflexiviridae*. Multiple sequence alignment of 66 TGB2 sequences using Clustal Omega.
