# Supplementary material for: Alphaflexiviridae in Focus: Genomic Signatures, Conserved Elements and Viral-Driven Cellular Remodeling
Source: Viruses. 2025 Apr 24;17(5):611. doi: 10.3390/v17050611 (PMC12115993; doi:10.3390/v17050611)
Supplement: Supplementary file 1 [file viruses-17-00611-s001.zip › viruses-3549170-supplementary/Supplementary_files/Figure S5.pdf]

CLUSTAL OMEGA (1.2.4) multiple sequence alignment

```
NP_619749.1_triple_gene_block_3_[Potato_aucuba_mosaic_virus]                       ------------------------------------------------MY-------RYLDC-LLV----I-MCVVLA--IVLLWPNNYHPCVVNNSGAEIHIHNCAEPNKIISSIQSHL   57
NP_663727.1__triple_gene_block_3_[Pepino_mosaic_virus]                             -----------------------------------------MSSYLSSFSRWVLS--SPINL-VLALVVLL-TSILAI--THIQQPTIHNHCQAIIDSAAIVITNCENTPEVLKAINFSP   73
YP_001960943.1__triple_gene_block_3_[Lettuce_virus_X]                              ------------------------M------SDAPSSWPWRRSSGCQPLS-------WAING-TLG----A-LLLVFI--LVCIAPTYHHPCRVVISGHTITITDCPNAAEILRGLDVAP   75
YP_319830.1__triple_gene_block_3_[Alstroemeria_virus_X]                            ----------MAPKLSLTFALLTLS------PTLINGWPSHQSSPSASSF------GYVLNF-LLV----I-GGLYLL--YALTAPPTKAGCIVRVTGESVTIDNCPDTALIMEKFNLAP   90
YP_009186837.1__triple_gene_block_3_[Plantain_virus_X]                             ------------------------M------PTTTSG----PQQPPSPFFLFLFT--HKVDF-LLG----I-ALILFV--YAVTVPRQATTCTVTITGEAIRVVGCPDPGSVFTNLNIAP   76
YP_001715615.1__triple_gene_block_3_[Asparagus_virus_3]                            -------------------------------MAVQNS------PPPAPFFSSLCSSLLSLNG-LLV----L-SVVLCA--YAATVTP-TPPCQVIVTGEAVRIIGCAEPSHILANLNLAP   74
YP_667847.1__triple_gene_block_3_[Malva_mosaic_virus]                              ------------------------M------VLTTSG----VPPAPSGFFHFLSS--HKLDL-ILV----L-SVLLFV--YALIAPP-TPVCQVIITGESVKVLNCPAPDKIIASIQLAP   75
NP_040781.1__triple_gene_block_3_[Narcissus_mosaic_virus]                          --------MQTAPREYSTSGPTAVL------APTTNT----QHYAPYSLYRFLSS--HKLDL-LLG----I-ALLVFL--YVITAAP-KEVCQVVITGESVVIRNCQQPDRILANLNLSP   91
NP_620645.1__triple_gene_block_3_[Strawberry_mild_yellow_edge_virus]               ----------------------------------------------------MRV--LD--L------ILALITAAVV--GYTIALVSNSGCYVHFDGRSATTT-CPPGPWVESIANGLY   55
YP_002647030.1__triple_gene_block_3_[Allium_virus_X]                               ------------------------------------------------MRA-------E--L-LIT--LLSAAVTLLV--LQVWTQPTPNSCYIEVNGHTAYTN-CPPSPHLAQVVAHLK   57
NP_077082.1__triple_gene_block_3_[Clover_yellow_mosaic_virus]                      ------------------------------------------------MHL-------A--I--VG--ALTLVLTLFV---LHY-TTKDDRCYILINGHSAFTN-CPASPDLAKVISQLK   54
YP_004849317.1__triple_gene_block_3_[Tamus_red_mosaic_virus]                       -------------------------------------------MPFTSLNL-------A--V--LV--SA-LVVTLLV--LQVV-QRNTDNCIIVITGHHAETN-CPPSLELAEVLKYLK   59
YP_002308467.1__triple_gene_block_3_[Hosta_virus_X]                                -------------------------------------------MQSFCSHLRSGS--FP--V-VLGALLLAFTCATLV--LRL-GNNNSNNCLIYVDGARAFLE-GNCAGISAEVVAALR   68
NP_042698.1__triple_gene_block_3_[Cassava_common_mosaic_virus]                     ----ME---GPGPQLFP---------LQP--ESSGRSSASLPSQAFFSSQIGIGL---T--L-VV-----AAVLT-WA--LIS-LQSSKGGCLIIIDGAKALLQ-GDCSYISPEILANLH   86
YP_224087.1_triple_gene_block_3_[Hydrangea_ringspot_virus]                         --------------------------MPP--SSSSHSWPSSTSLAVFTA---------------------ALVLTFLA--LAL-LTPPPPKCFVRVSGAEATLS-GDCSTLSPEALRHLK   67
YP_001718502.1__triple_gene_block_3_[Lolium_latent_virus]                          ---------------------------------------------------MSLS--FSLIVFAVGVAV---SIGVLTL----TTQQSSSYCLILVDGAKAVVEGCHLRQDIPAILSELK   60
YP_001655013.1__triple_gene_block_3_[Phaius_virus_X]                               ----MEAATGMARSPYTTAQQR------------EGATP-TLT--SSGLSWLFQG--SSLP--FIGLGL---AALALAL---ALGVSNPRQCLITIDGAGAILQGCGEIQDLPAVINALH   91
YP_054410.1__triple_gene_block_3_[Opuntia_virus_X]                                 ---------------------------------------------------MLVL--SF----LVGAAL---GLALLAA---LHTG-ENKGCLIQFNGHSTIVSNCQDVKDLAAVINAIN   56
QIL68843.1_triple_gene_block_3_[Papaya_virus_X]                                    ---------------------------------------------------MFLL--YL----FLGLLL---GVALIAV---SNTLTNCGTCLIVFNGHSTTITGCERVPDLDKVVNALN   57
YP_009046885.1__triple_gene_block_3_[Pitaya_virus_X]                               ---------------------------------------------------MFLV--YV----LVGVLL---GVALIAV---VNT--NNPQCTIVFTGHSTTIQGCERLTNLHDIIAAVN   55
YP_002341562.1__triple_gene_block_3_[Schlumbergera_virus_X]                        ---------------------------------------------------MFLL--YS----LIGVVV---GIAIIVV---LNTL-NSPSCTIILNGHSTIIQGCTQLSNLDSVIEALN   56
NP_148783.1__triple_gene_block_3_[Cactus_virus_X]                                  ---------------------------------------------------MFLT--YL----SLGVVL---GLAVIAG---LTTL-NTPSCLIVLSGDQTVVKGCEHIGNLADVITALN   56
YP_054405.1__triple_gene_block_3_[Zygocactus_virus_X]                              ---------------------------------------------------MFLT--YL----VLGAVL---GIALTAV---LNTL-NQPNCVLVFTGERTVVSGCEHLPHLSEVINSLN   56
WVN62092.1__triple_gene_block_3_[Rehmannia_allexivirus]                            -------------------MNT------------GQSSRSY------YSQWPL-----L----VLTLCT---AAAVLLATTTL--TNPPPGCRFVITGSTVEALQCNPAEVA-EAVRSFA   68
YP_009362671.1__triple_gene_block_3_[Alfalfa_virus_S]                              ----MQAPELVH----THASTS------------CQQSPWY------SSPWAL-----L----LVS---GLSALLVLAAANYFNSLPGSHSCLLTITGHSVSVSGCENHDVP-AIIQSFA   81
YP_010798343.1__triple_gene_block_3_[Senna_severe_yellow_mosaic_virus]             ----MQAPICDR----TTA-TH------------TTSGPSS------QSSWSP-----L----LLSLVVACSAILTLFVCTKL--SRVTPGCQIIITGHSVSITGCENTPVA-DIVRAFS   81
QJX15397.1_triple_gene_block_protein_3_[Carnation_latent_virus]                    ---------------------------------------------MQ-----ASG--LI----LVALXSAAVSYL-----ALLHLSSSSSSCVVVVTGESFRISGCDFTEEFIGFAKTLR   59
YP_010087335.1__triple_gene_block_3_[Ambrosia_asymptomatic_virus_1]                ----------------------------------------MQP--PLGLSWPQYL--SL----SLSLAFLAAAIT-----LFVALPGQEPGCTISIDGTGIYIKNCGQHAEFIQSVSKLK   67
YP_010087747.1__triple_gene_block_3_[Euonymus_yellow_mottle_associated_virus]      ---------------------------------------------MLAPH--FQV--FI----AAAITL---LIL-----II----SSRDPCVITITGESVRIVNCPLSEGLISQLSSLK   55
YP_009389482.1__triple_gene_block_3_[Vanilla_virus_X]                              ---------------------------------------------MHSLDTIVLG--LL----LLGLTL---TII-----TI----IQPKPCTLLIDGSQVLITNCKLDPSILEALASIK   57
YP_009091817.1__triple_gene_block_3_[Yam_virus_X]                                  ---------------------------------------------MQPLDWLVAL--LL----LLGTVF---AAI-----TI----YKEPPCTLVLDGASLTITGCKLTPELISQISGLS   57
WMX21794.1__triple_gene_block_3_[Adenium_obesum_virus_X]                           ------------------MHNE------------HTSPPSLFA--FFHLSFSFYI--KL----LLGITL---GIL-----VLLALPRPYEPCTIIITGERVTAHGCPVTQELANILSNRE   74
WVS18186.1__triple_gene_block_3_[Hibiscus_virus_X]                                 -------------------------------------------------MPPVDL--LL----LLCIPL---AIY-----TLYLLLQPERACVLTITGESLTLTNCDLSPELLKALPTQP   57
NP_042586.1__triple_gene_block_3_[Bamboo_mosaic_virus]                             -----------------------------------------------MLNTDTL----------CIILF---ILILGIL-YNILQQHLPPPCEIIINGHTISIRGNCY--HTTSS-----   52
NP_040991.1__triple_gene_block_3_[Foxtail_mosaic_virus]                            ------------------------------------------------MHESHL----------VVIL----ALLLIAL-WCLSTRPVQPSCHVEINGHSIIVTGNCW--HSTQRPH---   52
YP_009552765.1__triple_gene_block_3_[Turtle_grass_virus_X]                         ------------------------------------------------MRDGAL----------QLILA---ALLGLIL-YLATRQPARPNCYLEINGHSIVVSGDCW--HTSAKSTNR-   55
UTI93307.1_triple_gene_block_3_[Chaenostoma_potexvirus]                            --------------------------MEPPPRPITRPGPWEQS--FASLLSSPL----------ALLLI---TLTVCAV-VVLSCTESRNGCRITITGHSTHIEGDCP--ITVELIKAAH   76
NP_620839.1__triple_gene_block_3_[Plantago_asiatica_mosaic_virus]                  MHYPTEADTSTGPNPSATSAPVRPRHVTPSLSPSSSSSPSPDS--FYYFLAAAV--------------------ILTAA-LAAALLTPNPGCTIVITGHTTIIQGSCP--IPPQLVLAAH   95
NP_702991.1__triple_gene_block_3_[Tulip_virus_X]                                   ----------MEPNASTTSDHTPRTPIRPIPSSSSQSSSYPDS--FYFCLAAVL--------------------LLLAV-LAANALTNHHPCTVHINGHSTTLTGNCV--ITPELLTSLH   85
NP_044333.1__triple_gene_block_3_[Papaya_mosaic_virus]                             ------------------------------------------------MFSGKEI--------TLFALS---TLIALIV-LNYMSATPNPVCLIELTGHSAVLRGNNCESLTSGVIEALS   60
YP_009448190.1__triple_gene_block_3_[Babaco_mosaic_virus]                          ------------------------------------------------MASLGEI--------KIALAV---FVLALAI-FTLLNSNKRETCLVEVSATLSRVSGQDCRHITPEIIQALG   60
YP_009664731.1__triple_gene_block_3_[Lagenaria_mild_mosaic_virus]                  ------------------------------------------------MFSKQEI--------ALGVLA---AVIALAA-LNWHPRASTPICQIVLDGSSSKLVGDCS-NIGADVVRALG   59
YP_459947.1__triple_gene_block_3_[Alternanthera_mosaic_virus]                      ------------------------------------------------MPYLVEA--------AITVLA---CIGVLAA-L---RPG-SHPCTILLTGHSATISGDCG-PVAPETIRALG   55
YP_009270633.1__triple_gene_block_3_[Senna_mosaic_virus]                           ------------------------------------------------MYYLREA--------AISLGV---CIFALAA-LTWWSSD-TKECKIILTATSSTVSGRCEGIVSPDLIHALG   59
YP_010087354.1__triple_gene_block_3_[Cnidium_virus_X]                              ------------------------------------------------MHWQDA----------VIIIS---VLVLLIS-LYYGSHSRRDGCFIHIDGATASASNCSPEQLSAISHGFLK   58
YP_002332932.1__triple_gene_block_3_[Potato_virus_X]                               ----------------------------------------------MEVNTYLN---------AIILVL---VVTI-IA-VISTSLVRTEPCVIKITGESITVLACKLDAETIKAIADLK   60
NP_620718.1__triple_gene_block_3_[White_clover_mosaic_virus]                       ---------------------------------------------------MDF---------TTLIII---GVYLLVF-IVYFAKINTSVCTISISGASIEISGCDNPT-LFEILPKLR   55
NP_054028.1__triple_gene_block_3_[Cymbidium_mosaic_virus]                          ----------------MLG-TRNILT--------TSGLPLPPP--SSSLSAYIF---------PTILAI---IFAVFAL-VAIHITTPEPFCTIHIDGASITITNCPDPAAILNKVA-IG   79
YP_446995.1__triple_gene_block_3_[Nerine_virus_X]                                  ----MEAATGTEPSLSPIARHNAIYHRLTA-PV-TASSPQHSP--SYSQRQFIL---------ALSVSI---AALILTL-AV-IASQTPPPCVVHLDGNSAHIENCSNQPELPELVNAAS   98
YP_224137.1__triple_gene_block_3_[Mint_virus_X]                                    --------MSTAPSAFTSGGHHA-----AA-PAIFGCLPSL-L--PYCRASFGW----------AGL-----ALLTFAA-ALFALRPAPPDCTIVVTGHSLTVSGCVGHRGAELLLQGLS   87
YP_263306.1__triple_gene_block_3_[Lily_virus_X]                                    ------------------------------------------------MYSSPY----------ILAAY---ALIAFTA-VCYVTQPAAETCYIEVTGHNAVIRGCVNVPHLPEVVPALA   58
YP_009389422.1__triple_gene_block_3_[Euonymus_yellow_vein_virus]                   ------------------------------------------------MRSIEA----------ICIVS-----LSLAL-IVALTWQAPNRCFVEISGAHVRFVGCAESPQLWEAVKAVK   56
YP_009124991.1__triple_gene_block_3_[Citrus_yellow_vein_clearing_virus]            ------------------------------------------------MQSIDL----------LILAI-----LVLAL-IA-SLFPAPEPCTIVVSGASASVTNCPNPEQLAELVRALK   55
YP_010798311.1__triple_gene_block_3_[Citrus_yellow_mottle_virus]                   ------------------------------------------------MHSIDL----------IIGII-----LAGAI-IV-FLLPKPEPCVITVSGASATVSNCPNPELLTDLVRALK   55
NP_203556.1__triple_gene_block_3_[Indian_citrus_ringspot_virus]                    ------------------------------------------------MHYIDW----------VILLT-----FAAAL-IV-CLTPKPEPCIITVSGASATVSNCPNPELLTDLVKALK   55
                                                                                                                                                                              *     .
```

```
NP_619749.1_triple_gene_block_3_[Potato_aucuba_mosaic_virus]                     GT--GLSFHLKVLINIVN-----------------------------------------    73
NP_663727.1__triple_gene_block_3_[Pepino_mosaic_virus]                           WN--GLSFPKFEN----------------------------------------------    84
YP_001960943.1__triple_gene_block_3_[Lettuce_virus_X]                            WN--GVKFPQL------------------------------------------------    84
YP_319830.1__triple_gene_block_3_[Alstroemeria_virus_X]                          WN--GVKFPKE------------------------------------------------    99
YP_009186837.1__triple_gene_block_3_[Plantain_virus_X]                           WN--GVKFPILDFEN--------------------------------------------    89
YP_001715615.1__triple_gene_block_3_[Asparagus_virus_3]                          WN--GVKFPILQV----------------------------------------------    85
YP_667847.1__triple_gene_block_3_[Malva_mosaic_virus]                            WN--GVKFPIL------------------------------------------------    84
NP_040781.1__triple_gene_block_3_[Narcissus_mosaic_virus]                        WN--GVKFPLL------------------------------------------------    100
NP_620645.1__triple_gene_block_3_[Strawberry_mild_yellow_edge_virus]             TAGLARPHPEPECERRQS-----------------SW----------------------    75
YP_002647030.1__triple_gene_block_3_[Allium_virus_X]                             PHSHAVKFPTGLKQNDRQ-----------------DCS----HQCPL------------    83
NP_077082.1__triple_gene_block_3_[Clover_yellow_mosaic_virus]                    PHNHG------------------------------------------------------    59
YP_004849317.1__triple_gene_block_3_[Tamus_red_mosaic_virus]                     PHHHG------------------------------------------------------    64
YP_002308467.1__triple_gene_block_3_[Hosta_virus_X]                              PHSHAG-----------------------------------------------------    74
NP_042698.1__triple_gene_block_3_[Cassava_common_mosaic_virus]                   PYGLSLPKSRN------------------------------------------------    97
YP_224087.1_triple_gene_block_3_[Hydrangea_ringspot_virus]                       PHSHRC-----------------------------------------------------    73
YP_001718502.1__triple_gene_block_3_[Lolium_latent_virus]                        PASSP--FNPL------------------------FCS---------------------    72
YP_001655013.1__triple_gene_block_3_[Phaius_virus_X]                             YAKGL-SYPNI-----------------------RNCEYGYHEHCEQVRSCEHASLDGP    126
YP_054410.1__triple_gene_block_3_[Opuntia_virus_X]                               DRLS---FSN-------------------------------------------------    63
QIL68843.1__triple_gene_block_3_[Papaya_virus_X]                                 NRLS---FSSV------------------------------------------------    65
YP_009046885.1__triple_gene_block_3_[Pitaya_virus_X]                             NRLS---FSSS---------------------------------CEN------------    66
YP_002341562.1__triple_gene_block_3_[Schlumbergera_virus_X]                      HRLS-------------------------------------------------------    60
NP_148783.1__triple_gene_block_3_[Cactus_virus_X]                                NRLS---FNKL------------------------------------------------    64
YP_054405.1__triple_gene_block_3_[Zygocactus_virus_X]                            NRLS---FN--------------------------------------------------    62
WVN62092.1__triple_gene_block_3_[Rehmannia_allexivirus]                          WSSHDFRDGRLL----------------QQLPKHPNRLAPKHPKCHL------------    99
YP_009362671.1__triple_gene_block_3_[Alfalfa_virus_S]                            WSGHAFNRQ---------------------LPGVIHSD---------------------    98
YP_010798343.1__triple_gene_block_3_[Senna_severe_yellow_mosaic_virus]           WSSNASLQ---------------------------------------------------    89
QJX15397.1_triple_gene_block_protein_3_[Carnation_latent_virus]                  VANSQP-----------------------------------------------------    65
YP_010087335.1__triple_gene_block_3_[Ambrosia_asymptomatic_virus_1]              VPSYGFKFD--------------------------------------------------    76
YP_010087747.1__triple_gene_block_3_[Euonymus_yellow_mottle_associated_virus]    PITHGLSFHK---------------------SENVDFGGFTSETCCSNQ----------    83
YP_009389482.1__triple_gene_block_3_[Vanilla_virus_X]                            PLQRG------------------------------------------------------    62
YP_009091817.1__triple_gene_block_3_[Yam_virus_X]                                PLRGLSLQEF-------------------------------------------------    67
WMX21794.1__triple_gene_block_3_[Adenium_obesum_virus_X]                         AL-HAVKFPI---------------------NSE-------------------------    86
WVS18186.1__triple_gene_block_3_[Hibiscus_virus_X]                               LT--NPNFQ--------------------------------------------------    64
NP_042586.1_triple_gene_block_3_[Bamboo_mosaic_virus]                            -----------------------------------------------------------    52
NP_040991.1__triple_gene_block_3_[Foxtail_mosaic_virus]                          -----------------------------------------------------------    52
YP_009552765.1__triple_gene_block_3_[Turtle_grass_virus_X]                       -----------------------------------------------------------    55
UTI93307.1__triple_gene_block_3_[Chaenostoma_potexvirus]                         PAGFKFRQPSKSLTEHQDEFV--------------------------------------    97
NP_620839.1__triple_gene_block_3_[Plantago_asiatica_mosaic_virus]                PRGLSLEQYLKFTNTLPDGSQHRSHR---------------------------------    121
NP_702991.1__triple_gene_block_3_[Tulip_virus_X]                                 PKLLSFQTP--------------------------------------------------    94
NP_044333.1__triple_gene_block_3_[Papaya_mosaic_virus]                           AHLHGLRN---------------------------------------------------    68
YP_009448190.1__triple_gene_block_3_[Babaco_mosaic_virus]                        EALVGLRL---------------------------------------------------    68
YP_009664731.1__triple_gene_block_3_[Lagenaria_mild_mosaic_virus]                ETLARLRF---------------------------------------------------    67
YP_459947.1__triple_gene_block_3_[Alternanthera_mosaic_virus]                    DYLTGLRF---------------------------------------------------    63
YP_009270633.1__triple_gene_block_3_[Senna_mosaic_virus]                         ASLAGLRI---------------------------------------------------    67
YP_010087354.1__triple_gene_block_3_[Cnidium_virus_X]                            PISHACTERS----------------SRGWFGQQRCC----------------------    79
YP_002332932.1__triple_gene_block_3_[Potato_virus_X]                             PLSVERLSFH-------------------------------------------------    70
NP_620718.1__triple_gene_block_3_[White_clover_mosaic_virus]                     PFNHGLSLPSN------------------------------------------------    66
NP_054028.1_triple_gene_block_3_[Cymbidium_mosaic_virus]                         PW-RGLSYHN-NLK---------------------------------------------    91
YP_446995.1__triple_gene_block_3_[Nerine_virus_X]                                NHVRGLIKP-------ELE-------------IEPHCSYGKH-----------------    120
YP_224137.1__triple_gene_block_3_[Mint_virus_X]                                  ------SRFIRNHD---------------------------------------------    95
YP_263306.1__triple_gene_block_3_[Lily_virus_X]                                  PASGLSCQKFRNHHYDDL------------------CS---------------------    78
YP_009389422.1__triple_gene_block_3_[Euonymus_yellow_vein_virus]                 PHTHGLSYQ--DSKVQDVSLESRMVRRPGTRRQDKDCGF--------------------    93
YP_009124991.1__triple_gene_block_3_[Citrus_yellow_vein_clearing_virus]          PAKPV------------------------------------------------------    60
YP_010798311.1__triple_gene_block_3_[Citrus_yellow_mottle_virus]                 PAKPV------------------------------------------------------    60
NP_203556.1__triple_gene_block_3_[Indian_citrus_ringspot_virus]                  PAKPV------------------------------------------------------    60
```

Figure S5. Amino acid alignment of triple gene block 3 (TGB3) sequences of members of the family *Alphaflexiviridae*. Multiple sequence alignment of 54 TGB3 sequences using Clustal Omega.
